# Supplementary figures and images for: Patterns of primates crop foraging and the impacts on incomes of smallholders across the mosaic agricultural landscape of Wolaita zone, southern Ethiopia
Source: PLoS One. 2024 Nov 18;19(11):e0313831. doi: 10.1371/journal.pone.0313831 (PMC11573158; doi:10.1371/journal.pone.0313831)

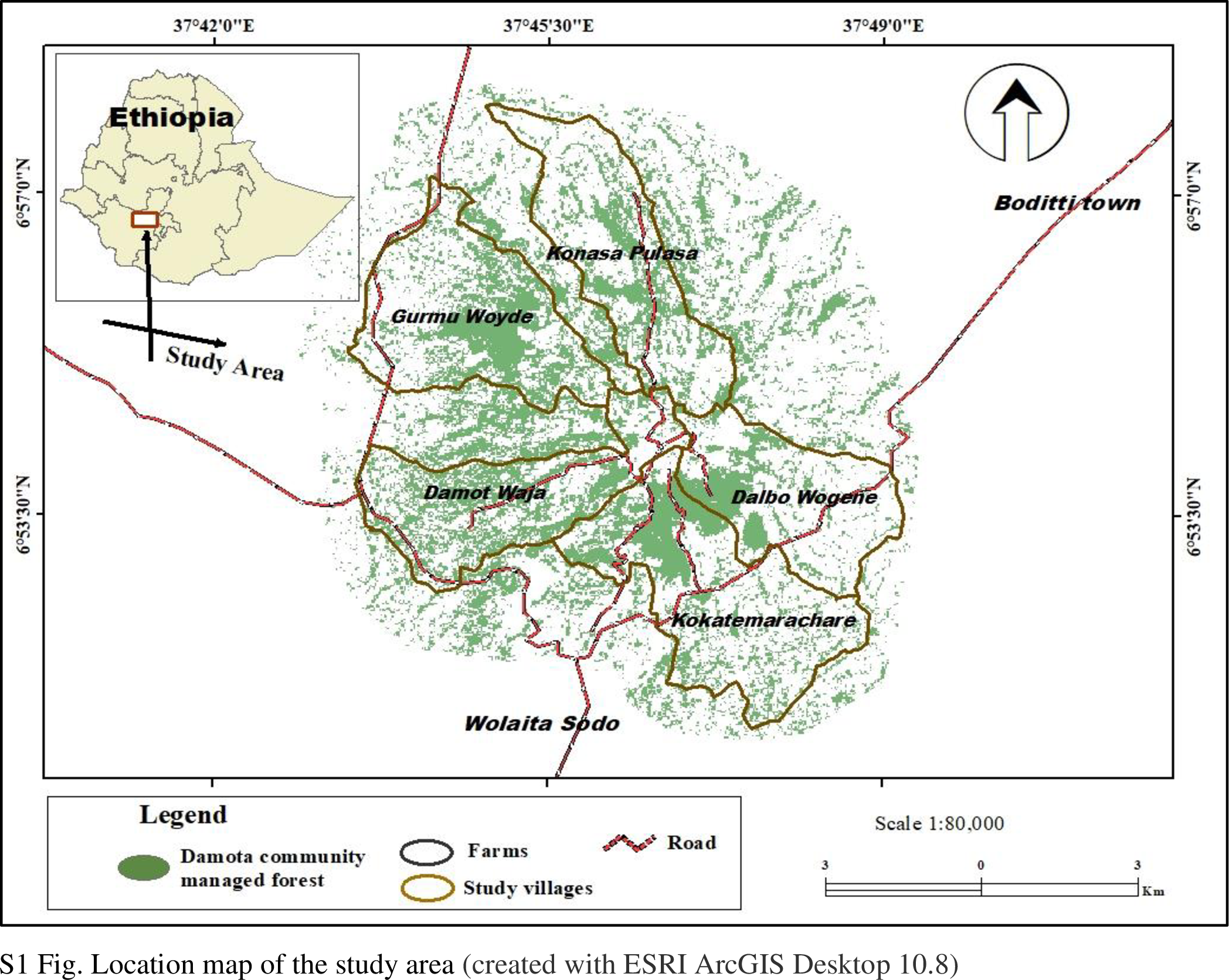

Supplement: S1 Fig — (TIF) [file pone.0313831.s001.tif]

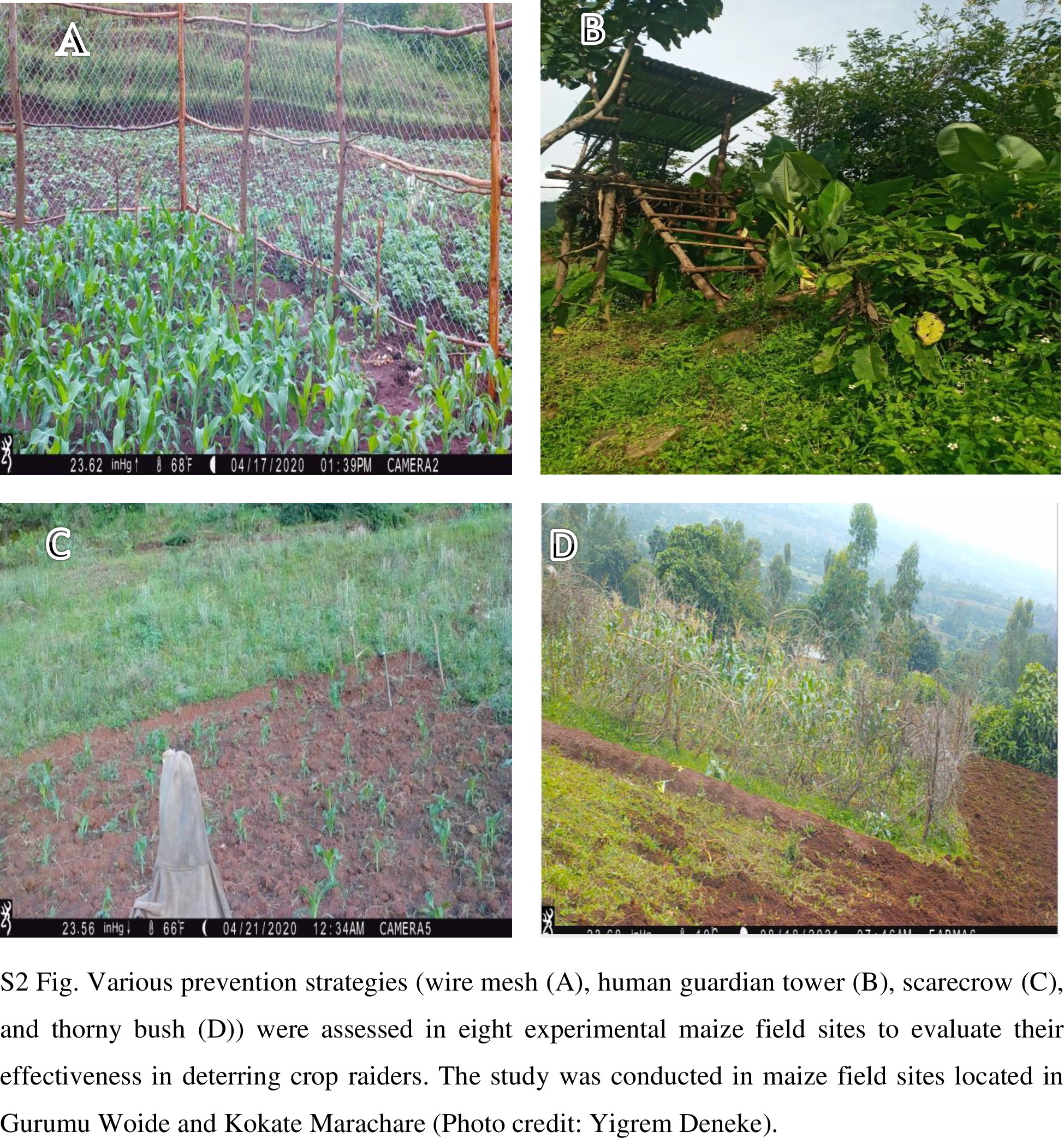

Supplement: S2 Fig — Various prevention strategies (wire mesh (A), human guardian tower (B), scarecrow (C), and thorny bush (D)) were assessed in eight experimental maize field sites to evaluate their effectiveness in deterring crop raiders. The study was conducted in maize field sites located in Gurumu Woide and Kokate Marachare (Photo credit: Yigrem Deneke). (TIF) [file pone.0313831.s002.tif]

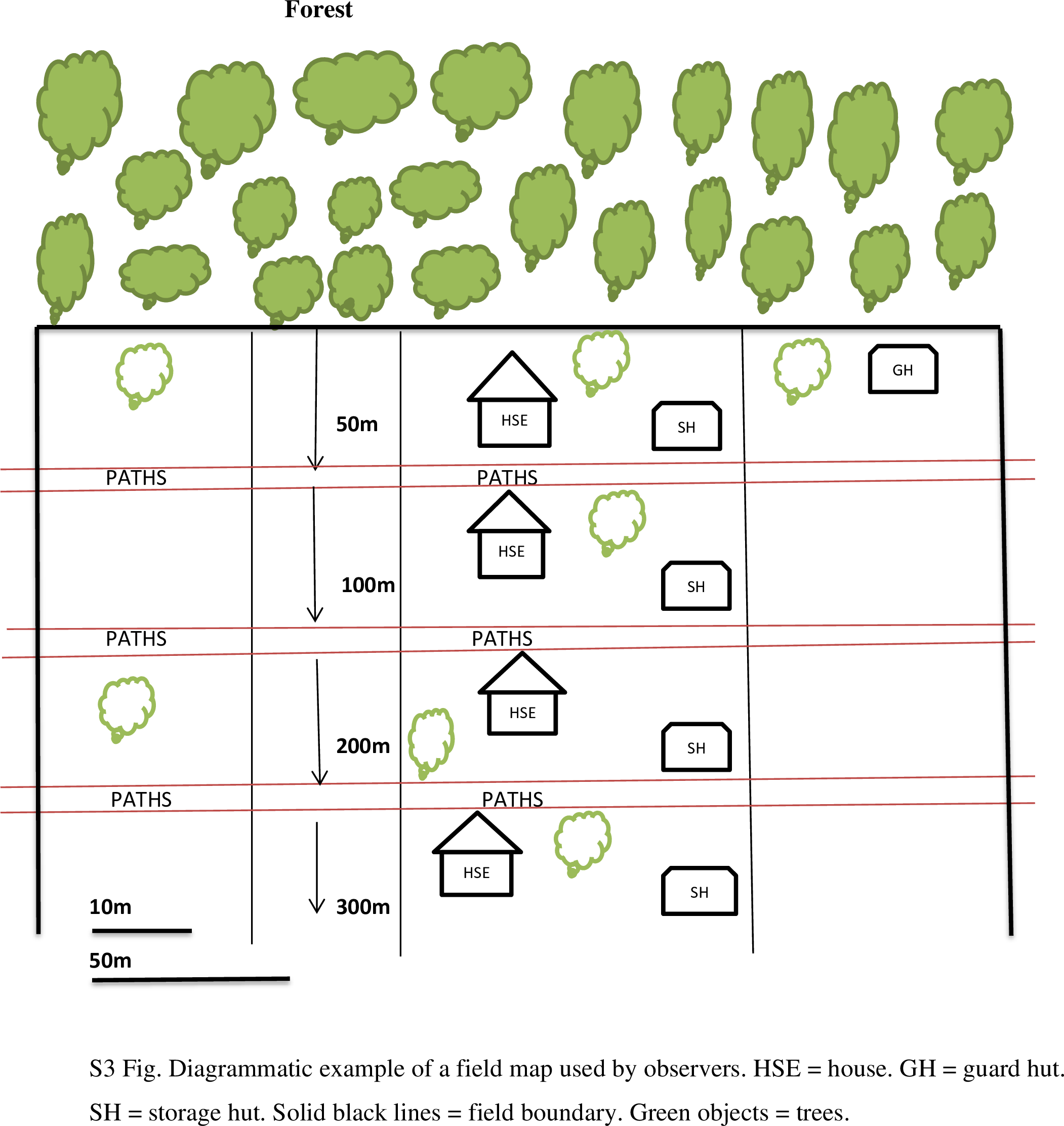

Supplement: S3 Fig — HSE = house. GH = guard hut. SH = storage hut. Solid black lines = field boundary. Green objects = trees. (TIF) [file pone.0313831.s003.tif]

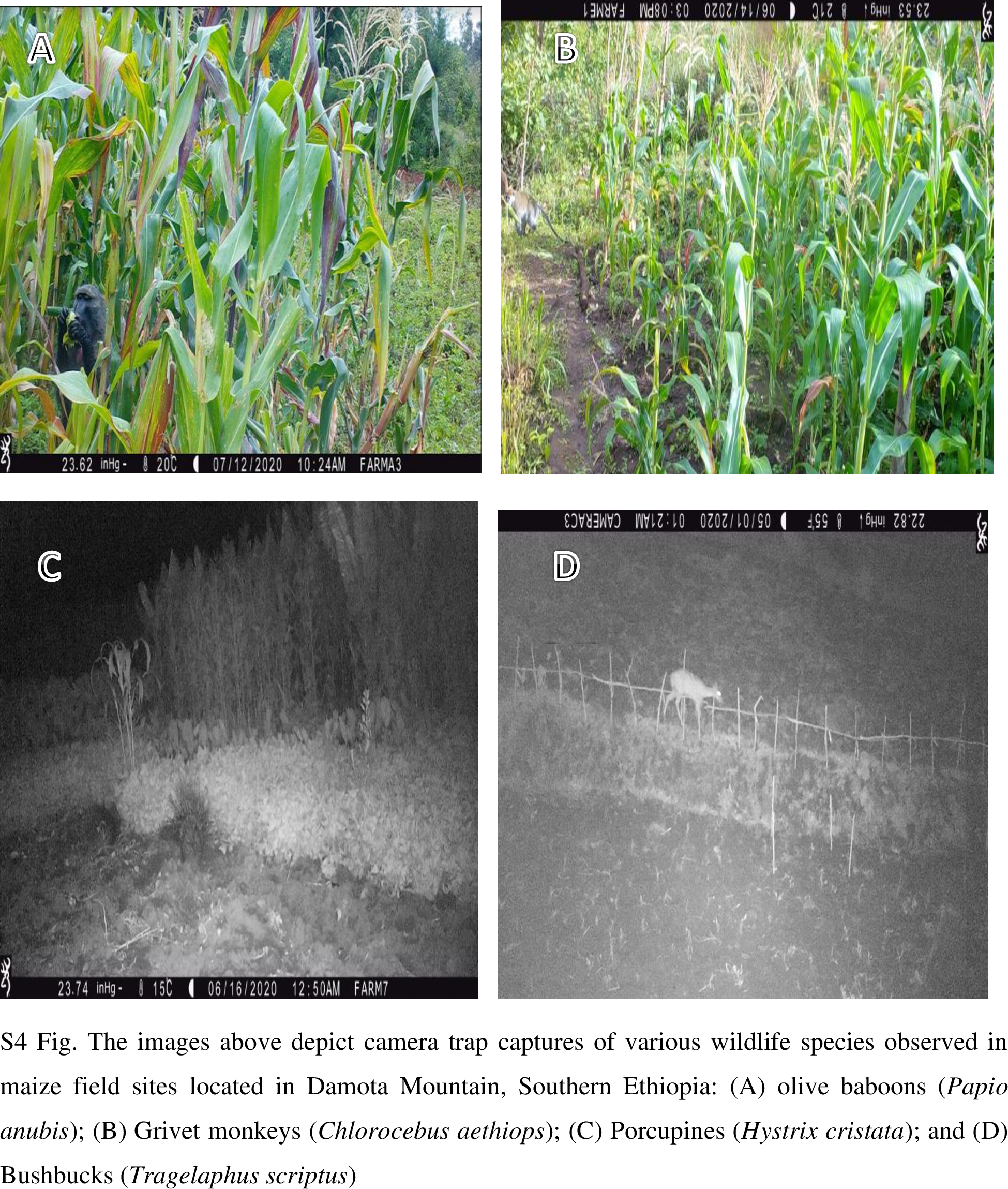

Supplement: S4 Fig — The images above depict camera trap captures of various wildlife species observed in maize field sites located in Damota Mountain, Southern Ethiopia: (A) Olive baboons (Papio anubis); (B) Grivet monkeys (Chlorocebus aethiops); (C) Porcupines (Hystrix cristata); and (D) Bushbucks (Tragelaphus scriptus). (TIF) [file pone.0313831.s004.tif]

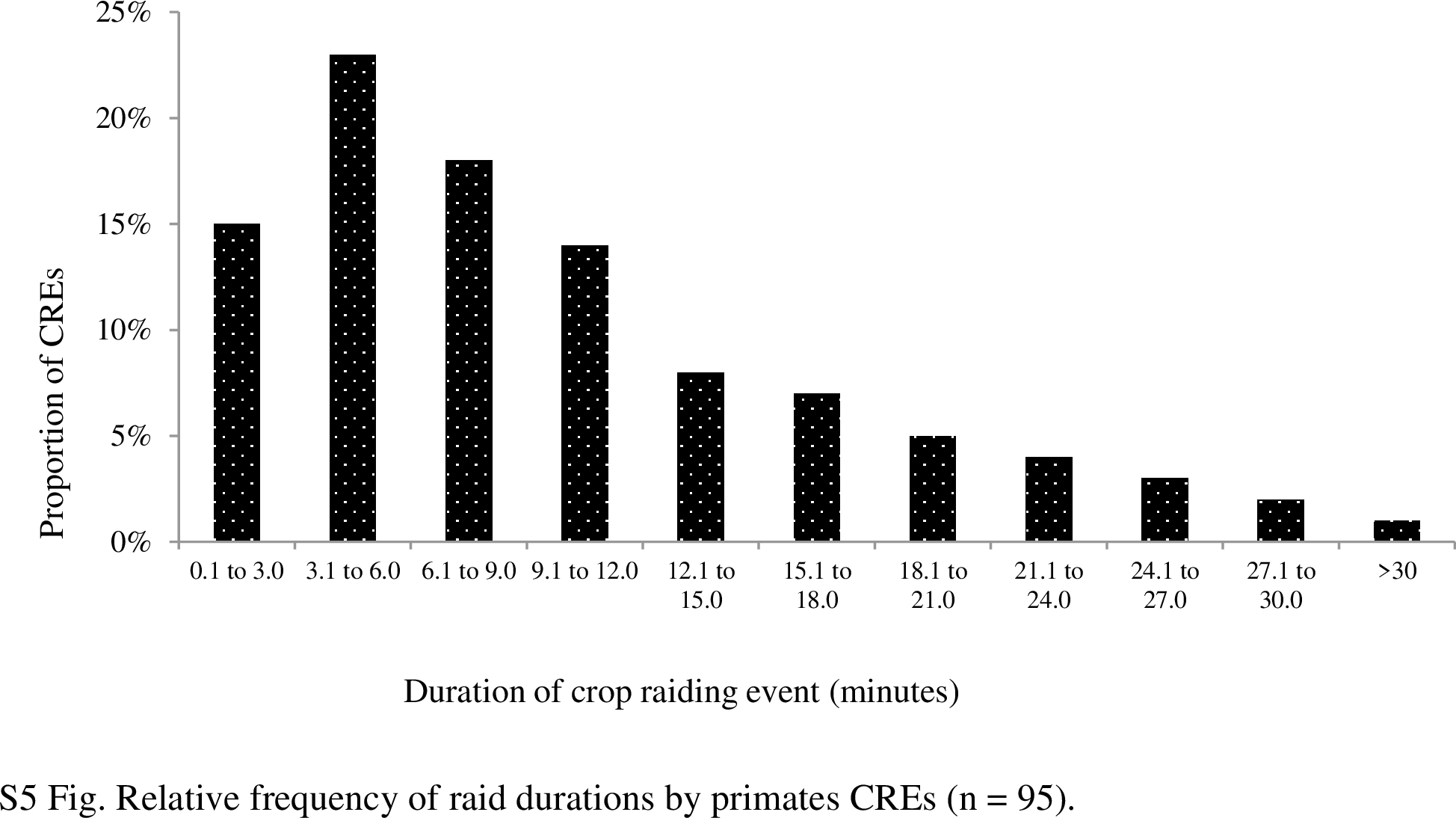

Supplement: S5 Fig — (TIF) [file pone.0313831.s005.tif]

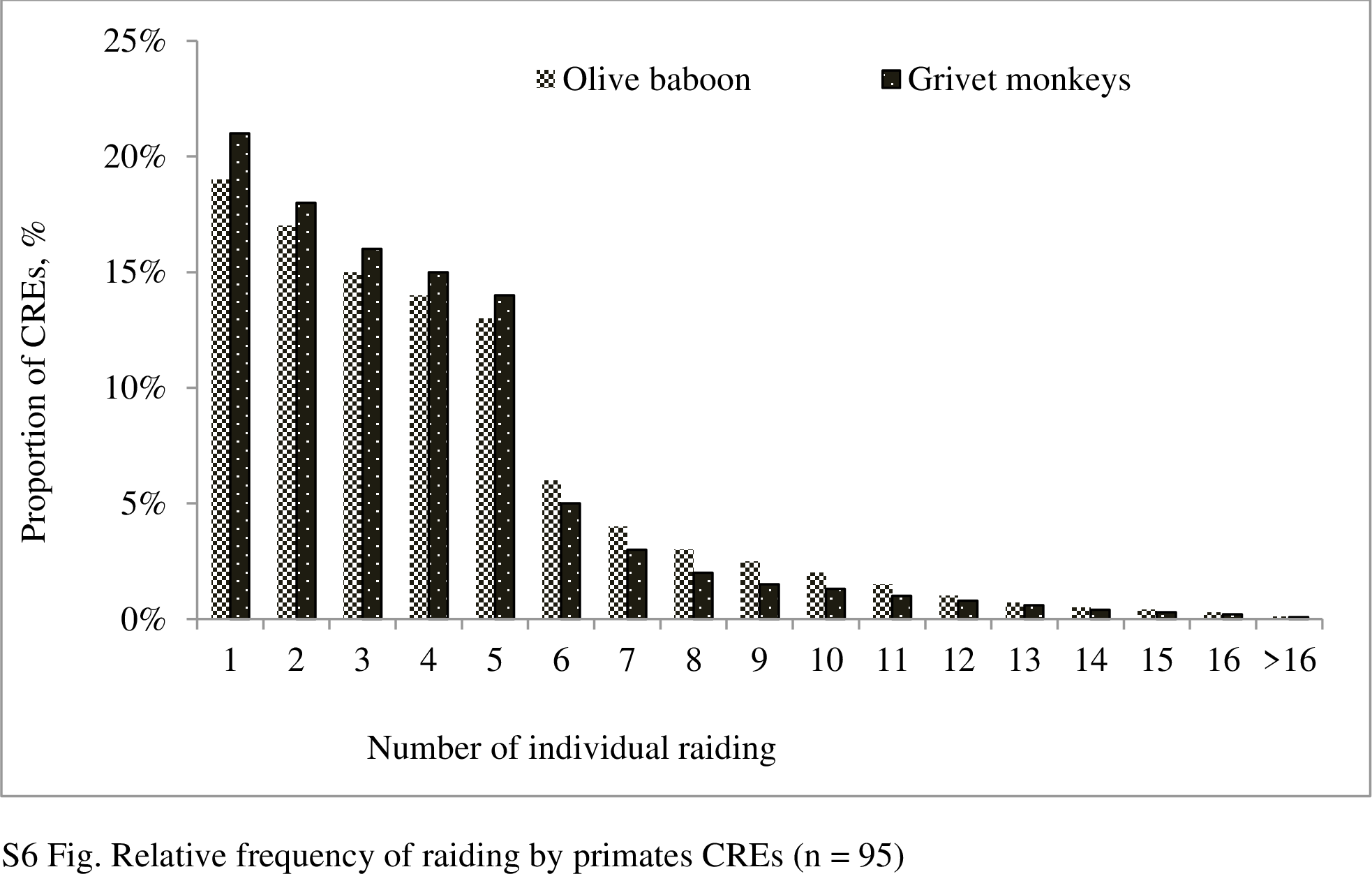

Supplement: S6 Fig — (TIF) [file pone.0313831.s006.tif]

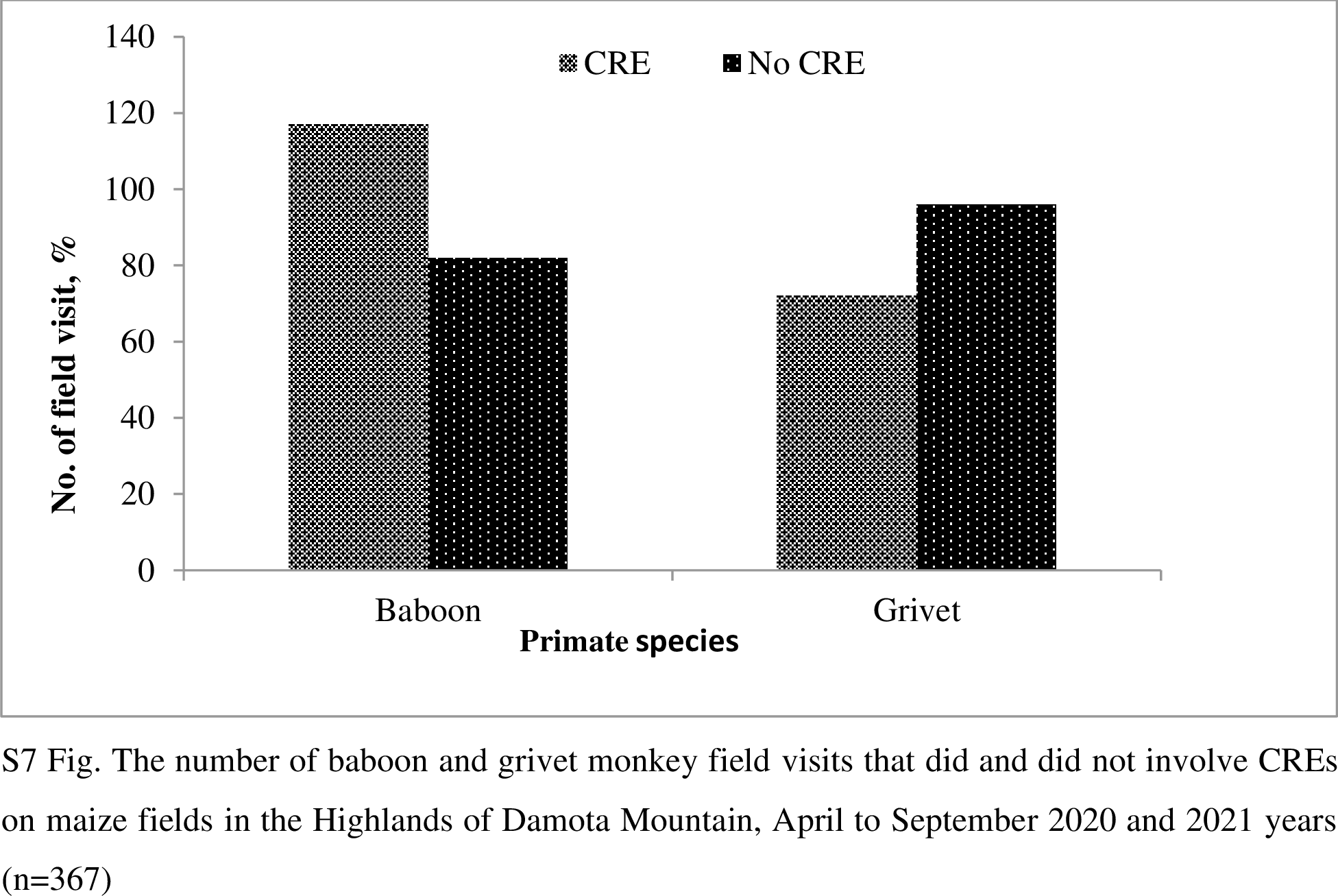

Supplement: S7 Fig — (TIF) [file pone.0313831.s007.tif]

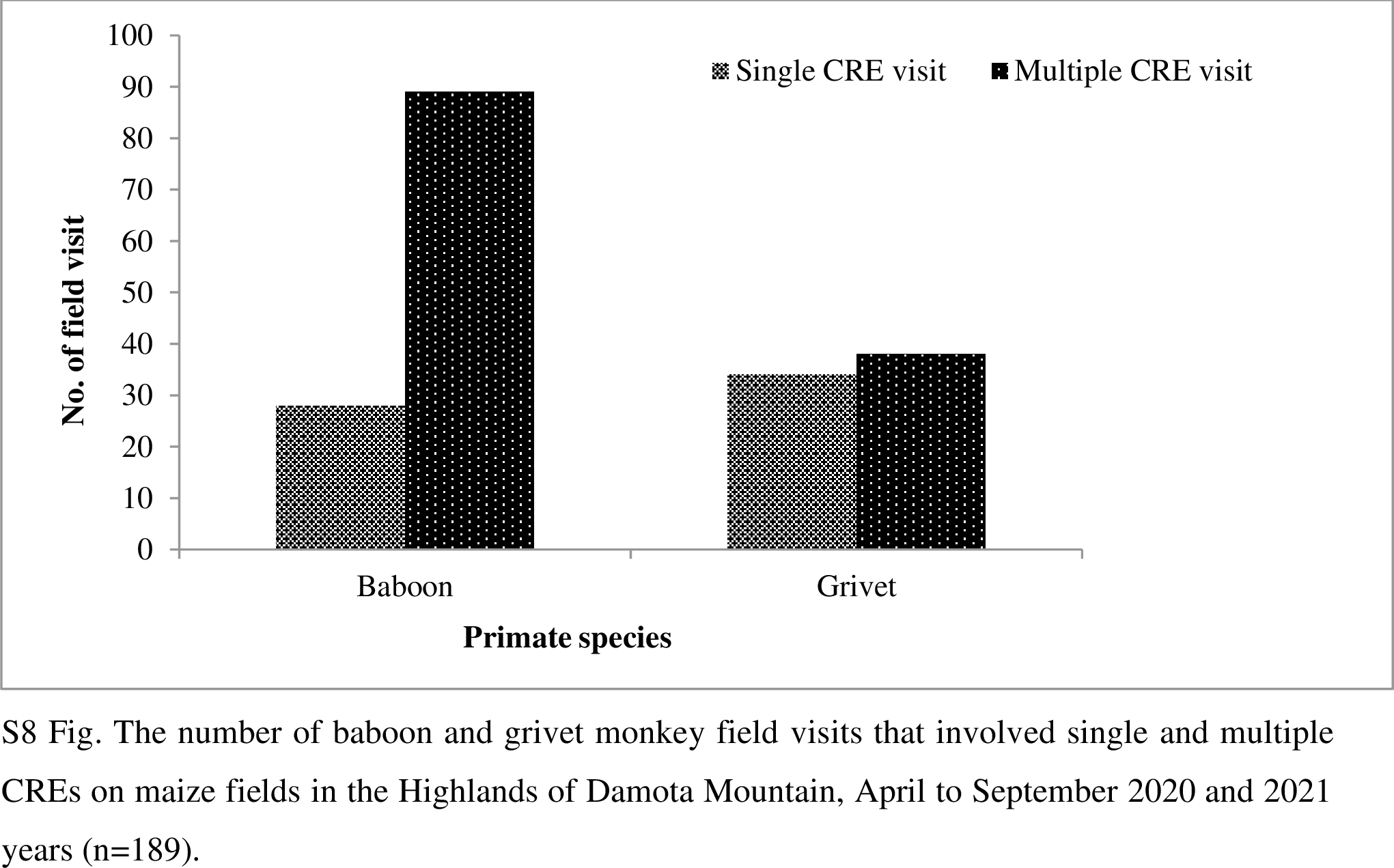

Supplement: S8 Fig — (TIF) [file pone.0313831.s008.tif]
