# Supplementary material for: Patterns of primates crop foraging and the impacts on incomes of smallholders across the mosaic agricultural landscape of Wolaita zone, southern Ethiopia
Source: PLoS One. 2024 Nov 18;19(11):e0313831. doi: 10.1371/journal.pone.0313831 (PMC11573158; doi:10.1371/journal.pone.0313831)
Supplement: S1 Table — (DOCX) [file pone.0313831.s009.docx]

S1 Table. Maize field and study plot size on the protective and non-protective maize fields

|  |  |  |  | | |
| --- | --- | --- | --- | --- | --- |
| Study sites | Field number | Maize field size in hectare | Study plot size (10x10m) | Distance to forest edge | Preventive and non-preventive measures |
| Gurumu Woide | 1 | 0.01 | 0.01 | 50m | Wire mesh |
|  | 2 | 0.06 | 0.01 | 50m | Human guard |
|  | 3 | 0.1 | 0.01 | 50m | Scarecrow |
|  | 4 | 0.1 | 0.01 | 50m | Thorny bushy |
|  | 5 | 0.1 | 0.01 | 50m | Open/control |
|  | 6 | 0.2 | 0.01 | 100m | Open |
|  | 7 | 0.3 | 0.01 | 200m | Open |
|  | 8 | 0.3 | 0.01 | 300m | Open |
| Kokate Marachare | 9 | 0.2 | 0.01 | 50m | Wire mesh |
|  | 10 | 0.2 | 0.01 | 50m | Scarecrow |
|  | 11 | 0.2 | 0.01 | 50m | Thorny bushy |
|  | 12 | 0.2 | 0.01 | 50m | Open/control |
|  | 13 | 0.2 | 0.01 | 50m | Human guard |
|  | 14 | 0.3 | 0.01 | 100m | Open |
|  | 15 | 0.3 | 0.01 | 200m | Open |
|  | 16 | 0.3 | 0.01 | 300m | Open |
| Delbo Wogene | 17 | 0.2 | 0.01 | 100m | Open |
|  | 18 | 0.2 | 0.01 | 200m | Open |
|  | 19 | 0.3 | 0.01 | 300m | Open |
| Damot Waja | 20 | 0.06 | 0.01 | 100m | Open |
|  | 21 | 0.3 | 0.01 | 200m | Open |
|  | 22 | 0.3 | 0.01 | 300m | Open |
| Konasa Pulasa | 23 | 0.01 | 0.01 | 100m | Open |
|  | 24 | 0.3 | 0.01 | 200m | Open |
|  | 25 | 0.3 | 0.01 | 300m | Open |
