# Supplementary material for: Patterns of primates crop foraging and the impacts on incomes of smallholders across the mosaic agricultural landscape of Wolaita zone, southern Ethiopia
Source: PLoS One. 2024 Nov 18;19(11):e0313831. doi: 10.1371/journal.pone.0313831 (PMC11573158; doi:10.1371/journal.pone.0313831)
Supplement: S2 Table — (DOCX) [file pone.0313831.s010.docx]

S2 Table. Farmer observation and reported of maize damage assessments (580 maize stem expected per plot except field no. 25 (see the text)

| Study sites | Field number | Distance to forest | measures | Olive baboons | | | | | | Grivet monkeys | | | | | |
| --- | --- | --- | --- | --- | --- | --- | --- | --- | --- | --- | --- | --- | --- | --- | --- |
|  |  |  |  | Maize cobs loss | | % damaged | | Av. damaged | Av.% | Maize cobs loss | | % damaged | | Av. damaged | Av. % |
|  |  |  |  | 2020 | 2021 | 2020 | 2021 | 2020/21 | 2020/21 | 2020 | 2021 | 2020 | 2021 | 2020/21 | 2020/21 |
| Gurumu Woide | 1 | 50m | Wire mesh | 145 | 146 | 16.57 | 16.35 | 145.5 | 16.46 | 0 | 0 | 0 | 0 | 0 | 0 |
|  | 2 | 50m | guard | 127 | 128 | 14.51 | 14.33 | 127.5 | 14.42 | 0 | 0 | 0 | 0 | 0 | 0 |
|  | 3 | 50m | Scarecrow | 165 | 167 | 18.86 | 18.7 | 166 | 18.78 | 0 | 0 | 0 | 0 | 0 | 0 |
|  | 4 | 50m | Thorny | 160 | 161 | 18.29 | 18.03 | 160.5 | 18.16 | 0 | 0 | 0 | 0 | 0 | 0 |
|  | 5 | 50m | Open/control | 164 | 168 | 18.74 | 18.81 | 166 | 18.78 | 0 | 0 | 0 | 0 | 0 | 0 |
|  | 6 | 100m | Open | 48 | 54 | 5.48 | 6.05 | 51 | 5.77 | 0 | 0 | 0 | 0 | 0 | 0 |
|  | 7 | 200m | Open | 16 | 13 | 1.83 | 1.46 | 14.5 | 1.64 | 0 | 0 | 0 | 0 | 0 | 0 |
|  | 8 | 300m | Open | 4 | 5 | 0.46 | 0.56 | 4.5 | 0.51 | 0 | 0 | 0 | 0 | 0 | 0 |
| Kokate Marachare | 9 | 50m | Wire mesh | 0 | 0 | 0 | 0 | 0 | 0 | 0 | 0 | 0 | 0 | 0 | 0 |
|  | 10 | 50m | guard | 4 | 2 | 0.46 | 0.22 | 3 | 0.34 | 12 | 13 | 3.56 | 3.74 | 12.5 | 3.65 |
|  | 11 | 50m | Scarecrow | 7 | 8 | 0.8 | 0.9 | 7.5 | 0.85 | 25 | 27 | 7.42 | 7.76 | 26 | 7.59 |
|  | 12 | 50m | Thorny | 6 | 7 | 0.69 | 0.78 | 6.5 | 0.74 | 17 | 19 | 5.04 | 5.46 | 18 | 5.26 |
|  | 13 | 50m | Open/control | 11 | 12 | 1.26 | 1.34 | 11.5 | 1.3 | 29 | 31 | 8.61 | 8.91 | 30 | 8.76 |
|  | 14 | 100m | Open | 15 | 18 | 1.71 | 2.02 | 16.5 | 1.86 | 11 | 12 | 3.26 | 3.44 | 11.5 | 3.36 |
|  | 15 | 200m | Open | 3 | 4 | 0.34 | 0.45 | 3.5 | 0.39 | 2 | 5 | 0.59 | 1.43 | 3.5 | 1.02 |
|  | 16 | 300m | Open | 0 | 0 | 0 | 0 | 0 | 0 | 0 | 0 | 0 | 0 | 0 | 0 |
| Delbo Wogene | 17 | 100m | Open | 0 | 0 | 0 | 0 | 0 | 0 | 30 | 28 | 8.9 | 8.04 | 29 | 8.47 |
|  | 18 | 200m | Open | 0 | 0 | 0 | 0 | 0 | 0 | 9 | 12 | 2.67 | 3.44 | 10.5 | 3.07 |
|  | 19 | 300m | Open | 0 | 0 | 0 | 0 | 0 | 0 | 0 | 0 | 0 | 0 | 0 | 0 |
| Damot Waja | 20 | 100m | Open | 0 | 0 | 0 | 0 | 0 | 0 | 40 | 42 | 11.9 | 12.1 | 41 | 11.97 |
|  | 21 | 200m | Open | 0 | 0 | 0 | 0 | 0 | 0 | 10 | 10 | 2.97 | 2.87 | 10 | 2.92 |
|  | 22 | 300m | Open | 0 | 0 | 0 | 0 | 0 | 0 | 0 | 0 | 0 | 0 | 0 | 0 |
| Konasa Pulasa | 23 | 100m | Open | 0 | 0 | 0 | 0 | 0 | 0 | 117 | 119 | 34.7 | 34.19 | 118 | 34.45 |
|  | 24 | 200m | Open | 0 | 0 | 0 | 0 | 0 | 0 | 35 | 30 | 10.4 | 8.62 | 32.5 | 9.48 |
|  | 25 | 300m | Open | 0 | 0 | 0 | 0 | 0 | 0 | 0 | 0 | 0 | 0 | 0 | 0 |
| Total |  | | | 875 | 893 | 100 | 100 | 884 | 100 | 337 | 348 | 100 | 100 | 342.5 | 100 |
