# Supplementary material for: Patterns of primates crop foraging and the impacts on incomes of smallholders across the mosaic agricultural landscape of Wolaita zone, southern Ethiopia
Source: PLoS One. 2024 Nov 18;19(11):e0313831. doi: 10.1371/journal.pone.0313831 (PMC11573158; doi:10.1371/journal.pone.0313831)
Supplement: S3 Table — Each field comprised study plots measuring 10mx10 meters, observed during the maize cropping seasons of 2020 and 2021. (DOCX) [file pone.0313831.s011.docx]

S3 Table. Camera traps recorded CREs and CFEs of olive baboons and grivet monkeys among twenty-five selected maize fields. Each field comprised study plots measuring 10mx10 meters, observed during the maize cropping seasons of 2020 and 2021.

| Study sites | Farm code | Prevention method | Distance farthest from forest edge | Date of camera trap | Time of primate CRE/CFE events | Number of primate image recorded | Olive baboon CRE | Olive baboon CFE | Grivets monkeys CRE | Grivets monkeys CFE |
| --- | --- | --- | --- | --- | --- | --- | --- | --- | --- | --- |
| Gurumu Woide | Farm A1 | Wire mesh | 50m | 6/8/2020 | 5:22am | Olive baboon (1) | 1 | 0 | 0 | 0 |
|  | Farm A2 | Human Guardian | 50m | 6/12/2020 | 6:49am | Olive baboon (1) | 1 | 0 | 0 | 0 |
|  | Farm A3 | Scarecrow | 50m | 4/21/2020 | 2:40pm | Olive baboon (1) | 1 | 0 | 0 | 0 |
|  |  | Scarecrow | 50m | 7/12/2020 | 10:24am | Olive baboon (1) | 0 | 1 | 0 | 0 |
|  | Farm A4 | Thorny bush | 50m | 5/4/2020 | 3:40pm | Olive baboon (1) | 1 | 0 | 0 | 0 |
|  | Farm A5 | Open/control | 50m | 6/11/2020 | 6:49am | Olive baboon (1) | 1 | 0 | 0 | 0 |
|  |  | Open/control | 50m | 6/11/2020 | 8:46am | Olive baboon (1) | 1 | 0 | 0 | 0 |
|  |  | Open/control | 50m | 6/11/2020 | 10:48am | Olive baboon (1) | 1 | 0 | 0 | 0 |
|  |  | Open/control | 50m | 6/11/2020 | 1:32pm | Olive baboon (1) | 1 | 0 | 0 | 0 |
|  |  | Open/control | 50m | 6/11/2020 | 2:34pm | Olive baboon (1) | 1 | 0 | 0 | 0 |
|  | Farm A6 | Open | 100m | 10/11/2020 | 3:48pm | Olive baboon (1) | 1 | 0 | 0 | 0 |
| Kokate Marachere | FarmB1 | Wire mesh | 50m | 4/21/2020 | 3:50pm | Olive baboon (1) | 1 | 0 | 0 | 0 |
|  | Farm B6 | Open | 100m | 4/22/2020 | 8:00am | Olive baboon (1) | 1 | 0 | 0 | 0 |
|  |  | Open | 100m | 4/23/2020 | 5:14pm | Grivet monkey (1) | 0 | 0 | 1 | 0 |
| Delbo Wogene | Farm C1 | Open | 100m | 5/2/2020 | 6:30am | Grivet monkey (1) | 0 | 0 | 1 | 0 |
| Damot Waja | Farm D1 | Open | 100m | 4/28/2020 | 6:25am | Grivet monkey (1) | 0 | 0 | 1 | 0 |
| Konasa Pulasa | Farm E1 | Open | 100m | 6/14/2020 | 3:08pm | Grivet monkey (2) | 0 | 0 | 1 | 2 |
|  |  | Open | 100m | 6/14/2020 | 2:57pm | Grivet monkey (1) | 0 | 0 | 1 | 0 |
|  | Farm E2 | Open | 200m | 6/14/2020 | 8:10am | Grivet monkey(1) | 0 | 0 | 1 | 0 |
| Gurumu Woide | Farm A1 | Wire mesh | 50m | 8/11/2021 | 4:18pm | Olive baboon (1) | 1 | 0 | 0 | 0 |
|  | Farm A2 | Human Guardian | 50m | 9/8/2021 | 12:44pm | Olive baboon (1) | 1 | 0 | 0 | 0 |
|  | Farm A3 | Human Guardian | 50m | 9/10/2021 | 3:25pm | Olive baboon (1) | 1 | 0 | 0 | 0 |
|  |  | Human Guardian | 50m | 9/11/2021 | 2:02pm | Olive baboon (1) | 1 | 0 | 0 | 0 |
|  |  | Scarecrow | 50m | 11/17/2021 | 7:50am | Olive baboon (1) | 1 | 0 | 0 | 0 |
|  |  | Scarecrow | 50m | 11/18/2021 | 7:04am | Olive baboon (2) | 1 | 1 | 0 | 0 |
|  |  | Scarecrow | 50m | 11/19/2021 | 5:18pm | Olive baboon (1) | 1 | 1 | 0 | 0 |
|  |  | Scarecrow | 50m | 11/23/2021 | 8:06am | Olive baboon (1) | 1 | 0 | 0 | 0 |
|  |  | Scarecrow | 50m | 11/26/2021 | 7:15am | Olive baboon (1) | 1 | 0 | 0 | 0 |
|  |  | Scarecrow | 50m | 11/26/2021 | 9:26am | Olive baboon (1) | 1 | 0 | 0 | 0 |
|  |  | Scarecrow | 50m | 11/26/2021 | 10:30am | Olive baboon (1) | 1 | 0 | 0 | 0 |
|  |  | Scarecrow | 50m | 11/26/2021 | 11:32am | Olive baboon (1) | 1 | 0 | 0 | 0 |
|  |  | Scarecrow | 50m | 11/26/2021 | 12:34am | Olive baboon (1) | 1 | 0 | 0 | 0 |
|  |  | Scarecrow | 50m | 11/26/2021 | 7:35am | Olive baboon (1) | 1 | 0 | 0 | 0 |
|  | Farm A4 | Thorny bush | 50m | 8/10/2021 | 12:40am | Olive baboon (1) | 1 | 0 | 0 | 0 |
|  |  | Thorny bush | 50m | 8/11/2021 | 9:17am | Olive baboon (1) | 1 | 0 | 0 | 0 |
|  |  | Thorny bush | 50m | 8/12/2021 | 7:22am | Olive baboon (1) | 1 | 0 | 0 | 0 |
|  |  | Thorny bush | 50m | 8/12/2021 | 8:25am | Olive baboon (1) | 1 | 0 | 0 | 0 |
|  |  | Thorny bush | 50m | 8/13/2021 | 6:17am | Olive baboon (1) | 1 | 0 | 0 | 0 |
|  | Farm A6 | Open | 100m | 8/14/2021 | 3:07pm | Olive baboon (1) | 1 | 0 | 0 | 0 |
|  | Farm A6 | Open | 100m | 8/15/2021 | 8:37am | Olive baboon (1) | 1 | 0 | 0 | 0 |
| Kokate Marachere | Farm B1 | Wire mesh | 50m | 6/15/2021 | 5:29am | Olive baboon (1) | 1 | 0 | 0 | 0 |
|  | Farm B2 | Scarecrow | 50m | 6/16/2021 | 6:15am | Grivet monkey (1) | 0 | 0 | 1 | 0 |
|  | Farm B3 | Thorny bush | 50m | 6/17/2021 | 8:00am | Grivet monkey (1) | 0 | 0 | 1 | 0 |
|  |  | Thorny bush | 50m | 6/18/2021 | 4:25pm | Olive baboon (1) | 1 | 0 | 0 | 0 |
|  | Farm B4 | Open/control | 50m | 6/24/2021 | 7:21am | Grivet monkey (1) | 0 | 0 | 1 | 0 |
|  |  | Open/control | 50m | 6/21/2021 | 5:20pm | Olive baboon (1) | 1 | 0 | 0 | 0 |
|  |  | Open/control | 50m | 6/22/2021 | 7:24am | Olive baboon (1) | 1 | 0 | 0 | 0 |
|  |  | Open/control | 50m | 6/23/2021 | 12:03am | Olive baboon (1) | 1 | 0 | 0 | 0 |
|  | Farm B5 | Human Guardian | 50m | 6/22/2021 | 12:30am | Olive baboon (1) | 1 | 0 | 0 | 0 |
|  |  | Human Guardian | 50m | 6/22/2021 | 4:48pm | Olive baboon (1) | 1 | 0 | 0 | 0 |
|  |  | Human Guardian | 50m | 6/22/2021 | 5:26pm | Olive baboon (1) | 1 | 0 | 0 | 0 |
|  |  | Human Guardian | 50m | 6/22/2021 | 5:52pm | Olive baboon (1) | 1 | 0 | 0 | 0 |
|  |  | Human Guardian | 50m | 6/23/2021 | 12:05am | Olive baboon (1) | 1 | 0 | 0 | 0 |
|  |  | Human Guardian | 50m | 6/24/2021 | 7:55am | Olive baboon (1) | 1 | 0 | 0 | 0 |
| Konasa Pulasa | Farm E1 | Open | 100m | 6/10/2021 | 6:30am | Grivet monkey (1) | 0 | 0 | 1 | 0 |
|  |  | Open | 100m | 9/19/2021 | 2:07pm | Grivet monkey (1) | 0 | 0 | 1 | 0 |
|  |  | Open | 100m | 9/22/2021 | 1:37pm | Grivet monkey (1) | 0 | 0 | 1 | 0 |
|  |  | Open | 100m | 9/22/2021 | 3:24pm | Grivet monkey (1) | 0 | 0 | 1 | 0 |
|  |  | Open | 100m | 12/15/2021 | 7:09am | Grivet monkey (1) | 0 | 0 | 1 | 0 |
|  |  | Open | 100m | 12/15/2021 | 10:41am | Grivet monkey (1) | 0 | 0 | 1 | 0 |
|  |  | Open | 100m | 9/19/2021 | 6:05am | Grivet monkey (2) | 0 | 0 | 1 | 0 |
|  |  | Open | 100m | 9/20/2021 | 8:28am | Grivet monkey (1) | 0 | 0 | 1 | 0 |
|  |  | Open | 100m | 9/20/2021 | 8:29am | Grivet monkey (1) | 0 | 0 | 1 | 0 |
|  |  | Open | 100m | 9/22/2021 | 1:37pm | Grivet monkey (1) | 0 | 0 | 1 | 0 |
|  | Farm E2 | Open | 200m | 9/23/2021 | 6:50am | Grivet monkey (1) | 0 | 0 | 0 | 0 |
| Total | |  |  |  |  |  | 44 | 3 | 19 | 2 |
