# Supplementary material for: Patterns of primates crop foraging and the impacts on incomes of smallholders across the mosaic agricultural landscape of Wolaita zone, southern Ethiopia
Source: PLoS One. 2024 Nov 18;19(11):e0313831. doi: 10.1371/journal.pone.0313831 (PMC11573158; doi:10.1371/journal.pone.0313831)
Supplement: S4 Table — (DOCX) [file pone.0313831.s012.docx]

S4 Table. A linear mixed model of the maize damage rate by primates, considering different spatio-temporal variables, was analyzed using R code.

lmm_model <- lmer(Maize_SD ~ distance_farm + durationof_raiding +

+ crop_p + (1|PrimateCREs)+ (1|numberofindividualsraiding),data=maize)

> summary(lmm_model)

Linear mixed model fit by REML. t-tests use Satterthwaite's method ['lmerModLmerTest']

Formula: Maize_SD ~ distance_farm + durationof_raiding + crop_phenology + (1 |

PrimateCREs) + (1 | numberofindividualsraiding)

Data: maize

REML criterion at convergence: 2135.2

Scaled residuals:

Min 1Q Median 3Q Max

-2.5734 -0.5770 -0.1952 0.4354 3.1467

**Random effects:**

Groups Name Variance Std.Dev.

Numberofindividualsraiding (Intercept) 2.652 1.629

PrimateCREs (Intercept) 17.234 4.151

Residual 190.220 13.792

Number of obs: 274, groups: numberofindividualsraiding, 17; PrimateCREs, 5

**Fixed effects:**

|  |
| --- |

Parameters Estimate Std. Error df t value Pr(>|t|)

|  |
| --- |

(Intercept) 66.646 4.424 30.611 15.064 1.06e-15 *******

distance_farm100m -1.848 2.004 256.286 -0.922 0.357

distance_farm200m -10.088 3.698 256.976 -2.728 0.007 ******

distance_farm300m -6.388 4.196 257.913 -1.523 0.129

durationof_raiding3.1-6 minute -3.276 2.312 257.931 -1.417 0.158

durationof_raiding6.1-9 minute -6.466 3.244 182.907 -1.993 0.048 *****

durationof_raiding9.1-12 minute -3.517 4.119 217.458 -0.854 0.394

durationof_raiding12.1-15 minute -7.025 5.300 147.578 -1.325 0.187

durationof_raiding15.1-18 minute -9.031 5.434 218.392 -1.662 0.098 .

durationof_raiding18.1-21 minute -6.752 6.370 232.020 -1.060 0.290

durationof_raiding21.1-24 minute -8.664 6.813 248.224 -1.272 0.205

durationof_raiding24.1-27 minute -11.756 7.637 245.037 -1.539 0.125

durationof_raiding27.1-30 minute -11.639 8.685 228.281 -1.340 0.182

durationof_raiding>30 minute -8.555 10.282 227.031 -0.832 0.406

crop_phenology_fruiting -46.620 3.999 98.983 -11.656 < 2e-16 *******

crop_phenology_maturity -55.256 4.084 176.050 -13.530 < 2e-16 *******

|  |
| --- |

Signif. codes: 0 ‘***’ 0.001 ‘**’ 0.01 ‘*’ 0.05 ‘.’ 0.1 ‘ ’ 1

Residual standard error: 14.24 on 258 degrees of freedom

Multiple R-squared: 0.4802, Adjusted R-squared: 0.45

F-statistic: 15.89 on 15 and 258 DF, p-value: < 2.2e-16
