# Supplementary material for: Patterns of primates crop foraging and the impacts on incomes of smallholders across the mosaic agricultural landscape of Wolaita zone, southern Ethiopia
Source: PLoS One. 2024 Nov 18;19(11):e0313831. doi: 10.1371/journal.pone.0313831 (PMC11573158; doi:10.1371/journal.pone.0313831)
Supplement: S1 File — (DOCX) [file pone.0313831.s013.docx]

S1 File. The rate of maize damage by olive baboons in different crop phonological stages was analyzed in both protected and open/control fields using R code.

Maize damage by olive baboons at Gurumu Woide site

v=c (0, 67, 6) (0=seedling stage, 67= fruiting stage, 6= maturity stage)

w= c (0, 61, 4) (0=seedling stage, 61= fruiting stage, 4= maturity stage)

x= c (10, 71, 6) (10=seedling stage, 71= fruiting stage, 6= maturity stage)

y= c (7, 73, 4) (7=seedling stage, 73= fruiting stage, 4= maturity stage)

z= c (10, 74, 5) (10=seedling stage, 74= fruiting stage, 5= maturity stage)

boxplot (v,w,x,y,z,

names=c("Wire mesh","human guardians","Scarecrow","Thornybush", "open farm"),

col=c("gray","gray","gray","gray","gray"),

xlab="Prevention methods",

ylab="Average number of maize damaged stem")
