# Supplementary material for: Patterns of primates crop foraging and the impacts on incomes of smallholders across the mosaic agricultural landscape of Wolaita zone, southern Ethiopia
Source: PLoS One. 2024 Nov 18;19(11):e0313831. doi: 10.1371/journal.pone.0313831 (PMC11573158; doi:10.1371/journal.pone.0313831)
Supplement: S2 File — (DOCX) [file pone.0313831.s014.docx]

S2 File. The rate of maize damage by grivet monkeys in different crop phenological stages was analyzed in both protected and open/control fields using R code.

Maize damage by grivet monkeys at Kokate Marachare site

v=c (0, 0, 0) (0=seedling stage, 0= fruiting stage, 0= maturity stage)

w= c (0, 6, 1) (0=seedling stage, 6= fruiting stage, 1= maturity stage)

x= c (1, 10, 2) (1=seedling stage, 10= fruiting stage, 2= maturity stage)

y= c (1, 6, 2) (1=seedling stage, 6= fruiting stage, 2= maturity stage)

z= c (1, 7, 1) (1=seedling stage, 7= fruiting stage, 1= maturity stage)

boxplot (v,w,x,y,z,

names=c("Wire mesh","human guardians","Scarecrow","Thornybush", "open farm"),

col=c("gray","gray","gray","gray","gray"),

xlab="Prevention methods",

ylab="Average number of maize damaged stem")
