# Supplementary material for: Patterns of primates crop foraging and the impacts on incomes of smallholders across the mosaic agricultural landscape of Wolaita zone, southern Ethiopia
Source: PLoS One. 2024 Nov 18;19(11):e0313831. doi: 10.1371/journal.pone.0313831 (PMC11573158; doi:10.1371/journal.pone.0313831)
Supplement: S3 File — (PDF) [file pone.0313831.s015.pdf]

MIAMI VALLEY
CAREER TECHNOLOGY CENTER

THIS CERTIFICATE IS AWARDED TO

ABEBAYEHU DESALEGN HAILEMARIAM

in recognition of superior participation, outstanding achievements, and excellent progress in Aspire English for Speakers of Other Languages (ESOL) classes

AUGUST 2023 – MAY 2024

May 23, 2024

Date Issued

Mary Beth Sorlisan

Adult ESOL Instructor
