# Supplementary material for: Patterns of primates crop foraging and the impacts on incomes of smallholders across the mosaic agricultural landscape of Wolaita zone, southern Ethiopia
Source: PLoS One. 2024 Nov 18;19(11):e0313831. doi: 10.1371/journal.pone.0313831 (PMC11573158; doi:10.1371/journal.pone.0313831)
Supplement: S4 File — (PDF) [file pone.0313831.s016.pdf]

Haramaya University
Registrar Office
✉ : 138; ☎: +251255530313

ሐረማያ ዩኒቨርሲቲ
ሬጅስትራር ቢሮ
☎: +251255530331/25

Ref: Ra/03/988/13
Date: 10/06/2013
ቀን: ______

TO WHOM IT MAY CONCERN

Mr/Mrs. Sisay Belay Bedeke has requested us to write him/her a letter verifying the language of instruction at higher learning institutions in Ethiopia. This is, therefore, to kindly inform you that the language of instruction in Ethiopian High School and higher Learning Institutions in general and at Haramaya University in particular, where Mr/Mrs. Sisay Belay Bedeke earned his/her BSc & MSc Degree is English.

Sincerely,

ሐረማያ ዩኒቨርሲቲ
OFFICE OF THE REGISTRAR

In reply, please cite our reference number
