# Supplementary material for: Patterns of primates crop foraging and the impacts on incomes of smallholders across the mosaic agricultural landscape of Wolaita zone, southern Ethiopia
Source: PLoS One. 2024 Nov 18;19(11):e0313831. doi: 10.1371/journal.pone.0313831 (PMC11573158; doi:10.1371/journal.pone.0313831)
Supplement: Attachment — Submitted filename: PONE-D-23-43746__Reviewer_Summary_NSP_Rev_1.pdf [file pone.0313831.s024.pdf]

Tshwane University of Technology

Faculty Science

Department of Nature Conservation

To: Dr. Nickson E. Otieno

Academic Editor PLOS ONE

plosone@plos.org

Submission by link: https://www.editorialmanager.com/pone/l.asp?i=65562495&l=7IGO5R6B

Manuscript ID: PONE-D-23-43746

Title: Crop Damage by Non-human Primates: Quantifying the Keys Parameters of Crop-Raiding events on the Livelihoods of Smallholders in an Agriculture- Forest Mosaic Landscape, Wolaita Zone, Southern Ethiopia

Thank you for affording me the opportunity to provide a review of the manuscript entitled "Crop Damage by Non-human Primates: Quantifying the Key Parameters of Crop-Raiding Events on the Livelihoods of Smallholders in an Agriculture-Forest Mosaic Landscape, Wolaita Zone, Southern Ethiopia." This study explored the conflict between primates, such as baboons (*Papio anubis*) and monkeys (*Chlorocebus aethiops*), and farming practices in southern Ethiopia, effectively demonstrating these animals' significant negative impacts on livelihoods and property. The manuscript was reviewed by reading the manuscript more than once before critiquing, with notable focus on the hypotheses, methodology and supporting statistical evidence (to deduce trustworthy and reproducible science), the synthesis of the crucial findings and conclusions. I therfore offer a review that is systematically and unbiasedly critiqued accompanied by a final read through which was conducted a few days later to look at the material with fresh perpsective. I hope that these qualified opinions and comments will be beneficial with the ultimate aim of ensuring scientific integrity.

While the manuscript is generally well-written, there are a few concerns. The introduction requires structural revisions for improved clarity and coherence. It should begin with a broader discussion of global human-wildlife conflict, particularly focusing on primates' effects on agriculture, to better establish the study's relevance. Additionally, the sections on the Sodo Zuriya and Damot Gale areas need to be integrated more effectively, highlighting their ecological significance and the importance

of these regions for studying human-wildlife conflict. The introduction should also better articulate the problem statement, linking it to existing literature and identifying the research gap this study addresses. Methodologies such as camera traps and community-based studies should be reserved for the methods section. At the same time, the introduction should succinctly outline the study's objectives and emphasize the importance of the findings for conservation and conflict management. Improving the flow and ensuring a logical progression from general to specific information will enhance the introduction's impact. Another concern is the annotation of statistical results and terminology, which needs to be addressed for greater accuracy and clarity.

In conclusion, the study provides valuable insights into primates' foraging behaviour and maize damage across 25 small maize fields, both protected and non-protected. Overall, I was inclined to agree with the idea that this research is crucial for understanding the impacts on maize crops and developing strategies to mitigate wildlife conflict and support the livelihoods of smallholder farmers in the region. Additional comments are detailed in the attached PDF. Should these be addressed, I recommend the manuscript be accepted for publication.

Yours sincerely,

____
Dr Nimmi Seoraj-Pillai, *Pr. Sci. Nat.*

Ph.D., Zoology (WITS); Dip Vocational Teacher Ed., Pedagogical Trainer (Haaga-Helia UoAS, Finland)

Head of Department: Nature Conservation
Tshwane University of Technology, Private Bag X680, Pretoria, 0001, South Africa
Tel: +27 12 382 4669/ 5306| +27 60 626 5454
