# Supplementary material for: Patterns of primates crop foraging and the impacts on incomes of smallholders across the mosaic agricultural landscape of Wolaita zone, southern Ethiopia
Source: PLoS One. 2024 Nov 18;19(11):e0313831. doi: 10.1371/journal.pone.0313831 (PMC11573158; doi:10.1371/journal.pone.0313831)
Supplement: Attachment — Submitted filename: PONE-D-23-43746_reviewer_NSP_Rev 1.pdf [file pone.0313831.s025.pdf]

PLOS ONE

Crop Damage by Nonhuman Primates: Quantifying the Keys Parameters of Crop-Raiding events on the Livelihoods of Smallholders in an Agriculture- Forest Mosaic Landscape, Wolaita Zone, Southern Ethiopia

--Manuscript Draft--

**Manuscript Number:**	PONE-D-23-43746
**Article Type:**	Research Article
**Full Title:**	Crop Damage by Nonhuman Primates: Quantifying the Keys Parameters of Crop-Raiding events on the Livelihoods of Smallholders in an Agriculture- Forest Mosaic Landscape, Wolaita Zone, Southern Ethiopia
**Short Title:**	Crop Damage by Nonhuman Primates
**Corresponding Author:**	Yigrem Kebede Deneke, Msc<br>Wolaita Sodo University<br>Sodo, SODO ETHIOPIA
**Keywords:**	Anubis baboon; Grivet monkeys; Human-Wildlife conflicts; Non-human primate; Maize damage; Prevention method
**Abstract:**	Crop damage caused by non-human primates poses a significant challenge to wildlife conservation efforts. This study aims to assess primates foraging behavior and the extent of maize damage in 25 small (10x10m) maize fields, including both protected and non-protected fields. Data were collected over a twelve-month period spanning 2020 and 2021 in the Sodo Zuriya and Damot Gale regions in the Southern Highlands of Ethiopia. Farmers reported that olive baboons, porcupines, and grivet monkeys were the most notorious crop raiders. Baboons and grivet monkeys were found to attack maize more frequently in June, July, and August. Baboons primarily targeted maize in the morning, while grivet monkeys did so in the afternoon. Notably, primate raids were more common in maize fields located closer to the forest edge than in those situated farther away. The average maize yield losses due to nonhuman primate damage amounted to 43.14% and 31.4% in the protected and non-protected fields, respectively. Within this figure, 43.14% of the damage occurred in the protected fields situated 50 m from the forest edge. Conversely, non-protected fields experienced lower rates of damage: 14.42%, 13.18%, 3.7%, and 0.1% at distances of 50 m, 100 m, 200 m, and 300 m from the forest edge, respectively. Camera traps recorded 47 photos of baboons, 21 photos of grivet monkeys, and documented 8 primate crop foraging events. Consequently, our study concluded that maize fields positioned within 50 meters of the forest edge faced significant primate raids. Despite the utilization of wire mesh fencing, it displayed limited effectiveness in deterring olive baboons and grivet monkeys. Furthermore, while guarding is assumed to be an efficient protective strategy, our findings suggest its ineffectiveness when not implemented continuously.
**Order of Authors:**	Yigrem Kebede Deneke, Msc
	Aberham Megaze, PhD
	Wondimagegehu Tekalegn, PhD
	Taye Dobamo, MSc
	Herwig Leirs, PhD
**Additional Information:**	
**Question**	**Response**
**Financial Disclosure**<br><br>Enter a financial disclosure statement that describes the sources of funding for the work included in this submission. Review the submission guidelines for detailed	Yes

Powered by Editorial Manager® and ProduXion Manager® from Aries Systems Corporation

requirements. View published research articles from *PLOS ONE* for specific examples.<br><br>This statement is required for submission and **will appear in the published article** if the submission is accepted. Please make sure it is accurate.<br><br>**Funded studies**<br>Enter a statement with the following details:<br>• Initials of the authors who received each award<br>• Grant numbers awarded to each author<br>• The full name of each funder<br>• URL of each funder website<br>• Did the sponsors or funders play any role in the study design, data collection and analysis, decision to publish, or preparation of the manuscript?<br><br>Did you receive funding for this work?	
Please add funding details.<br>as follow-up to "**Financial Disclosure**<br><br>Enter a financial disclosure statement that describes the sources of funding for the work included in this submission. Review the submission guidelines for detailed requirements. View published research articles from *PLOS ONE* for specific examples.<br><br>This statement is required for submission and **will appear in the published article** if the submission is accepted. Please make sure it is accurate.	The funders has no role in the study design, data collection and analysis, decision to publish, or preparation of the manuscript

**Funded studies**<br>Enter a statement with the following details:<br>• Initials of the authors who received each award<br>• Grant numbers awarded to each author<br>• The full name of each funder<br>• URL of each funder website<br>• Did the sponsors or funders play any role in the study design, data collection and analysis, decision to publish, or preparation of the manuscript?<br><br>Did you receive funding for this work?"	
Please select the country of your main research funder (please select carefully as in some cases this is used in fee calculation).<br>as follow-up to "**Financial Disclosure**<br><br>Enter a financial disclosure statement that describes the sources of funding for the work included in this submission. Review the submission guidelines for detailed requirements. View published research articles from *PLOS ONE* for specific examples.<br><br>This statement is required for submission and **will appear in the published article** if the submission is accepted. Please make sure it is accurate.<br><br>**Funded studies**<br>Enter a statement with the following details:<br>• Initials of the authors who received each award<br>• Grant numbers awarded to each author<br>• The full name of each funder<br>• URL of each funder website<br>• Did the sponsors or funders play any role in the study design, data collection and analysis, decision to publish, or preparation of the manuscript?<br><br>Did you receive funding for this work?"	BELGIUM - BE
**Competing Interests**	The authors have declared that no competing interests exist.

Use the instructions below to enter a competing interest statement for this submission. On behalf of all authors, disclose any competing interests that could be perceived to bias this work—acknowledging all financial support and any other relevant financial or non-financial competing interests.<br><br>This statement is required for submission and **will appear in the published article** if the submission is accepted. Please make sure it is accurate and that any funding sources listed in your Funding Information later in the submission form are also declared in your Financial Disclosure statement.<br><br>View published research articles from *PLOS ONE* for specific examples.<br><br>**NO authors have competing interests**<br><br>Enter: *The authors have declared that no competing interests exist.*<br><br>**Authors with competing interests**<br><br>Enter competing interest details beginning with this statement:<br><br>*I have read the journal's policy and the authors of this manuscript have the following competing interests: [insert competing interests here]*<br><br>* typeset	
**Ethics Statement**<br><br>Enter an ethics statement for this submission. This statement is required if the study involved:<br><br>• Human participants<br>• Human specimens or tissue<br>• Vertebrate animals or cephalopods<br>• Vertebrate embryos or tissues	The work (VLIR-UOS, ET2019TEA485A102) was funded by the Flemish Interuniversity Council. I obtained written consent from local farmers and acquired a permit letter from the Wolaita Sodo Zone District and Sub-district Agriculture, Environment, Forest, and Climate Change Regulatory Office (Reference no. WZ/253/2013).

Powered by Editorial Manager® and ProduXion Manager® from Aries Systems Corporation

• Field research<br><br>Write "N/A" if the submission does not require an ethics statement.<br><br>General guidance is provided below. Consult the submission guidelines for detailed instructions. **Make sure that all information entered here is included in the Methods section of the manuscript.**	

**Format for specific study types**<br><br>**Human Subject Research (involving human participants and/or tissue)**<br>• Give the name of the institutional review board or ethics committee that approved the study<br>• Include the approval number and/or a statement indicating approval of this research<br>• Indicate the form of consent obtained (written/oral) or the reason that consent was not obtained (e.g. the data were analyzed anonymously)<br><br>**Animal Research (involving vertebrate animals, embryos or tissues)**<br>• Provide the name of the Institutional Animal Care and Use Committee (IACUC) or other relevant ethics board that reviewed the study protocol, and indicate whether they approved this research or granted a formal waiver of ethical approval<br>• Include an approval number if one was obtained<br>• If the study involved *non-human primates*, add *additional details* about animal welfare and steps taken to ameliorate suffering<br>• If anesthesia, euthanasia, or any kind of animal sacrifice is part of the study, include briefly which substances and/or methods were applied<br><br>**Field Research**<br><br>Include the following details if this study involves the collection of plant, animal, or other materials from a natural setting:<br>• Field permit number<br>• Name of the institution or relevant body that granted permission	
**Data Availability**<br><br>Authors are required to make all data underlying the findings described fully available, without restriction, and from the time of publication. PLOS allows rare exceptions to address legal and ethical concerns. See the PLOS Data Policy and FAQ for detailed information.	Yes - all data are fully available without restriction

A Data Availability Statement describing where the data can be found is required at submission. Your answers to this question constitute the Data Availability Statement and **will be published in the article**, if accepted.<br><br>**Important:** Stating 'data available on request from the author' is not sufficient. If your data are only available upon request, select 'No' for the first question and explain your exceptional situation in the text box.<br><br>Do the authors confirm that all data underlying the findings described in their manuscript are fully available without restriction?	
**Describe where the data may be found in full sentences. If you are copying our sample text, replace any instances of XXX with the appropriate details.**<br><br>• If the data are **held or will be held in a public repository**, include URLs, accession numbers or DOIs. If this information will only be available after acceptance, indicate this by ticking the box below. For example: *All XXX files are available from the XXX database (accession number(s) XXX, XXX.).*<br>• If the data are all contained **within the manuscript and/or Supporting Information files**, enter the following: *All relevant data are within the manuscript and its Supporting Information files.*<br>• If neither of these applies but you are able to provide **details of access elsewhere**, with or without limitations, please do so. For example:<br><br>*Data cannot be shared publicly because of [XXX]. Data are available from the XXX Institutional Data Access / Ethics Committee (contact via XXX) for researchers who meet the criteria for access to confidential data.*<br><br>*The data underlying the results presented in the study are available from (include the name of the third party*	All relevant data are within the manuscript and its Supporting Information files.

and contact information or URL).<br>• This text is appropriate if the data are owned by a third party and authors do not have permission to share the data.<br><br>* typeset	
Additional data availability information:	Tick here if the URLs/accession numbers/DOIs will be available only after acceptance of the manuscript for publication so that we can ensure their inclusion before publication.

Powered by Editorial Manager® and ProduXion Manager® from Aries Systems Corporation

Manuscript

Click here to access/download;Manuscript;Primate manuscript.docx

Crop Damage by Nonhuman Primates: Quantifying the Key Parameters of Crop-Raiding Events on the Livelihoods of Smallholders in an Agriculture–Forest Mosaic Landscape, Wolaita Zone, Southern Ethiopia

Yigrem Deneke[1*], Aberham Megaze[1], Wondimagegnheu Tekalign[2], Taye Dobamo[2]and Herwig Leirs[2]

[1]Wolaita Sodo University, College of Natural Sciences, Department of Biology, P.O. box 138, Wolaita Sodo, Ethiopia

[2]University of Antwerp, Department of Biology, Evolutionary Ecology Group, Universiteitsplein 1, 2610 Wilrijk, Belgium

[*] Corresponding author, email: yigremk@gmail.com

Abstract

Crop damage caused by non-human primates poses a significant challenge to wildlife conservation efforts. This study aims to assess primates foraging behavior and the extent of maize damage in 25 small (10x10m) maize fields, including both protected and non-protected fields. Data were collected over a twelve-month period spanning 2020 and 2021 in the Sodo Zuriya and Damot Gale regions in the Southern Highlands of Ethiopia. Farmers reported that olive baboons, porcupines, and grivet monkeys were the most notorious crop raiders. Baboons and grivet monkeys were found to attack maize more frequently in June, July, and August. Baboons primarily targeted maize in the morning, while grivet monkeys did so in the afternoon. Notably, primate raids were more common in maize fields located closer to the forest edge than in those situated farther away. The average maize yield losses due to nonhuman primate damage amounted to 43.14% and 31.4% in the protected and non-protected fiel[illegible] respectively. Within this figure, 43.14% of the damage occurred in the protected fields situated 50 m from the forest edge. Conversely, non-protected fields experienced lower rates of damage: 14.42%, 13.18%, 3.7%, and 0.1% at distances of 50 m, 100 m, 200 m, and 300 m from the forest edge, respectively. Camera traps recorded 47 photos of baboons, 21 photos of grivet monkeys, and documented 8 primate crop foraging events. Consequently, our study concluded that maize fields positioned within 50 meters of the forest edge faced significant primate raids. Despite the utilization of wire mesh fencing, it displayed limited effectiveness in deterring olive baboons and grivet monkeys. Furthermore, while guarding is assumed to be an efficient protective strategy, our findings suggest its ineffectiveness when not implemented continuously.

Key words: Anubis baboon, Grivet monkeys, Human-Wildlife conflicts, Non-human primate, Maize damage, Prevention method

Introduction

Non-human primates that attack subsistence farmers' crops are worrisome since they endanger farmers' livelihoods [1, 2, 3]. Recognizing and managing human-wildlife conflict resulting from crop raiding is a critical conservation issue [4, 5].

The Sodo Zuriya and Damot Gale Community Protected Areas were established in January 2006 through a collaboration between the Sodo community and World Vision Ethiopia. The aim was to restore and protect the montane high-forest on the slopes of Mount Damota in the Southern Ethiopian Highlands. These mountainous landscapes are known for their large populations of endemic animal and plant species, making them an intriguing research area for conservation. The Ethiopian National Biodiversity Institute has identified Mt. Damota in the Wolaita Sodo area of Southern Ethiopia as a priority region for conservation research. According to the institute's assessment, the area also plays a role in global climate regulation [Institute of Biodiversity Conservation, 2005 (6)].

Our study assesses the extent of primate damage to maize crops in human-dominated forest-agricultural mosaic landscapes in Southern Ethiopia. The land is collectively owned by five Sodo Zuriya and Damot Gale Communities, who safeguarded the site and obtained land user-rights certificates from the Ethiopian Government in 2006. Furthermore, the Ethiopian government has supported the community's ownership of carbon rights trading, allowing them to earn revenue from carbon offsets. Cooperatives were established to manage the protected areas. There are reports of several wild herbivorous large mammals in the area damaging maize fields.

Our study combines a camera trap approach with a community-based farmer's participatory study. It is relevant and timely, as the communities around Mt. Damota have begun to protect and coexist with nature and have a good understanding of human-wildlife conflict. Prevention measures are crucial to avoid crop damage by primates on community farms. We evaluate primate crop feeding events and measure the extent of maize damage by primates at various distances from the forest's edge.

Materials and methods

Study area

The study was conducted in the Sodo Zuriya and Damot Gale districts, located approximately at 6.54°N 37.45°E through 6.9°N 37.75°E in the Highlands of Southern Ethiopia. The study sites included the Gurumu Woyde, Kokate Marachere, Konasa Pulasa, Damot Waja, and Dalbo Wogene sub-districts (see Fig. 1). The study area covers 380 km² and is primarily situated atop Mt. Damota. This region experiences a dry period from October to March and a wet season from April to September, receiving 1450 to 1800 mm of rainfall, respectively [7]. The maximum rainfall occurs between June and September, with shorter rains falling in March and April [7]. The temperature ranges from 16°C to 24°C between the wet and dry seasons. The site is characterized by rugged topography and diverse agro-ecology, fauna, and flora. It encompasses both closed forest and open forest areas.

The vegetation is marked by various types, including evergreen needle-leaved, deciduous needle-leaved, evergreen broadleaved, and deciduous broadleaved forests, mixed with shrubland, herbaceous vegetation, herbaceous wetland, moss and lichen, sparse/bare vegetation, and cropland [7]. Dominant plant species in this area include *Syzygium guineense* (woodland waterberry), *Juniperus procera* (African juniper), *Croton macrostachyus* (Broad-Leaved Croton), *Erica arborea* (briar root), *Olea europaea* (common olive), and *Acacia hockii* (Shittim Wood) [7]. The region is home to various large and medium-sized mammals, such as olive baboons (*Papio anubis*), grivet monkeys (*Chlorocebus aethiops*), duikers (*Sylvicapra grimmia*), common bushbucks (*Tragelaphus scriptus*), Guenther's dikdik (*Madoqua guentheri*), and porcupines (*Hystrix cristata*). Predators include golden jackals (*Canis aureus*), black-backed jackals (*Canis mesomelas*), leopards (*Panthera pardus*), African civets (*Civettictis civetta*), and spotted hyenas (*Crocuta crocuta*) [7]. The entire area sustains a population of 16,342 people [8].

The landholding of farmers in Mount Damota is very small. The minimum and maximum sizes of landholding are 0.06 and 1.75 hectares, with an average size of 0.5 hectares [9]. Subsistence farming is the primary source of income for the local population, with crops such as potato (*Solanum tuberosum*), sweet potato (*Ipomoea batatas*), wheat (*Triticum aestivum*), barley (*Hordeum vulgare*), false banana (*Ensete ventricosum*), taro (*Colocasia esculenta*), banana (*Musa acuminata*), maize (*Zea mays*), and common beans (*Phaseolus vulgaris*) [7]. Maize fields

in these areas are very small (e.g.10x10m) and are connected with fields with different crops. Maize fields were selected for this study to assess the extent of damage caused by non-human primates.

Experimental setup

We set up our study using 25 fields. Ten fields were situated 50 meters from the forest edge and were used to compare protective measures in the villages of Gurumu Woide and Kokate Marachare. The protected study plots were safeguarded using wire mesh, human guardians, scarecrows, and thorny bushes, while the non-protected fields remained open/control. Furthermore, we set up a total of fifteen non-protected maize study plots (~~see~~ Table 1), including Gurumu Woide, Kokate Marachare, Delbo Wogene, Damot Waja, and Konasa Pulasa. The study plots were located at varying distances: 100 meters, 200 meters, and 300 meters from the forest edge.

Each study field, we designated a study plot measuring 10m x 10m (~~see~~ Table 1). Within these study plots; we planted the high-yielding maize variety BH-546, well-suited for the region's agro-ecology. Maize seeds were sown early in the rainy season, typically in April, reaching the milky stage in late July and ripening by mid-August, with harvesting in September. Prior to sowing, oxen-drawn plows were used to prepare the fields by creating rows. Initially, 580 seeds were sown in each study plots in both the 2020 and 2021 maize cropping seasons. However, in one field (Field no. 25) seeds were removed or added by the farmer resulting in 532 seeds (19 rows x 28 seeds) during the 2020 maize cropping season and 627 seeds (19 rows x 33 seeds) during the 2021 maize cropping season. Each hole received one seed, with a planting distance of 40 cm x 30 cm, while maintaining a distance of at least 50 meter between one maize study plot and the next.

All cultivation practices, including fertilizer application, cultivation, and weeding, were carried out as usual. However, non-uniform germination of the sown maize seeds resulted in varying maize harvests across different plots.

~~In this study, we collected data using (1) Farmer observation and reports (2) Camera traps~~

Farmer observation and reports

Data collection was carried out by farmers who had received training from researchers. These trained farmers possessed a clear understanding of the nature of Crop Foraging or Raiding Events (CFE/CRE) in primates. All trained farmers monitored and assessed the CFE/CRE of baboons and grivet monkeys. Each farm was involved in this project for two maize harvest seasons (April to August 2020 and 2021 years). The project compensated the participating farmers with monthly payments ranging from 9.5 to 28.5 USD. At the end of each maize growing season, the project paid 19 USD to each of the 25 farmers for their participation in both years (2020 and 2021).

Researchers defined the primate crop foraging or crop raid event (CFE/CRE) to potential aspiring farmers as follows:

CFE /CRE is defined as when one or more individuals of a species entered (i.e. crossed a field boundary) and make trampling the field and left the field (CRE), and interacted with one or more maize stem and eat the stem (CFE). The CFE/CRE episode begins when the first primate enters the field; eat the stem and ends when the last primate leaves the farm. The duration is measured in seconds using a digital stopwatch. Primate age categories are adult (full species-sex-specific size), sub-adult (not fully grown, beyond infant development, exhibits independent behaviour frequently), or infant (developmentally small and dependent, carried frequently, maintains close proximity to adults).

Farmers responded to the following questions: (1) What is the extent of primate damage to maize on protected and non-protected fields? (2) When and during which months do primates raid maize crops? (3) How long do primates typically stay during their maize raids? (4) How frequently and at what times do farmers report primate incursions? (5) Which crop-feeding species have farmers reported encountering? (6) What is the extent of primate maize damage on fields located at a distance? (7) How many individual primates raided maize and entered fields? (8) In what proportion do multiple and single primate raid events occur? (9) How many individual primates typically visit maize fields? (10) In which age categories are maize crop-raiding primates typically found?

Data were also collected regarding the presence or absence of humans on fields, the nature of on-field human activity, the extent of guarding behavior, and responses to crop-raiding primates.

Crop damage was determined by counting stems damaged by primates. Trained farmers assessed and counted the damage caused by primates to maize daily at 18:00 hours. All data collection adhered to institutional ethics requirements, with Reference No. WSU15/12/915 establishing ethical guidelines for social and primate research. This work was conducted with the consent and support of zone administration, village councils, and participating farmers.

Camera traps

To gather information on the timing, frequency, and location of feeding behavior by olive baboons and grivet monkeys within the 25 study plots, we utilized Bushnell camera detection equipment (Browning trail camera Model No BTC-6HDX).

These motion-trigger cameras were configured to capture and store data, including the date, time, location, and temperature for each ~~photo~~. The cameras were set to take only one photo per trigger, with a 2-second interval between triggers [11]. Cameras were securely housed and locked in metal cases. A potential CFE/CRE was recorded when one or more individuals olive baboons and grivet monkeys were merely present in the field [11]. An actual CFE was documented if the photo or video indicated physical manipulation and/or consumption of crop items [11, 12]. An interval of more than an hour between captured images was considered as an independent CFE [11]. During the course of this project, different camera traps were installed and dismantled on different days, resulting in varying numbers of trap days for each unit.

Cameras were installed on each study plot. We used 30mm x 30mm stainless steel wire mesh with a wire diameter of 1.6 mm and a height of 2.5 meters. For data storage, we utilized 16GB and 32GB Class 4 SDHC memory cards for each camera. Farmers monitored the camera traps to prevent theft. Data from the camera traps were collected from April to September in both 2020 and 2021, with cameras installed in each of the 25 maize fields for four consecutive trapping nights. We installed the cameras for 192 trapping days. During camera installation, we collected the following information: camera ID, GPS position, date, and altitude. Subsequently, we downloaded the photos and videos from the camera traps onto a laptop. We checked each photo/video for the presence of wildlife and other relevant information. We also investigated the presence of humans and dogs, among other factors. Photos containing baboons and monkeys that

could damage the crop were numbered and placed in a digital folder. We cataloged all the saved photos/videos and associated information in a spreadsheet.

Data Analysis

We analyzed the data using SPSS Version 16 for Windows (SPSS Inc., Chicago, USA). The images captured by the camera traps were interpreted to determine the frequency and timing of Crop Foraging/CRE Events (CFE/CRE). Descriptive statistics were used for the analysis of crop feeding data. A chi-square test was used to test the variation in the amount of maize damage by primates in fields located at a distance. Primate CFE/CRE analysis involved the use of the Mann-Whitney U test, Spearman's Rank Correlation Coefficient, and the F-test. For the analysis of primate assaults on maize within both preventive and non-preventive maize fields at different seasons and crop phenology, we utilized R-Software [13]. The maize damage was reported in three aspects: the average number of maize stems/cobs affected, the estimated amount of maize damaged in kilograms, and the proportion of maize damage caused by primates in relation to the expected harvest. To calculate the monetary loss, we converted the market prices for maize crop per kilogram to US dollars using the prevailing exchange rate at the time of the survey. Additionally, we estimated that the maize seeds in one maize stalk weighed approximately 0.2 kg after harvest.

Result

Farmers-reported crop feeding or raiding species

All farmers consistently reported that baboons, porcupines, and grivet monkeys were the primary culprits responsible for the most severe crop damage to maize, and these species exhibited a high frequency of Crop Foraging/CRE Events (CFE/CRE). Additionally, some farmers (~~N~~ = 10) suggested that bushbuck might also be involved in crop feeding or raiding. However, the reported CFE/CRE frequency of bushbuck in crop fields was notably low, occurring only 24 times (~~as detailed in~~ Table 2).

Farmer-reported maize damage assessments

The average percentage of maize cobs lost by olive baboons in wire mesh, human guard, scarecrow, and thorny bush setups was 8.23% (equivalent to 72.8 maize stems/cobs), 7.38% (65.3 maize stems/cobs), 9.82% (86.8 maize stems/cobs), and 9.45% (83.5 maize stems/cobs), respectively. These fields were located 50 meters from the forest edge. In non-protected fields, the average percentage of maize cobs lost by olive baboons were 10.04% (88.8 maize stems/cobs), 1.53% (13.5 maize stems/cobs), 0.4% (3.6 maize stems/cobs), and 0.1% (0.9 maize stems/cobs) located at 50 meters, 100 meters, 200 meters, and 300 meters from the forest edge, respectively (~~refer to~~ Table 3).

The average percentage of maize cobs lost by grivet monkeys in wire mesh, human guard, scarecrow, and thorny bush setups were 0, 1.83% (6.3 maize stems/cobs), 3.8% (13 maize stems/cobs), and 2.63% (9 maize stems/cobs), respectively. These fields were located 50 meters from the forest edge. In non-protected fields, the average percentage of maize cobs lost by grivet monkeys were 4.38% (15 maize stems/cobs), 11.65% (39.9 maize stems/cobs), 3.3% (11.3 maize stems/cobs), and 0, located at 50 meters, 100 meters, 200 meters, and 300 meters from the forest edge, respectively (refer to Table 3).

In total, the average percentage of maize cobs lost by these two primate species in the protected fields was 43.14% (equivalent to 336.7 maize stems), located at 50 meters from the forest edge. The average percentage of maize cobs lost by primates in the non-protected fields was 14.42%

(103.8 maize stems), 13.18% (53.4 maize stems), 3.7% (14.9 maize stems), and 0.1% (0.9 maize stems) located at 50 meters, 100 meters, 200 meters, and 300 meters, respectively.

Camera trap results

Our cameras recorded 47 photographs of baboons and 21 photographs of grivet monkeys, as summarized in Table 3. Of the 47 photographs of baboons, only ~~3~~ were confirmed as actual (CFE), while the remaining 44 were potential (CRE). Similarly, out of the 21 photographs of grivet monkeys, only ~~2~~ were confirmed as actual (CFE), with the remaining 19 being potential (CRE). Notably, the longest CRE event, recorded by camera ID A3 and E1, occurred in scarecrow and open maize fields (see Table 4, Figure 3).

Farmers-reported extent of primate crop damage on protected and open/control fields

The average percentage of maize damaged by Olive baboons in both Gurumu Woide and Kokate Marachare study sites, as reported by farmers, was 23.62% in wire mesh, 21.03% with a human guard, 28.15% with a scarecrow, and 27.2% in thorny bush fields, respectively (~~as illustrated in~~ Fig. 4).

The results ~~of a one-way ANOVA~~ indicated that the amount of damage in maize fields was significantly higher in thorny bush fields compared to the levels of damage from wire mesh, a human guard, and a scarecrow (F=292.5, df=11, p < .001, see Figure 4).

The average percentage of maize damaged by grivet monkeys in the Kokate Marachare study site, as reported by farmers, was 0% in wire mesh, 24.14% with a human guard, 44.83% with a scarecrow, and 31.03% in thorny bush fields, respectively (~~as illustrated in~~ Fig. 5).

~~The results of a one-way ANOVA~~ indicated that the amount of damage in maize fields was significantly higher in thorny bush fields compared to the levels of damage from wire mesh, human guards, and scarecrows (F=5.4, df=11, p < .005, see Figure 5).

Time or months of maize raided

According to farmers' responses, a higher frequency of maize cobs being plucked was reported in July, with 524 ± 3.8 maize cobs in the year 2020 and 539 ± 4.6 maize cobs in the year 2021. Moderate frequencies of maize raiding were reported in June and August, with 216 ± 4.6 and 64

± 2.1 maize cobs in 2020, and 240 ± 5.2 and 25 ± 1.6 maize cobs in 2021, respectively. The lowest frequencies of maize raiding occurred in April, May, and September for both 2020 and 2021 (as illustrated in Fig 6).

Duration of crop-raiding events

The average raid duration ranged from 15.1 to 18 minutes, with a standard deviation of 0.66. There was significant difference in raid duration between species, as indicated by the Kruskal-Wallis test ($\chi^2 = 58.62$, d.f. = 10, $P < 0.05$).

Raid durations were significantly shorter when carried out by single individuals (~~median 1 minute, SD = 0.42~~) compared to raids by two or more individuals (median 3 minutes, SD = 2.42), as confirmed by the Mann-Whitney U test (~~n (single) = n(two+) = 38,~~ U = 34.0, $p < 0.001$). The majority of Crop Raiding Events (CREs), approximately 70%, lasted between 0.1 and 12 minutes (see Fig 7)

Farmers-reported CFE frequency and timing

Farmers observed that baboons typically fed on crops early in the morning, while grivet monkeys fed on crops throughout the day. According to farmers, neither baboons nor grivet monkeys were seen eating on crops at night. Baboon Crop Foraging Events (CFEs) occurred throughout the day but not in a uniform distribution, as revealed by photographic data from five locations (Chi-square ~~goodness of fit~~: $\chi^2 = 32.36$, df = 12, $p < 0.001$). Similarly, Grivet monkey CFEs occurred throughout the day, also with a non-uniform distribution, based on photographic data from five locations (~~Chi-square goodness of fit;~~ $\chi^2 = 35.86$, df = 8, $p < 0.001$). Morning CFEs were more common in baboons (6:00–7:00 a.m.) than afternoon CFEs (2:00–3:30 p.m.). In contrast, CFEs were more common in the early afternoon (11:00 a.m.–12:00 p.m.) for grivet monkeys than in the morning (6:00–7:00 a.m.) during both 2020 and 2021 years. Farmers reported no baboon CFEs in all five locations between 11:00 a.m. and 6:00 p.m. during both 2020 and 2021 years (~~as depicted in~~ Fig. 8).

Primate crop raiding events

A total of 367 primates were observed at the forest edges immediately before or during Crop Raiding Events (CREs). Out of these, 367 individuals, accounting for 75%, ventured into fields (~~refer to~~ Table 5). This included all 75 CREs by Anubis baboons (79%) and 20 CREs by grivet monkeys (21%). Notably, Anubis baboons were significantly more likely to be found near the forest edge than grivet monkeys~~, as indicated by the Kruskal-Wallis test~~ ($\chi^2 = 263.1$, df = 15, $p < 0.001$). The number of individuals entering a field showed a positive correlation with the number at the forest edge prior to raiding, which was confirmed by the Spearman's Rank Correlation Coefficient (~~rs~~ = 0.434, n = 95, p = 0.006). This correlation persisted even when humans were present on the field, with a Spearman's Rank Correlation Coefficient of ~~rs~~ = 0.324, n = 59, and p = 0.04. Regarding the composition of CREs, the majority (36.1%) involved three or fewer individuals, while 47.8% consisted of a single individual or a pair. Only 16.1% of CREs involved more than five individuals (~~as illustrated in~~ Fig. 9). It's worth noting that baboons raided in significantly larger groups than other species~~, as shown by the Kruskal-Wallis test~~ ($\chi^2 = 41.57$, df = 5, $p < 0.001$); however, most baboon raiding groups were small, with 78% comprising fewer than five individuals. On the other hand, grivet monkeys were more likely to raid alone~~, according to the Kruskal-Wallis test~~ ($\chi^2 = 88.01$, df = 5, $p < 0.001$).

Multiple versus Single raid events

A significantly greater proportion of raids (64%; n = 61) occurred in groups rather than as single raids~~, as confirmed by the Chi-square test~~ ($\chi^2 = 15.9$, df = 4, p = 0.003). Among the group raids, 67% consisted of either 2-CRE or 3-CRE groupings, indicating a diverse pattern of multiple-CRE profiles for both grivet monkeys and baboons (~~as depicted in~~ Fig 10). On the other hand, single raids accounted for 36% (n = 34) and were more likely to involve a single raiding individual. It's worth noting that the extent of maize crop damage per CRE differed significantly between single raids and group raids~~, as evidenced by the F-test~~ (F = 22.17, df = 1, $p < 0.001$).

Primate field visit and crop raiding events

Seventy-five percent of primate field visits (comprising 22.3% baboons and 26.1% grivets) did not involve crop raiding ~~at all, as illustrated in~~ Fig 12. Among the visits that did include crop raiding, it was observed that 76% more baboon visits involved multiple crop-raiding events

rather than a single event. In the case of grivets, 53% more visits involved multiple events~~, as confirmed by the Chi-square test~~ (baboon ~~-~~ χ^2_1 = 11.63, df = 1, p < 0.001; Grivet ~~-~~ χ^2_1 = 16.00, df = 1, p < 0.001; ~~as depicted in~~ Fig 11 and ~~Fig~~ 12).

Age categories composition of crop-raiding primates

Significantly more adults were observed on study plots during CREs compared to sub-adults, and more sub-adults were observed than infants. These differences were statistically significant (Mann-Whitney U tests: n (sub-adult) = 118, n (adult) = 216, U = 1653.5, p < 0.001; n (infant) = 33, n (sub-adult) = 118, U = 952.0, p = 0.510). This age category distribution was consistent for each primate species~~, as confirmed by a Chi-square test~~ (χ^2 = 71.4, df = 1, p < 0.001) (~~refer to~~ Table 6). Nearly 58% (n = 55) of raiders were single adults, and the majority of adults were present in 42% of CREs involving multiple individuals (n = 40). Baboons exhibited mixed age-category raiding groups significantly more frequently than grivet monkeys (~~Kruskal-Wallis test,~~ χ^2 = 58.05, df = 5, p < 0.001), and baboon raiding groups were more diverse (~~Kruskal-Wallis test,~~ χ^2 = 10.88, df = 4, p = 0.028). At least one infant was observed during six baboon raids. Most baboon and grivet raiders were accompanied by an adult during their raids. Almost two-thirds of baboon raiding groups included one or more sub-adults. All on-field adult and sub-adult primates damaged at least one crop stem. While infants occasionally interacted with crops by pulling or biting stems, they often traveled or rested near an adult female or engaged in play behavior with other infants or sub-adults, suggesting they were not anxious during CREs. Female primates with infants were particularly vigilant on fields; they were usually the first to return to the forest while carrying their infants and the first to flee in response to human actions. The sex of raiding individuals was not reliably determined for analysis; however, counts of male (n = 38) and female (n = 14) adult baboons on-field during CREs did not significantly differ (~~Chi-square test,~~ χ^2 = 29.45, df = 1, p < 0.001). While significantly more maize stems were damaged by mixed-age groups than by adults-only groups, the former groups also comprised more individuals, traveled further onto fields, and raided for longer durations~~. These findings were supported by~~ Mann-Whitney U tests (n (adults) = 10.0, n(mixed) = 36: stems U = 2840.5, p = 0.021; individuals U = 20.5, p = 0.367; maximum distance U = 24.5, p = 1.000; median distance U = 429.0, p = 1.000; duration U = 528.5, p < 0.001).

Discussion

Numerous primate species have been involved in crop raids, as documented in various studies [14, 15, 16, 17, 18, 19]. In this study, the average maize yield losses attributed to nonhuman primate damage were estimated at 43.14% and 31.4% in the protected and non-protected fields, respectively, equivalent to 1704.4 maize stems/cobs (340.8 kg) per hectare. Among these losses, 43.14% occurred in protected fields located 50 m from the forest edge. In contrast, non-protected fields experienced lower damage percentages: 14.42%, 13.18%, 3.7%, and 0.1% at distances of 50 m, 100 m, 200 m, and 300 m from the forest edge, respectively. The resulting monetary losses for farmer households amounted to 15,864 ETB (equivalent to 444 US Dollars) from an expected income of 21,600 ETB (equivalent to 608 US Dollars) per hectare.

The intensity of crop raids by Anubis baboons varied across different villages, and with no baboon attacks documented in some of the areas. Baboons, like many other primates [20], do not uniformly utilize all parts of their home ranges [21, 22, 23]. Their area use patterns are influenced by factors such as food distribution, sleeping sites or refuge availability, water access, and the presence of predators [21, 23, 24]. This observation aligns with findings in reference [25], suggesting that primate crop-foraging decisions are influenced by crop nutritional quality, spatial and temporal crop availability in comparison to wild food resources. The phenomenon of maize raiding appeared impervious to variations in forest fruit abundance [26], but it may be affected by interspecific interactions, such as predator-prey relationships, which can impact primate foraging behaviors [27].

A study by [30] noted an average maize yield loss of 264.1 kg per hectare due to pests (baboons and pigs), representing 34.2% of the anticipated total yield. In the Budungo Forest Reserves of Uganda, a study by [2] reported that farmers observed 73% of crop damage caused by primates. Similarly, in another study [40] conducted in the Taita Hills of Kenya, characterized by a forest-agricultural mosaic landscape, farmers reported that 87% of the maize crop was attacked by primates. Our study demonstrates the value of strategically positioned camera traps in providing insights into various aspects, including recording primate species, their targeted crop types and growth phases, daily and seasonal patterns of crop-feeding activity, and whether crop-feeding occurs individually or in groups [11]. However, it's important to acknowledge the limitations of this method. Although camera traps allow the identification of large, solitary animals with

distinct markings [31, 11], we encountered challenges in identifying active crop feeders, particularly in terms of age and sex classification. Our identifications were likely biased toward more conspicuous individuals, primarily adult males [11]. Additionally, while camera traps may capture evidence of primate groups' presence in fields, they may not consistently provide photographic evidence of actual crop manipulation and consumption, as supported by a reference [11]. Therefore, many events identified as Crop Foraging Events (CFEs) through camera traps may not indeed be actual CFEs. To assess the severity of crop damage caused by primate feeding, we supplemented our research with additional methods, including farmers' reports. These reports helped monitor baboon and grivet monkey behavior, estimate daily maize damage, and assess post-harvest damage, as supported by references [32, 33]. Thus, both farmers' reports and camera trap data offered valuable information by specifying the crops targeted by primate species and identifying the most frequent and destructive crop-feeding species, such as baboons, grivet monkeys, porcupines, and bushbucks.

Both Anubis baboons and grivet monkeys showed a tendency to cause more damage to maize fields closer to the forest edge compared to those farther away. The proportion of fields raided by pests was significantly higher in villages near forests compared to those situated away from the forests [30, 52]. Primates predominantly raided crops within 10 meters of the farm-forest edges [17, 34, 35]. The primate raiding groups is influenced by factors like body size, human proximity from the forest [14].

The behavior of primates in the study area was influenced by their habits and foraging, with baboons on rocky cliffs and caves and grivet monkeys in large trees within the forest. Based on our field observation, Gurumu Woide has high forest fruit availability and an abundance of steep cliffs and caves suitable for the existence of a baboon troop. In contrast, Kokate, Konasa, Delbo, and Waja have lower forest fruit availability and fewer steep cliffs and caves for baboon survival. This may explain the reduced maize damage by baboons in these study sites. However, it's worth noting that the specific magnitude of forest fruit availability and the number of cliff and caves in these study villages remains unknown. In this study, the distance traveled by both baboons and grivet monkeys to inspect and raid crops did not vary, as both species traveled up to 300 meters. During our observations in the caves, we found that baboons were located at far distances, approximately 400 meters from the first farmer fields. Consequently, baboons raided

the crops that are available close to the forest edge. However, baboons still visited farms located 300 meters from the forest edge, even though maize crop feeding events were infrequent at this distance. When crops were not available, both species turned to forest foods, foraging on fruits, leaves, flowers, bark, and roots of forest plant species. In Uganda, grivet monkeys ventured up to 55 meters into crop fields, while baboons reached up to 110 meters [38]. The highest distance observed was over 700 meters, notably in the Ngangao Forest in Taita Hills, Kenya [40]. This variation may be influenced by the distribution of households and the number of farms investigated at different distances [40].

In this study, maize raids by primates were reported during the maturation of maize cobs. Our findings suggest that scarecrows and thorn bushes were generally ineffective in deterring the return of baboons or grivet monkeys to the fields. Our wire mesh (wire diameter of 1.6 mm and a mesh size of 30 mm x 30 mm, and a height of 2.5 meters) protection method reduced maize damage, but it did not deter baboons from raiding the crop, and they quickly habituated. Kokate Marachare is one of our experimental study sites; in this site, the wire mesh fence was effective in discouraging olive baboons and grivet monkeys from attacking maize crops in fields located 50 meters from the forest's edge, but it was not effective in discouraging olive baboons in the Gurumu Woide. We hypothesize that the presence of multiple baboons in the Gurumu Woide study site made them highly vigilant and determined to raid maize crops despite the crop fenced with wire mesh fences. In contrast, in the Kokate Marachare, where only a single baboon was involved, hence the wire mesh fence most likely deterred them from crop raiding. According to a ~~paper~~ by [29], the net wire fences exhibited limited effectiveness against primate raiding in Budongo Forest Reserve, Uganda. Guarding proved ineffective in preventing baboon attacks on maize fields in Gurumu Woide due to the absence of continuous field guards. Indeed, field guards were often absent due to other (social) activities, school attendance etc. However, a study conducted by [30], where it was found that continuous guarding is a principal strategy for effectively mitigating crop damage by pests. The extended protection duration was particularly necessary in villages at higher altitudes where maize takes longer to mature [30].

Both baboons and grivet monkeys are frequently observed foraging for crops in human-dominated settings in the study area, with baboons causing more damage than grivet monkeys. The time of day had differing effects on the crop-foraging patterns of the two species, with

baboons foraging more frequently in the morning and grivet monkeys in the afternoon. This variation in the time of activity might be related to the presence of baboons, which appeared to deter grivet crop-foraging behavior [28]. Similarly, the time activity pattern varied in different areas; reference [41] recorded a peak in baboon crop foraging in Zimbabwe between 8 and 10 am, potentially driven by the need to find food upon waking. In contrast, a reference [39] found that primates in Uganda foraged on crops more frequently between noon and sunset than between sunrise and noon. To access crops, baboons were observed using a 'sit and wait' strategy near the edge of crop fields [42]. The more time baboons and grivet monkeys spent close to the fields, the more likely they were to forage within crops. Furthermore, when they entered crops during these visits, they were more likely to enter multiple times. Crop raiding wasn't a behavior practiced by all members of primate social groups, with baboon raiding parties averaging five individuals [17].

In our study, more adults were observed on maize fields during CREs compared to sub-adults. This varies in different areas; in some studies, adult primates were the main crop raiders, as referenced in [17, 18, 34, 44], while in other studies, sub-adults were identified as the primary raiders, as cited in [45, 46, 47, 48]. However, this behavior was rare and observed only in baboons. Additionally, perceptions of risk may impact the age composition of primate raiding groups, with adult females with infants raiding the least frequently, likely due to increased caution, as suggested by [19, 51]. However, the diverse raiding group compositions among baboons, the presence of infants on fields, and high rates of raiding by baboons suggested that they were generally more comfortable on fields than other primate species. Although the age-category composition of raiding groups did influence crop loss, it was a secondary effect related to group size. The extensive profiles of multiple ~~Crop Raiding Events (~~CREs~~)~~ for grivet monkeys and baboons indicated that these species persistently raid crops when opportunities arise. The Effective deterrent techniques for farmers should aim to discourage raiding by multiple individuals, to reduce raiding group sizes, or minimize the time primates spend on fields. However, it's essential to acknowledge that primates with a substantial history of raiding can habituate quickly to deterrent techniques. In general, assessing the parameters of ~~Crop Raiding Events (~~CREs~~)~~ offers accurate indicators of how comfortable primates are on fields and it provides essential information for managing and mitigating human-wildlife conflicts.

Conclusion

The findings from this study underscore the pressing issue of human-wildlife conflict, particularly in areas where forests and agriculture intersect. Our combined approach, utilizing farmers' reports and camera traps to assess the extent of crop damage and Crop Foraging Events (CFEs) caused by primate pests, highlights the substantial crop losses observed in our study area. These losses emphasize the need for continuous vigilance over maize fields, from sowing to harvest, to deter wild mammal pests. The investment of time, effort, and resources in this endeavor is justified by the magnitude of crop protection required. Wire mesh fencing was found to exhibit limited effectiveness in deterring baboons and grivet monkeys. Although guarding is assumed to be an efficient protective strategy, our study revealed its ineffectiveness when implementation lacks continuity. In a broader context, understanding the spatio-temporal dynamics of wildlife-induced crop deprivation, evaluating parameters related to Crop Foraging Events (CRE), and formulating ecologically--based approach for primate pest management and prevention strategies are crucial steps in mitigating the socio-economic impacts of wild primate pests originating from forest edges.

Acknowledgments

We express our gratitude to the Environment Protection, Forest and Climate Change Regulatory Office of Wolaita Zone, Ethiopia, for granting us permission to conduct this research. We extend our sincere appreciation to Tadiwos T/Mariam, Hailemariam Lota, Alana Asale, Metalo Bentha, Tariku Milkano, and Mengistu Shirko for their invaluable field support. We are also thankful to the University of Antwerp, Belgium, and Wolaita Sodo Universities, Ethiopia, for their support. Our special thanks go to the farmers, families, and village/sub-district chairpersons of Sodo Zuriya and Damot Gale for their unwavering support, enthusiasm, and hospitality throughout our research.

Funding

We sincerely thank VLIR-UOS (Flemish Interuniversities Council) for financial support

Data Availability Statement

The data supporting this study is available in the article

Author Contributions

Conceptualization: Yigrem Deneke,

Data curation: Yigrem Deneke

Formal analysis: Yigrem Deneke

Investigation:Yigrem Deneke, Aberham Megaze,Taye Dobamo, Wondimagegnheu Tekalign

Methodology: Yigrem Deneke

Project administration: Herwig Leirs

Resources: Herwig Leirs

Supervision: Herwig Leirs, Aberham Megaze

Writing – original draft: Yigrem Deneke

Writing – review & editing: Yigrem Deneke, Herwig Leirs, Aberham Megaze

References

1. Hill CM. Conflict of interest between people and baboons: Crop raiding in Uganda. Inter J Prima. 2000; 21: 299–315.
2. Tweheyo M, Hill CM, Obua J. Patterns of crop raiding by primates around the Budongo Forest Reserve, Uganda. Wildl Biol. 2005; 11: 237-247.
3. Campbell-Smith G, Simanjorang HVP, Leader-Williams N, Linkie M. Local attitudes and perceptions toward crop-raiding by orangutans (*Pongo abelii*) and other nonhuman primates in northern Sumatra, Indonesia. Ameri J Primat. 2010; 72:866–876.
4. Sitati NW, Walpole MJ. Assessing farm-based measures for mitigating human-elephant conflict in Transmara District, Kenya. Oryx. 2006; 40: 279–286.
5. Graham MD, Ochieng T. Uptake and performance of farm-based measures for reducing crop raiding by elephants (*Loxodonta Africana*) among smallholder farms in Laikipia District, Kenya. Oryx. 2008; 42: 76–82.
6. Institute of biodiversity conservation (2005). National biodiversity strategy and action plan. Addis Ababa, Ethiopia.

7. World Vision Ethiopia. The Sodo Community Managed Reforestation (Forest Regeneration) Project "Many Species, one Planet, and one Future"). Submission to the Climate, Community and Biodiversity Standard. Sported by World Vision Australia. 2010; 64p.
8. CSA. Federal Democratic Republic of Ethiopia Central Statistical Agency. Population and Housing Census. 2015; Addis Ababa, Ethiopia.
9. Merkineh MM. Determinants of Choice Decision for Adoption of Conservation Intervention Practices: The Case of Mt. Damota Sub-Watershed, Wolaita Zone, Ethiopia. *Global Journal of HUMAN-SOCIAL SCIENCE: B Geography, Geo-Sciences, Environmental Science & Disaster Management*. Global Journals Inc. (USA). 2016, Volume 16, ISSN: 0975-587X.
10. Satellite images. Land use/cover data. 2017, 2018, and 2019; downloaded from USGS/Earth Explorer website. https://earthexplorer.usgs.gov
11. Zak AA, Riley EP. Comparing the Use of Camera Traps and Farmer Reports to Study Crop Feeding Behaviour of Moor Macaques *(Macaca maura).* Int. J. Prima. 2016*;* 38(2): 224–242. https://doi.org/10.1007/s10764-016-9945-6.
12. Acrenaz M, Hearn A, Ross J, Sollmann R, Wilting A. Handbook for wildlife monitoring using camera-traps. 2012; Kota Kinabalu, Sabah, Malaysia: BBEC II Secretariat
13. R Core Team. R: A language and environment for statistical computing. R Foundation for Statistical Computing, Vienna, Austria. 2020; https://www.R-project.org/
14. Wallace, GE, Hill, CM. Crop Damage by Primates: Quantifying the Key Parameters of Crop-Raiding Events. PLoS one. 2012; 7(10): e46636. Doi: 10.1371/ journal.pone.0046636. PMID: 23056378
15. Maples WR, Maples MK, Greenhood WF, Walek ML. Adaptations of crop-raiding baboons in Kenya. Ameri J Physi Anthropo. 1976; 45: 309–315.
16. Crockett CM, Wilson WL. The ecological separation of *Macaca nemestrina* and *M. fascicularis* in Sumatra. In: Lindburg DG, editor. The macaques: Studies in ecology, behavior and evolution. 1980; New York: Van Nostrand Reinhold. 148– 181 p.
17. Warren Y. Olive baboons (*Papio cynocephalus anubis*): Behaviour, ecology and human conflict in Gashaka Gumti National Park, Nigeria [PhD Thesis]. 2003; Roehampton: University of Surrey. 308 p.
18. Priston NEC. Crop-raiding by *Macaca ochreata brunnescens* in Sulawesi: Reality, perceptions and outcomes for conservation [PhD Thesis]. 2005; Cambridge: University of Cambridge.

19. Hockings KJ. Human-chimpanzee coexistence at Bossou, the Republic of Guinea: A chimpanzee perspective [PhD Thesis]. 2007; Stirling: University of Stirling.

20. Willems EP., Barton RA, Hill RA. Remotely sensed productivity, regional home range selection, and local range use by an omnivorous primate. Behav Ecol, 2009; 20: 985–992

21. Cowlishaw G. Trade-offs between foraging and predation risk determine habitat use in a desert baboon population. Anim Behav, 1997b; 53: 667–686.

22. Pebsworth PA, Macintosh AJJ, Morgan HR, Huffman MA. Factors influencing the ranging behavior of chacma baboons (Papio hamadryas ursinus) living in a human-modified habitat. Intern J of Primat, 2012; 33: 872–887.

23. Whiten A, Byrne RW, Henzi SP. The behavioural ecology of mountain baboons. Intern J of Primat, 1987; 8: 367–388.

24. Cowlishaw G. Refuge use and predation risk in a desert baboon population. Anim Behav, 1997a; 54:241–253.

25. Hill CH. Crop-raiding. Intern Encyclo of Primat. 2017; 10.1002/9781119179313, p1-5. DOI: 10.1002/9781119179313.wbprim0109.

26. Naughton-Treves L, Treves A, Chapman C, Wrangham, R. Temporal Patterns of Crop-Raiding by Primates: Linking Food Availability in Croplands and Adjacent Forest. J Appl Ecol. 1998; 35 (4): 596-606

27. Willems EP, Hill RA. Predator-specific landscapes of fear and resource distribution: effects on spatial range use. J Ecol. 2009; 90:546–555.

28. Findlay, LJ, Hill, RA. Baboon and vervet monkey crop-foraging behaviours on a commercial South African farm: preliminary implications for damage mitigation. Hum Wildl Intera. 2020*;* 14(3):505–518

29. Sara SH, Caroline R ,Catherine MH and Wallace GE. crop-raiding deterrents around budongo forest reserve: an evaluation through farmer actions and perceptions. *Oryx*, 2013; 47: 569 - 577 DOI: https://doi.org/10.1017/S0030605312000853

30. Ango TG, Borjeson L, Senbeta F. Crop raiding by wild mammals in Ethiopia: impacts on the livelihoods of smallholders in an agriculture–forest mosaic landscape. Oryx, 2016;51(3):527–

537 Fauna & Flora International doi: 10.1017/S0030605316000028.

31. Wegge P, Chiranjibi PP, Jnawali SR. Effects of trapping effort and trap shyness on estimates of tiger abundance from camera trap studies. Anim Conserv, 2004; 7: 251–256

32. Hill CM. Crop-raiding by wild vertebrates: The farmer's perspective in an agricultural community in western Uganda. Inter J Pest Manage. 1997; 43: 77–84.

33. Linkie M, Dinata Y, Nofrianto A. Leader Williams N. Patterns and perceptions of wildlife crop raiding in and around Kerinci Seblat National Park, Sumatra. Anim Conserv, 2007; 10:127–135. https://doi.org/10.1111/j.1469-1795.2006.00083.x

34. Hansen LK. Influence of forest-farm boundaries and human activity on raiding by the Buton macaque (*Macaca ochreata brunnescens*) [MSc Dissertation]. 2003; Oxford: Oxford Brookes University. 46 p.

35. Priston NEC, Wyper RM, Lee PC. Buton macaques (*Macaca ochreata brunnescens*): Crops, conflict, and behavior on farms. Ameri J Primat. 2012; 74: 29–36.

36. Wolfheim JH. Primates of the world: Distribution, abundance, and conservation. Seattle. 1983; University of Washington Press. 831 p.

37. Nowak RM. Walker's primates of the world. Baltimore. 1999; The Johns Hopkins University Press. 224 p.

38. Reynolds V. The chimpanzees of the Budongo Forest: Ecology, behaviour, and conservation. 2005; Oxford: Oxford University Press. 297 p.

39. Wallace GE. Monkeys in maize: primate crop-raiding behaviour and developing on-farm techniques to mitigate human–wildlife conflict. Dissertation, 2010; Oxford Brookes University, Oxford, United Kingdom.

40. Siljander Mika, Kuronen Toini, Johansson T, Munyao MN, Pellikka Petri KE. Primates on the farm – spatial patterns of human–wildlife conflict in forest-agricultural landscape mosaic inTaitaHills,Kenya.ApplGeogra.2020;117:102185.https://doi.org/10.1016/j.apgeog.2020.102185

41. Schweitzer C, Gaillard T, Guerbois C, Fritz H, O. Petit O. Participant profiling and pattern of crop-foraging in chacma baboons (*Papio hamadryas ursinus*) in Zimbabwe: why does investigating age–sex classes matter? Inter J Prima. 2017; 38: 207–223.

42. Walton BJ, Findlay LJ, Hill RA. Insights into short- and long-term crop-foraging strategies in a chacma baboon (*Papio ursinus*) from GPS and accelerometer data. Ecol Evol. 2021; 11:990–1001. https://doi.org/10.1002/ece3.7114

43. Webber AD. Primate crop raiding in Uganda: Actual and perceived risks around Budongo Forest Reserve [PhD Thesis]. 2006; Oxford: Oxford Brookes University.

44. Hockings KJ. Living at the interface: Human-chimpanzee competition, coexistence and conflict in Africa. Inter Studi. 2009; 10: 183–205.

45. Strum SC. Prospects for management of primate pests. Revue d'Ecologie (La Terre Et La Vie). 1994; 49: 295–306.

46. Forthman QDL. Activity budgets and the consumption of human food in two troops of baboons, Papio anubis, at Gilgil, Kenya. In: Else JG, Lee PC, editors. Prima ecol conserv. 1986; Cambridge: Cambridge University Press. 221–228.

47. Oyaro HO, Strum SC. Shifts in foraging strategies as a response to the presence of agriculture in a troop of wild baboons at Gilgil, Kenya. Inter J Prima. 1984; 5: 371–381.

48. Saj T, Sicotte P, Paterson JD. Influence of human food consumption on the time budget of vervets. Inter J Prima. 1999; 20: 977–994.

49. Fairbanks LA. Risk-taking by juvenile vervet monkeys. Behav. 1993; 124: 57– 72.

50. Janson CH, van Schaik CP. Ecological risk aversion in juvenile primates: Slow and steady wins the race. In: Pereira ME, Fairbanks LA, editors. Juvenile primates: Life history, development and behavior. 1993; New York: Oxford University Press. 57–74.

51. Fairbanks LA, McGuire MT. Maternal protectiveness and response to the unfamiliar in vervet monkeys. Ameri J Prima. 1993; 30: 119–129.

52. Alemayehu Mamo, Debissa Lemessa, Obsu Hirko Diriba,Debela Hunde. Pattern of crop raiding by wild large mammals and the resultant impacts vary with distances from forests in Southwest Ethiopia. Ecol Evol. 2021; 11:3203–3209. DOI: 10.1002/ece3.7268. PMID: 33841777

Fig 1. Background vegetation map based on remote sensing data [10]
Fig 2. Various prevention strategies (Wire mesh (A), Human guardian tower (B), Scarecrow (C), Thorny bush (D)) were assessed in eight experimental maize field sites to evaluate their effectiveness in deterring crop raiders. The study was conducted in maize field sites located in Gurumu Woide and Kokate Marachare.

Fig 3. The images above depict camera trap captures of various wildlife species observed in maize field sites located in Damota Mountain, Southern Ethiopia: (A) Anubis baboons (*Papio anubis*) (B) Grivet monkeys (*Chlorocebus aethiops*), (C) Porcupine (*Hystrix cristata*), and (D) Bushbuck (*Tragelaphus scriptus*)

Fig 4. The average of maize stems (≈number of cobs) damaged within 10m x 10m study plots by Olive baboons was examined in relation to various preventive methods at a distance of 50 meters from the forest edge during the 2020 and 2021 maize cropping seasons and crop phenology in the Gurumu Woide and Kokate Marachare (GW) sub-district. The boxplot illustrates a significant difference in crop damage among different prevention methods ($p < .001$).

Fig 5. The average of maize stems (≈the number of cobs) damaged within 10m x 10m study plots by grivet monkeys illustrates the relationship with various prevention methods at a distance of 50 meters from the forest edge during the 2020 and 2021 maize cropping seasons and crop phenology in the Kokate Marachare (KM) sub-district. The boxplot shows a significant difference in crop damage with different prevention methods ($p < .005$).

Fig 6. The frequency of primate maize crop raided during the 2020 and 2021 maize cropping seasons ($n = 95$)

Fig 7. Relative frequency of raid durations by primate CREs ($n = 95$).

Fig 8. The frequency of baboon and grivet monkey CFEs by time of day ($N = 95$) between April to September 2020 and 2021 years.

Fig 9. Relative frequency of raiding by primate CREs ($n = 95$)

Fig 10. The frequency distribution of CREs that were single raids or within a series of multiple-CREs for each of these two primate species ($n = 95$)

Fig 11. The number of baboon and grivet monkey field visits that did and did not involve crop-raiding events (CRE) on maize fields in the Highlands of Damota Mountain, April to September 2020 and 2021 years ($n=367$)

Fig 12. The number of baboon and grivet monkey field visits that involved single- and multi-crop raiding events on maize fields in the Highlands of Damota Mountain, April to September 2020 and 2021 years ($n=189$).

Table 1. Maize field and study plot size on the protective and non-protective maize fields

Study sites	Field number	Maize field size in hectare	Study plot size (10x10m)	Distance to forest edge	Preventive and non-preventive measures
Gurumu Woide	1	0.01	0.01	50m	Wire mesh
	2	0.06	0.01	50m	Human guard
	3	0.1	0.01	50m	Scarecrow
	4	0.1	0.01	50m	Thorny bushy
	5	0.1	0.01	50m	Open/control
	6	0.2	0.01	100m	Open
	7	0.3	0.01	200m	Open
	8	0.3	0.01	300m	Open
Kokate Marachare	9	0.2	0.01	50m	Wire mesh
	10	0.2	0.01	50m	Scarecrow
	11	0.2	0.01	50m	Thorny bushy
	12	0.2	0.01	50m	Open/control
	13	0.2	0.01	50m	Human guard
	14	0.3	0.01	100m	Open
	15	0.3	0.01	200m	Open
	16	0.3	0.01	300m	Open
Delbo Wogene	17	0.2	0.01	100m	Open
	18	0.2	0.01	200m	Open
	19	0.3	0.01	300m	Open
Damot Waja	20	0.06	0.01	100m	Open
	21	0.3	0.01	200m	Open
	22	0.3	0.01	300m	Open
Konasa Pulasa	23	0.01	0.01	100m	Open
	24	0.3	0.01	200m	Open
	25	0.3	0.01	300m	Open

Table 2. The comparison of farmer response frequency and CFE/CRE frequency of crop feeding/raiding species from April to September 2020 and 2021 years

Pest species	Number of farmers reporting the species	Frequency of CFE/CRE
Baboon (*Papio anubis*)	22	80
Grivet monkey (*Chlorocebus aethiops)*	17	45
Porcupine (*Hystrix cristata*)	25	75
Common bushbuck (*Tragelaphus scriptus*)	10	24

Table 3. Farmer observation and reported of maize damage assessments (580 maize stem expected per plot except field no. 25 (see the text)

Study sites	Field number	Distance to forest	measures	Olive baboons						Grivet monkeys					
				Maize cobs loss		% damaged		Av. damaged	Av.%	Maize cobs loss		% damaged		Av. damaged	Av. %
				2020	2021	2020	2021	2020/21	2020/21	2020	2021	2020	2021	2020/21	2020/21
Gurumu Woide	1	50m	Wire mesh	145	146	16.57	16.35	145.5	16.46	0	0	0	0	0	0
	2	50m	guard	127	128	14.51	14.33	127.5	14.42	0	0	0	0	0	0
	3	50m	Scarecrow	165	167	18.86	18.7	166	18.78	0	0	0	0	0	0
	4	50m	Thorny	160	161	18.29	18.03	160.5	18.16	0	0	0	0	0	0
	5	50m	Open/control	164	168	18.74	18.81	166	18.78	0	0	0	0	0	0
	6	100m	Open	48	54	5.48	6.05	51	5.77	0	0	0	0	0	0
	7	200m	Open	16	13	1.83	1.46	14.5	1.64	0	0	0	0	0	0
	8	300m	Open	4	5	0.46	0.56	4.5	0.51	0	0	0	0	0	0
Kokate Marachare	9	50m	Wire mesh	0	0	0	0	0	0	0	0	0	0	0	0
	10	50m	guard	4	2	0.46	0.22	3	0.34	12	13	3.56	3.74	12.5	3.65
	11	50m	Scarecrow	7	8	0.8	0.9	7.5	0.85	25	27	7.42	7.76	26	7.59
	12	50m	Thorny	6	7	0.69	0.78	6.5	0.74	17	19	5.04	5.46	18	5.26
	13	50m	Open/control	11	12	1.26	1.34	11.5	1.3	29	31	8.61	8.91	30	8.76
	14	100m	Open	15	18	1.71	2.02	16.5	1.86	11	12	3.26	3.44	11.5	3.36
	15	200m	Open	3	4	0.34	0.45	3.5	0.39	2	5	0.59	1.43	3.5	1.02
	16	300m	Open	0	0	0	0	0	0	0	0	0	0	0	0
Delbo Wogene	17	100m	Open	0	0	0	0	0	0	30	28	8.9	8.04	29	8.47
	18	200m	Open	0	0	0	0	0	0	9	12	2.67	3.44	10.5	3.07
	19	300m	Open	0	0	0	0	0	0	0	0	0	0	0	0
Damot Waja	20	100m	Open	0	0	0	0	0	0	40	42	11.9	12.1	41	11.97
	21	200m	Open	0	0	0	0	0	0	10	10	2.97	2.87	10	2.92
	22	300m	Open	0	0	0	0	0	0	0	0	0	0	0	0
Konasa Pulasa	23	100m	Open	0	0	0	0	0	0	117	119	34.7	34.19	118	34.45
	24	200m	Open	0	0	0	0	0	0	35	30	10.4	8.62	32.5	9.48
	25	300m	Open	0	0	0	0	0	0	0	0	0	0	0	0
Total				**875**	**893**	**100**	**100**	**884**	**100**	**337**	**348**	**100**	**100**	**342.5**	**100**

Table 4. Image data of olive baboon and grivet monkeys by camera traps of twenty five maize fields during 2020 and 2021

Study sites	Camera ID	Distance to forest edge	Preventive and Non-preventive measures	Olive baboon		Grivet monkey	
				CRE	CFE	CRE	CFE
Gurumu Woide	A1	50m	Wire mesh	4	0	0	0
	A2	50m	Human guard	10	0	0	0
	A3	50m	Scarecrow	12	3	0	0
	A4	50m	Thorny bushy	6	0	0	0
	A5	50m	Open/control	9	0	0	0
	A6	100m	Open	3	0	0	0
	A7	200m	Open	0	0	0	0
	A8	300m	Open	0	0	0	0
Kokate Marachare	B1	50m	Wire mesh	0	0	0	0
	B2	50m	Scarecrow	0	0	1	0
	B3	50m	Thorny bush	0	0	1	0
	B4	50m	Open/control	0	0	1	0
	B5	50m	Human guard	0	0	0	0
	B6	100m	Open	0	0	1	0
	B7	200m	Open	0	0	0	0
	B8	300m	Open	0	0	0	0
Delbo Wogene	C1	100m	Open	0	0	1	0
	C2	200m	Open	0	0	0	0
	C3	300m	Open	0	0	0	0
Damot Waja	D1	100m	Open	0	0	1	0
	D2	200m	Open	0	0	0	0
	D3	300m	Open	0	0	0	0
Konasa Pulasa	E1	100m	Open	0	0	11	2
	E2	200m	Open	0	0	2	0
	E3	300m	Open	0	0	0	0
**Total**				**44**	**3**	**19**	**2**

Table 5. The proportion of the total number of on-field primates during CREs (n = 367) that were adults, sub-adults, or infants.

	Total number of individuals on fields			
	Adults	Sub-adults	Infants	Total
Anubis baboon	151 (57.6%)	78 (29.8%)	33 (12.6%)	262
Grivet monkey	65 (61.9%)	40 (38.1%)	0 (0%)	105
Total	216	118	33	367

Table 6. Age-category composition of primate raiding groups during CREs (n = 95).

	Composition of crop-raiding group			
	Adults only	Adults and sub-adults	Adults and infants	Adults, sub-adults, infants
Species	% CREs	% CREs	% CREs	% CREs
Anubis baboon	36	45	4.4	14.6
Grivet monkey	68	32	0.0	0.0

Figure

Click here to access/download;Figure;Fig 1.docx

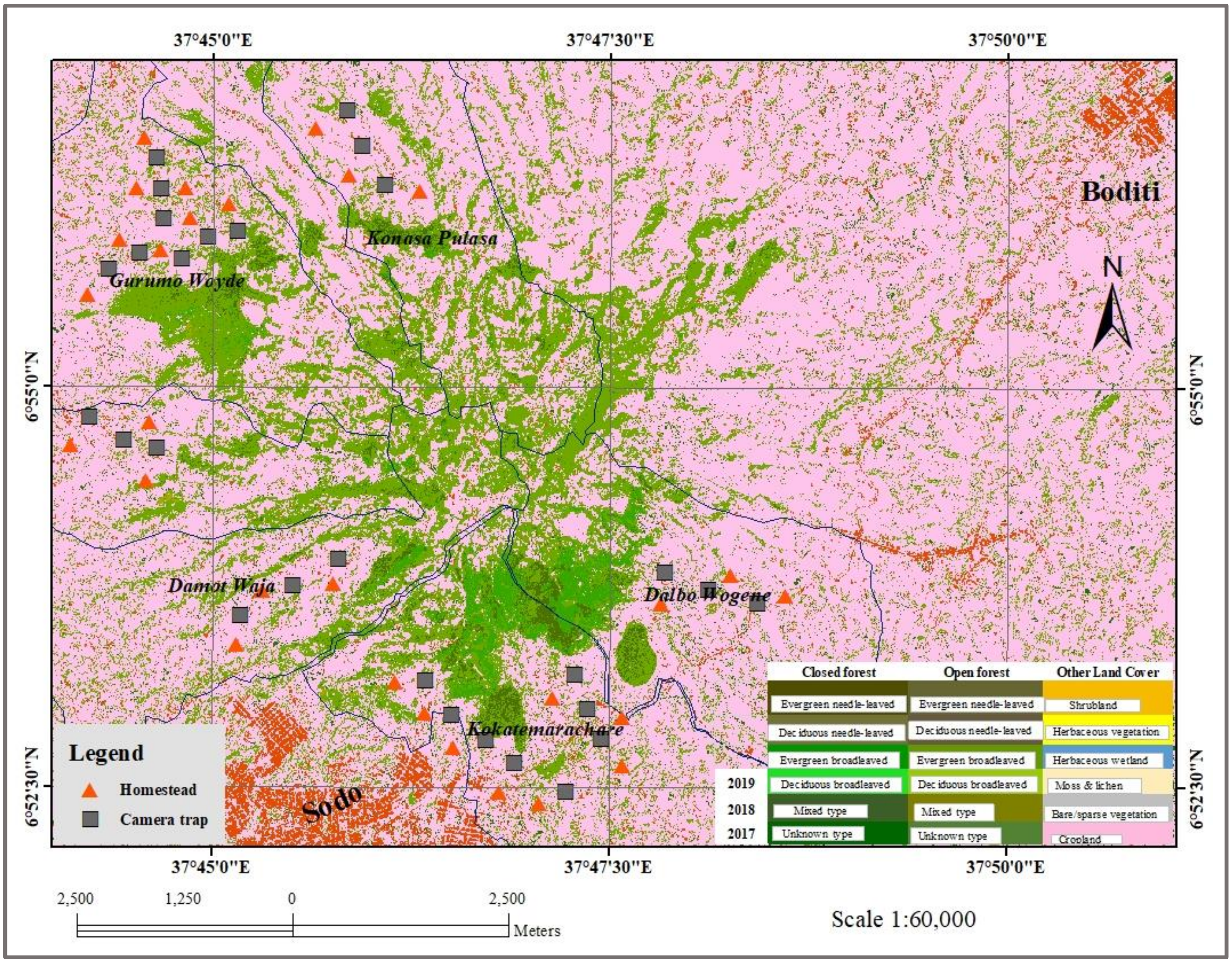

Fig 1. Background vegetation map based on remote sensing data [10]

Figure
Click here to access/download;Figure;Fig 2.docx

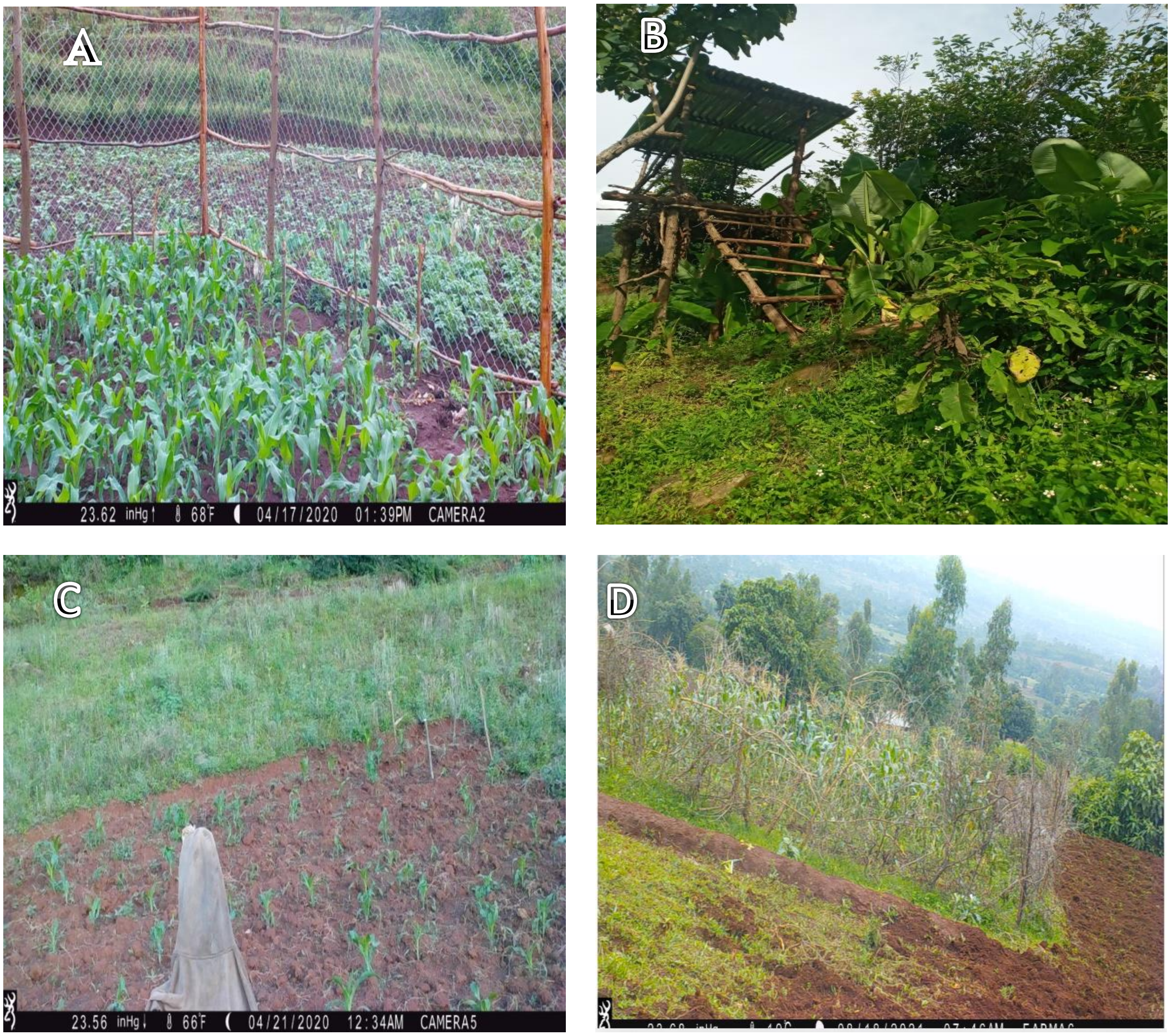

Fig 2. Various prevention strategies (Wire mesh (A), Human guardian tower (B), Scarecrow (C), Thorny bush (D)) were assessed in eight experimental maize field sites to evaluate their effectiveness in deterring crop raiders. The study was conducted in maize field sites located in Gurumu Woide and Kokate Marachare.

Figure Click here to access/download;Figure;Fig 3.docx

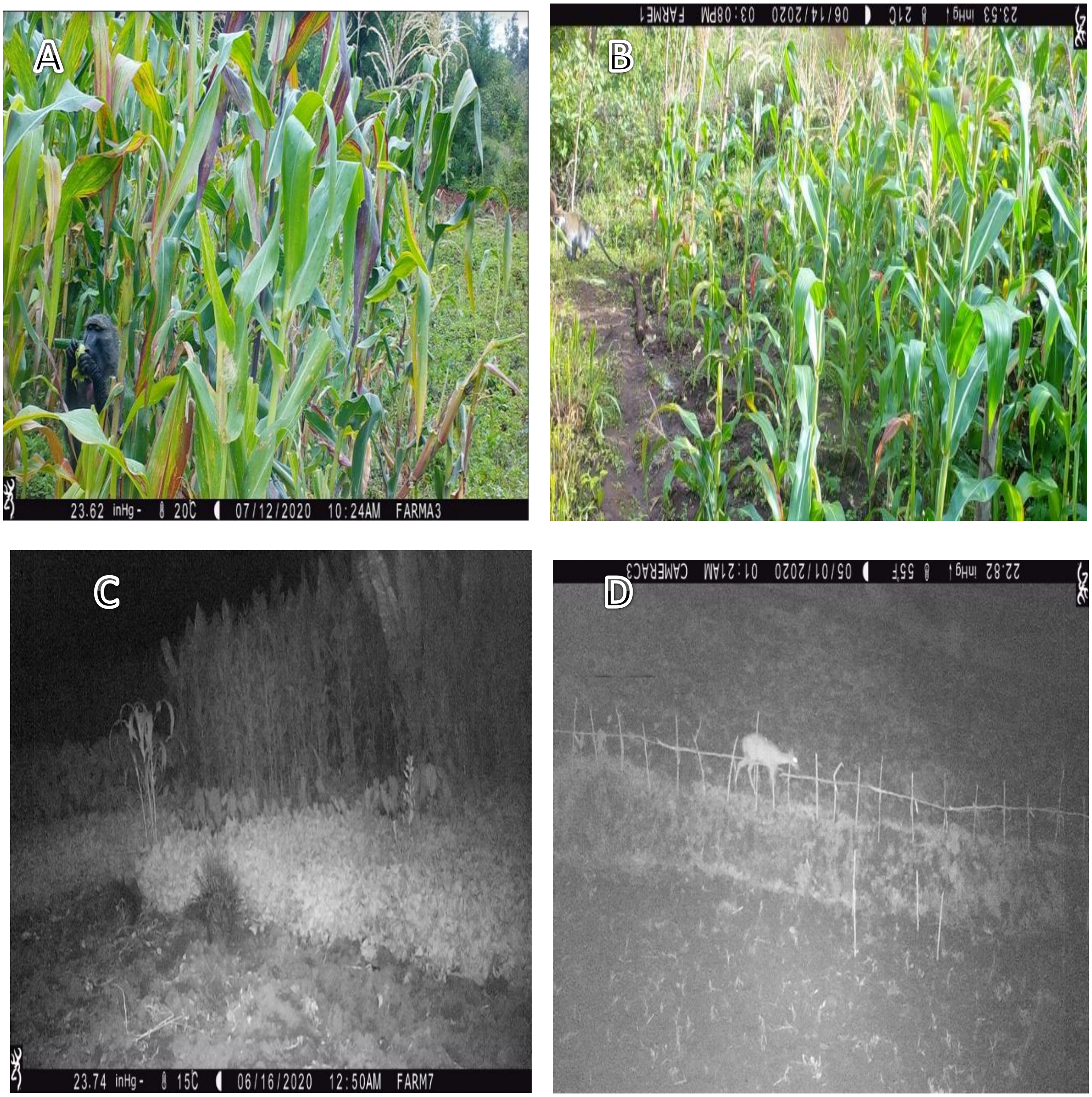

Fig 3. The images above depict camera trap captures of various wildlife species observed in maize field sites located in Damota Mountain, Southern Ethiopia: (A) Anubis baboons (*Papio anubis*) (B) Grivet monkeys (*Chlorocebus aethiops*), (C) Porcupine (*Hystrix cristata*), and (D) Bushbuck (*Tragelaphus scriptus*)

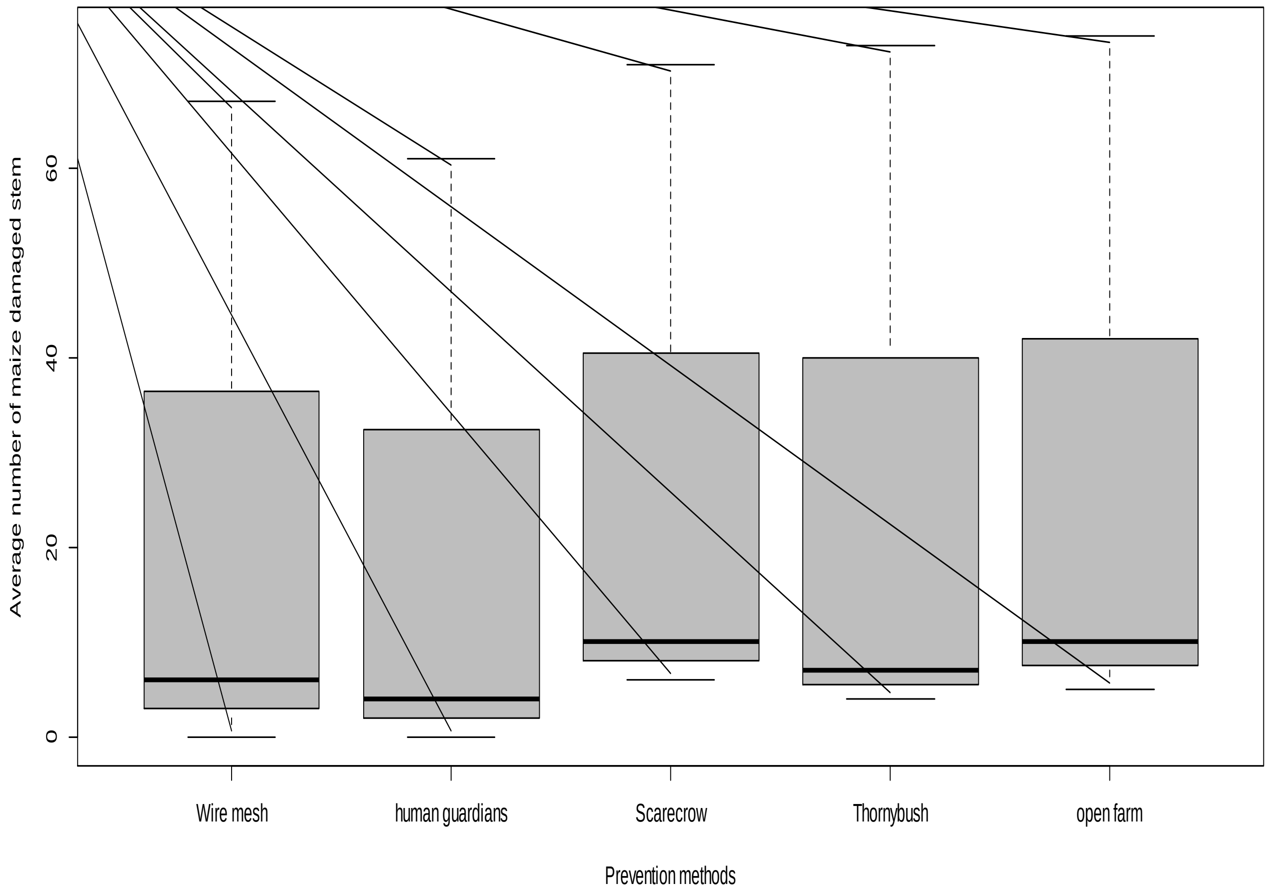

Fig 4. The average of maize stems (≈number of cobs) damaged within 10m x 10m study plots by Olive baboons was examined in relation to various prevention methods at a distance of 50 meters from the forest edge during the 2020 and 2021 maize cropping seasons and crop phenology in the Gurumu Woide and Kokate Marachare (GW) sub-district. The boxplot illustrates a significant difference in crop damage among different prevention methods ($p < .001$).

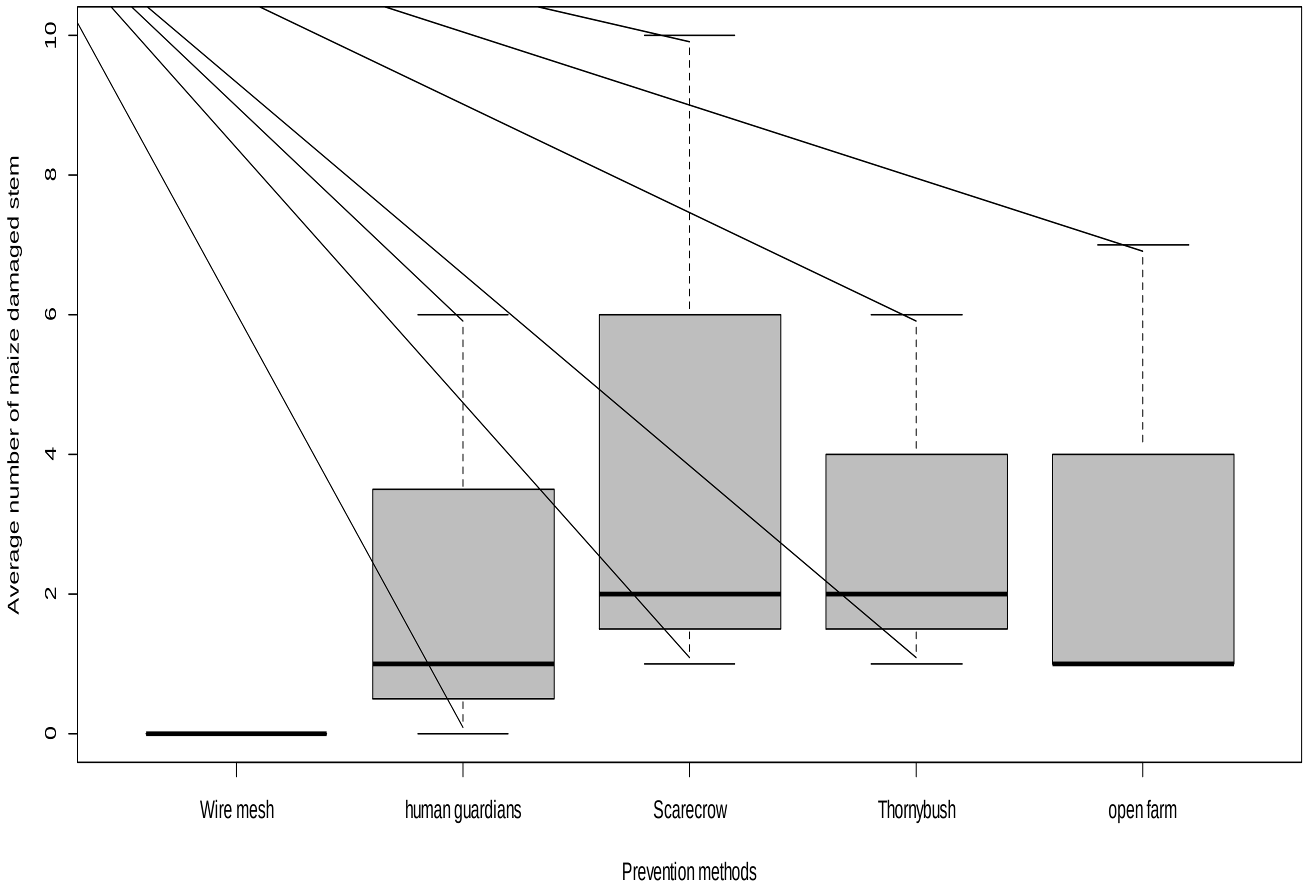

Fig 5. The average of maize stems (≈the number of cobs) damaged within 10m x 10m study plots by grivet monkeys illustrates the relationship with various prevention methods at a distance of 50 meters from the forest edge during the 2020 and 2021 maize cropping seasons and crop phenology in the Kokate Marachare (KM) sub-district. The boxplot shows a significant difference in crop damage with different prevention methods ($p < .005$).

Figure Click here to access/download;Figure;Fig 6.docx

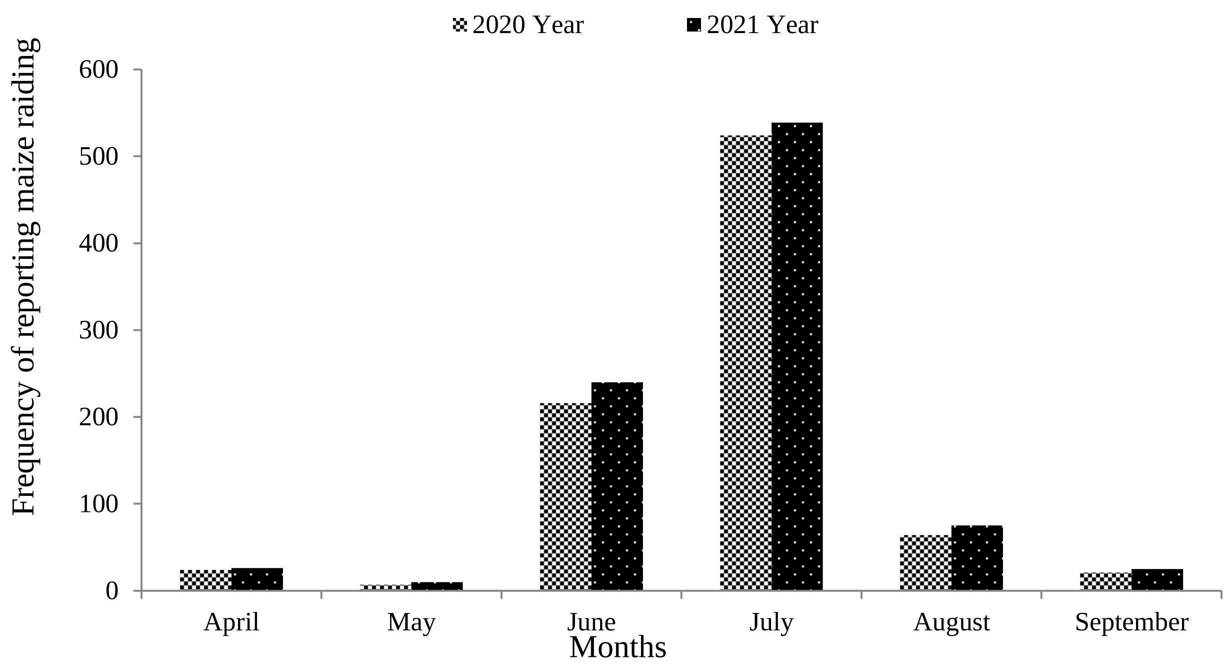

Fig 6. The frequency of primate maize crop raided during the 2020 and 2021 maize cropping seasons (n = 95)

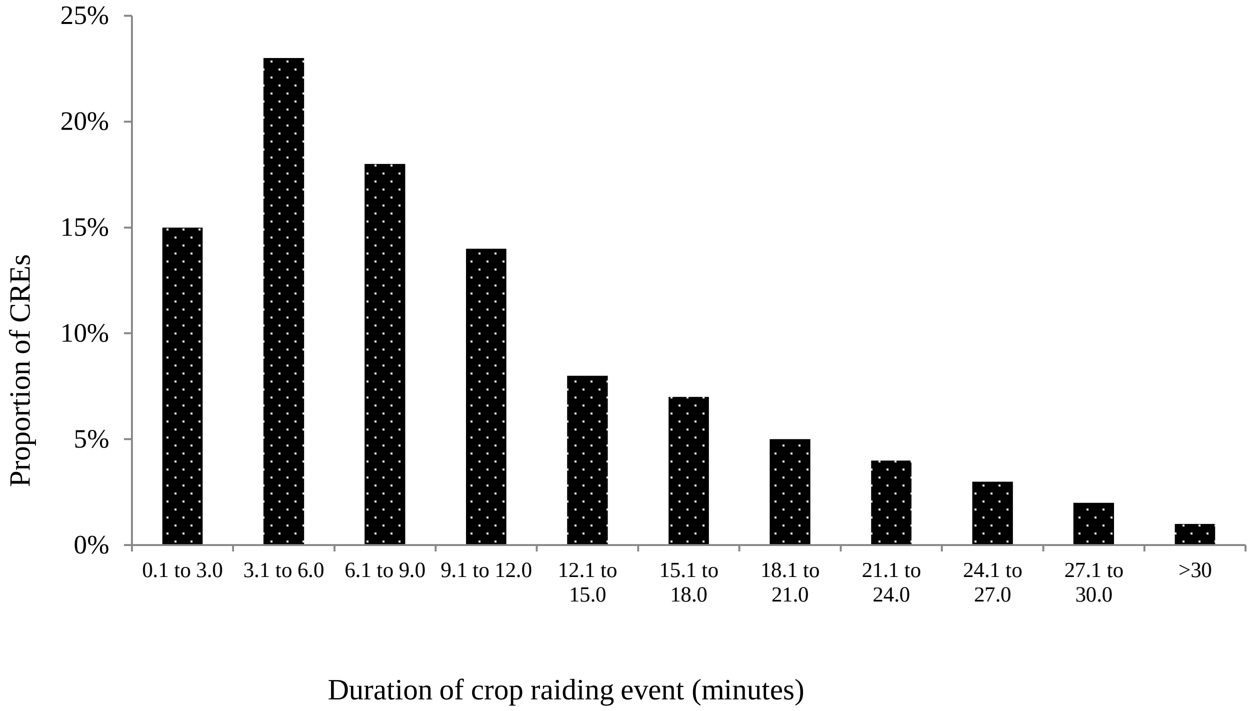

Fig 7. Relative frequency of raid durations by primate CREs (n = 95).

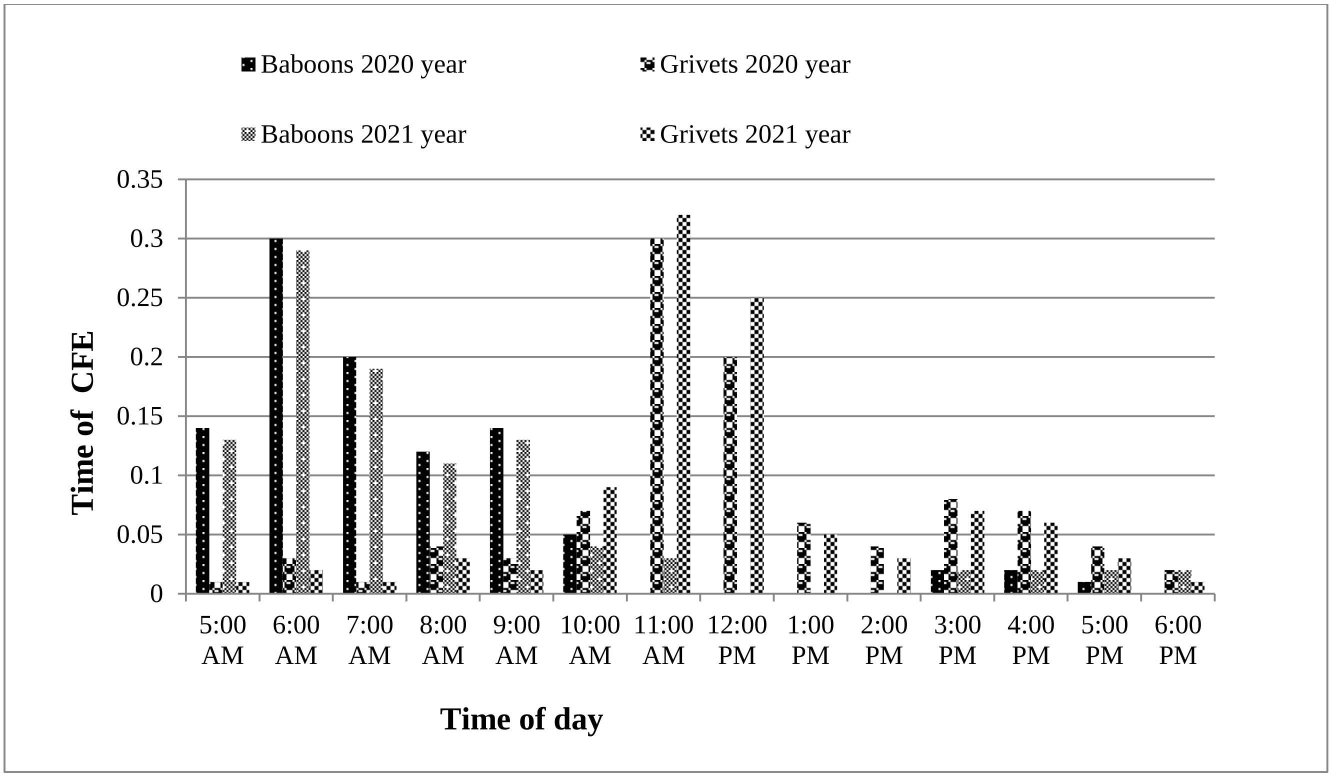

Fig 8. The frequency of baboon and grivet monkey CFEs by time of day (N = 95) between April to September 2020 and 2021 years.

Click here to access/download;Figure;Fig 9.docx

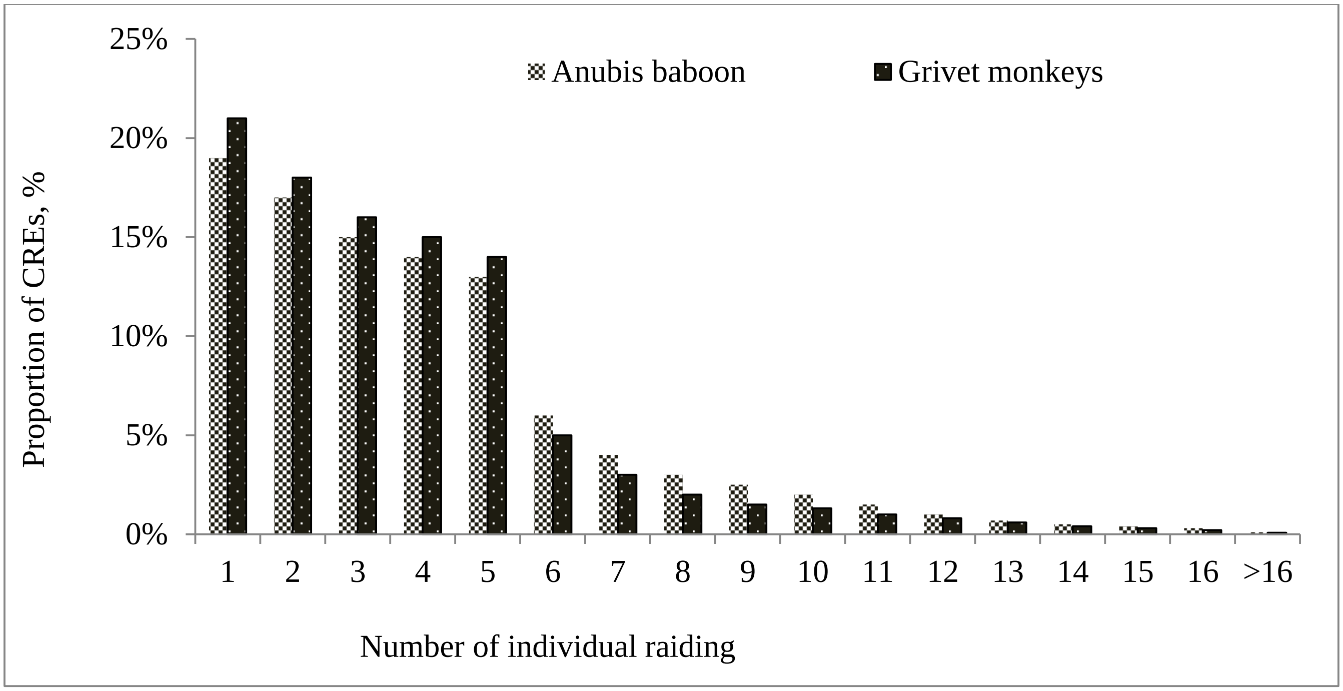

Fig 9. Relative frequency of raiding by primate CREs (n = 95)

 Click here to access/download;Figure;Fig 10..docx

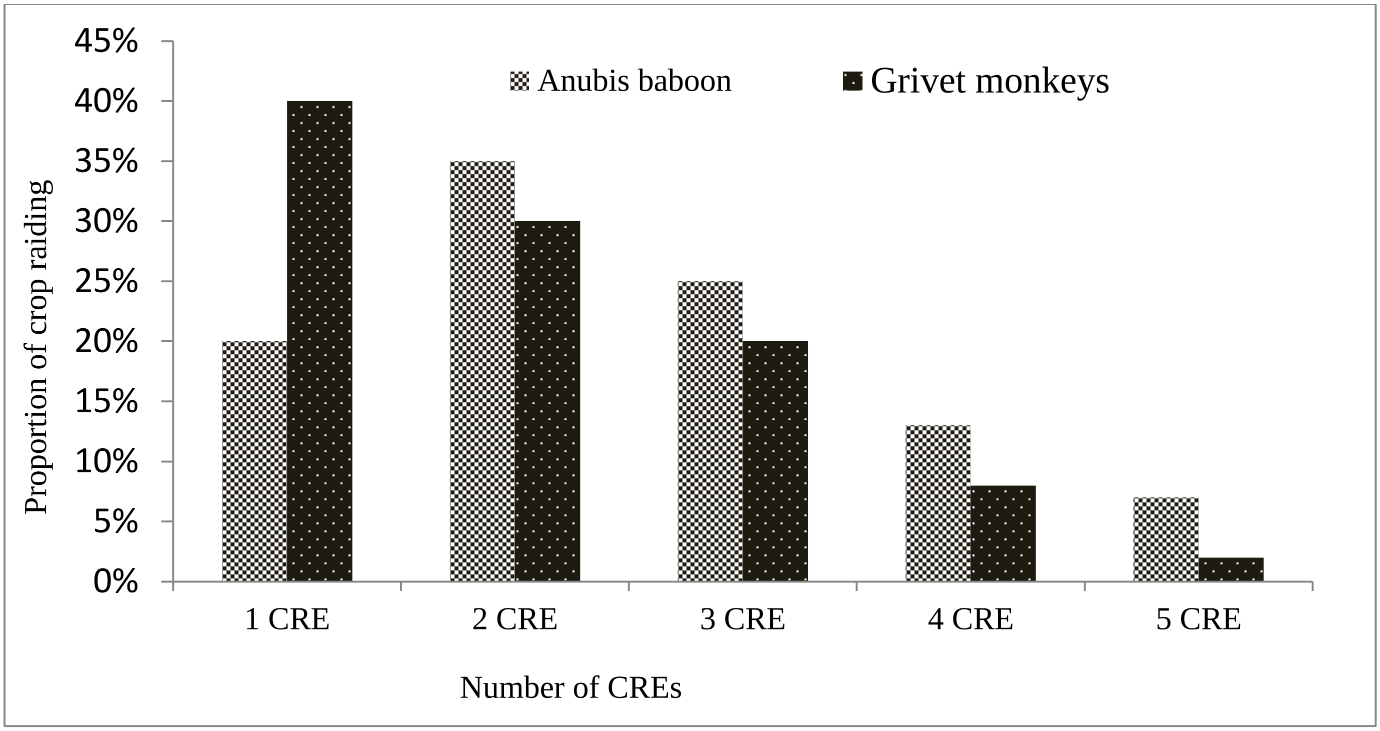

Fig 10. The frequency distribution of CREs that were single raids or within a series of multiple-CREs for each of these two primate species (n = 95)

 Click here to access/download;Figure;Fig 11.docx

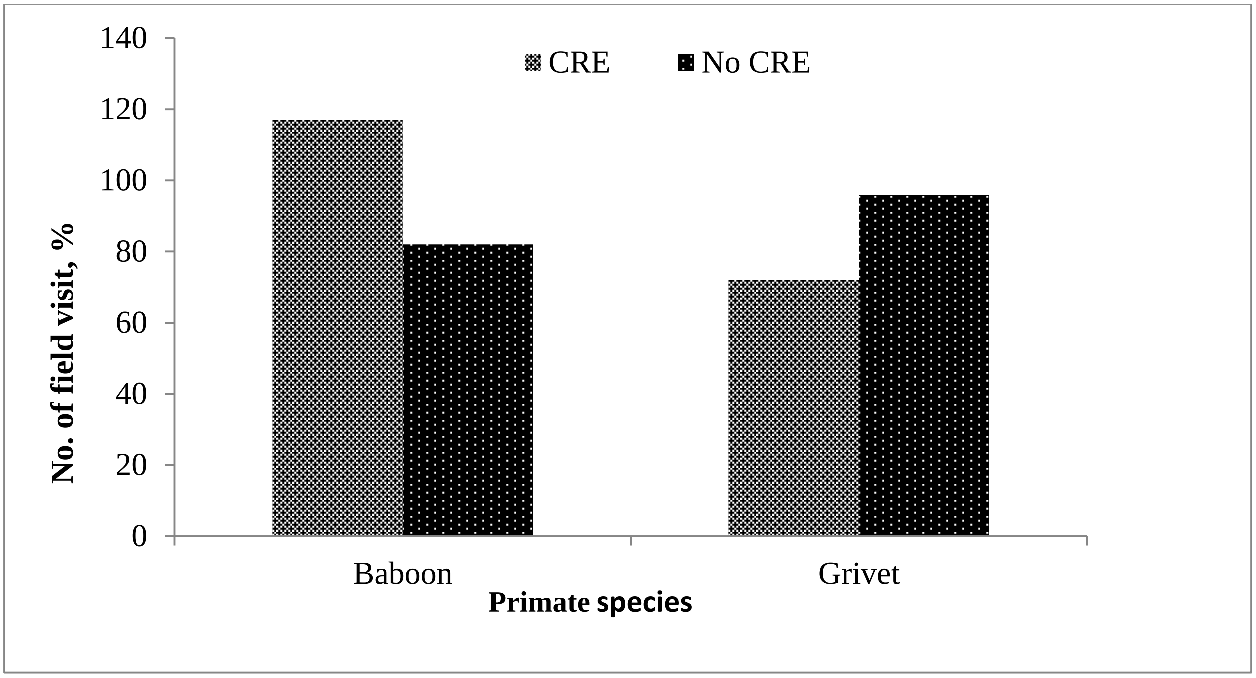

Fig 11. The number of baboon and grivet monkey field visits that did and did not involve crop-raiding events (CRE) on maize fields in the Highlands of Damota Mountain, April to September 2020 and 2021 years (n=367)

Figure Click here to access/download;Figure;Fig 12.docx

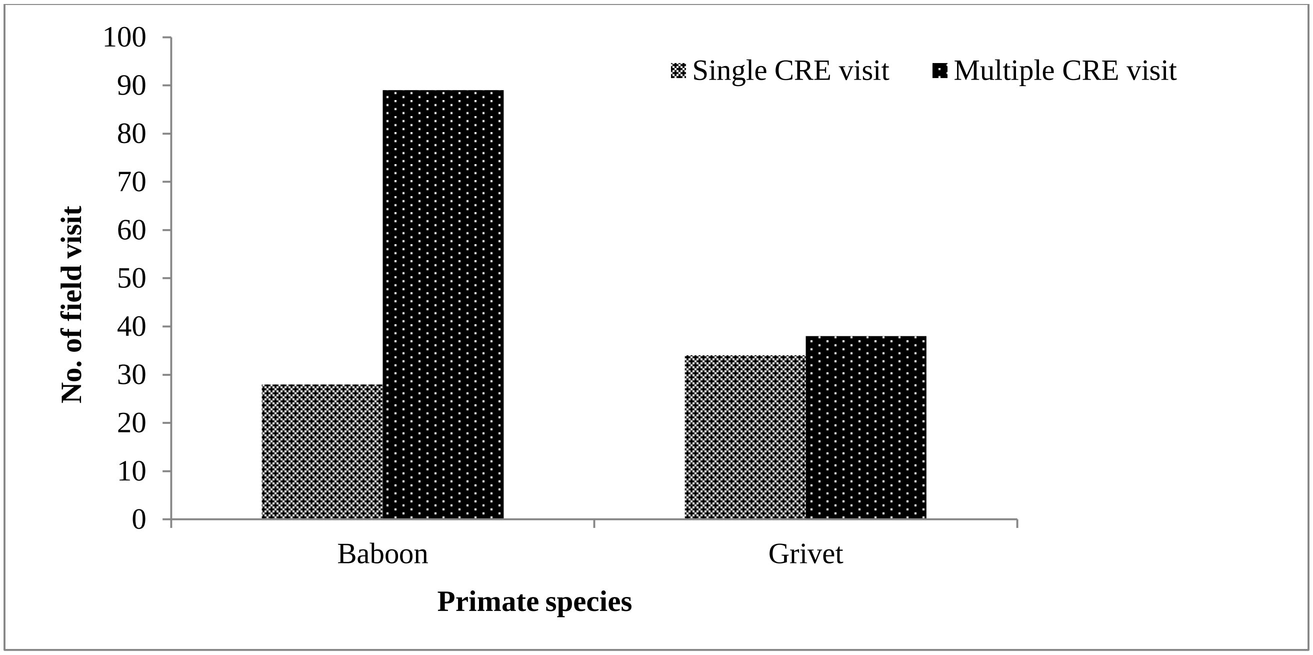

Fig 12. The number of baboon and grivet monkey field visits that involved single- and multi-crop raiding events on maize fields in the Highlands of Damota Mountain, April to September 2020 and 2021 years (n=189).

Click here to access/download
Supporting Information
S1. Fig 4. maize damaged by olive baboons.docx

Click here to access/download
Supporting Information
S2. Fig 5. maize damage by grivet monkeys.docx

Click here to access/download
Supporting Information
S3 Table 4. Primate maize damage by camera traps..docx
