# Supplementary material for: Patterns of primates crop foraging and the impacts on incomes of smallholders across the mosaic agricultural landscape of Wolaita zone, southern Ethiopia
Source: PLoS One. 2024 Nov 18;19(11):e0313831. doi: 10.1371/journal.pone.0313831 (PMC11573158; doi:10.1371/journal.pone.0313831)
Supplement: Attachment — Submitted filename: Comments_DL.docx [file pone.0313831.s018.docx]

Title: Crop Damage by Nonhuman Primates: Quantifying the Keys Parameters of Crop Raiding events on the Livelihoods of Smallholders in an Agriculture- Forest Mosaic Landscape, Wolaita Zone, Southern Ethiopia

Manuscript Number: PONE-D-23-43746

Dear Editor

Thank you so much for inviting me to review this manuscript.

The attempted to understand how the patterns of crop raiding by common baboon and grivet monkey vary with distance from forest edges. For this, the authors has established field experiment in forest areas which are protected, and in not protected. The study is valuable and has added insights further beyond that we can be gain through crop raiding surveying using questionnaires. However, it is unfortunate that the manuscript was not well synthesized as expected. For example, the data analysis is weak in that both fixed factors and random factors were not appropriately considered in the analysis and due to this the study has failed to show strong evidence across spatio-temporal scales across the study landscape. Moreover, I could not understand also why the authors applied different distance gradients in setting up field experiment (only at 50 m distance around protected areas, but at distances of 50 m, 100 m, 200 m, and 300 m from the forest edge at non-protected areas.

If the authors are willing to undertake major revision based on the dataset they have, I am sure the manuscript can come up with novel ideas. However, as it is now, it cannot be appropriate to be considered for publication.

Specific comments

Title: Please make your title concise and precise; it is now unnecessarily long and complex

I would suggest to be modified as: ‘Patterns of Primates’ Crop Raiding and the impacts on incomes of Smallholders across Mosaic agricultural Landscape of Wolaita Zone, Southern Ethiopia’

Introduction: this section is too shallow, lacks strong evidence from bodies of literature. Instead, it seems the description of the study area. Hence, I would recommend major revisions by scanning more literature review. For example, you can refer: Lemessa, D., Hylander, K., & Hambäck, P. (2013). Composition of crops and land-use types in relation to crop raiding pattern at different distances from forests. *Agriculture, Ecosystems and Environment*, *167*, 71–78. https://doi.org/10.1016/j.agee.2012.12.014

Materials and methods

Study area

Please include the ecosystem type and farming systems in the description of the study areas. Refer to this doc regarding the farming system: Amede, T., Auricht, C., Boffa, J.-M., Dixon, J., Mallawaarachchi, T., Rukuni, M., & Teklewold-Deneke, T. (2015). The Evolving Farming and Pastoral Landscapes in Ethiopia: A Farming System Framework for Investment Planning and Priority Setting. September.

Line 146-148, how can famers identify the age categories of the crop raiders? It is not clear!

Experimental setup: you need to show experimental setup by drawing schematic sketch, it is not entirely clear from the texts you wrote??

Data analysis

This is entirely less clear and has a lot of shortfalls. The study was undertaken across a landscape and hence, it is expected that there random factors besides the data considered as fixed factors. The data analysis undertaken did not take into account this issue. This means that such dataset can strongly be analyzed using mixed models such as LMM or GLMM. Now, simple non-parametric statistical tests were used and even are not clearly described how and for what type of response variables they used. For example, Ch-square for what kind of frequency distribution? Mann Whitney test for what type of data? and F-test?

Line 188-190, For the analysis of primate assaults on maize within both preventive and non-preventive maize fields at different seasons and crop phenology, we utilized R-Software [13]. But, what package and function were used is not mentioned.

Moreover, there are statistical tools mentioned in the result but not in data analysis section. Why the authors used two statistical software is also not clear? Hence, I would strongly suggest to run the analysis based on these comments and reciprocally present the results.

Results

I was a bit confused how the authors described the crop damage from maize stems and cobs.

- Is it stems/cob or cobs/stems?

Discussion section

Since some results may be changed after the revision of the analysis, at this stage it is not sensible to comment on this section.
