# Supplementary material for: Patterns of primates crop foraging and the impacts on incomes of smallholders across the mosaic agricultural landscape of Wolaita zone, southern Ethiopia
Source: PLoS One. 2024 Nov 18;19(11):e0313831. doi: 10.1371/journal.pone.0313831 (PMC11573158; doi:10.1371/journal.pone.0313831)
Supplement: Attachment — Submitted filename: Comments PLOS ONE.docx [file pone.0313831.s019.docx]

**Comments**

Dear Editor of PLOS ONE, I appreciate the opportunity to review this manuscript. After reading the MS, I put some comments and suggestions on a separate file. If the raised issues were addressed and amended, it would greatly benefit readers or the larger scientific community.

Best regards,

Teklay

**Comments and suggestions to the Authors**

This paper is good and can be strengthened for your audience to benefit even more from your study. I made some minor comments and suggestions. The authors should use the separate file to correct the suggested comments. In addition, there are some comments and suggestions that require clarification.

**Title:**

1. The title is very long. It should be shorter than the present and to the point accordingly.
2. The title is similar with the work titled “Crop Damage by Primates: Quantifying the Key Parameters of Crop-Raiding Events” in western Uganda and published in PLOS ONE although it is in different countries. Would you modify your title?

**Abstract:**

1. Line -13- “This study aims to assess primates foraging behavior” Is assessing primates foraging behavior was one of the objective of the paper? If yes where is the method and result of the objective?
2. An abstract should show slightly how to collect the data of the work. However, this manuscript didn’t show how to collect the data.

**Introduction**

1. The authors described as the study area contains endemic animal and plant species. Would you please list some of them?
2. The gap of the study should describe at the introduction part. However, I didn’t see a research gap in your introduction section.
3. Line-39- 47- It will be better if this part moved to the description of the study area section.
4. Lines -55- Please remove the method from the introduction part.
5. The introduction part is very short. It didn’t highlight what is done and what is the gap. I recommend writing strong introduction.

**Study area**

1. Line-68- remove the word “see”

**Experimental setup**

1. What do 25 fields mean? Would you clarify in detail?
2. Line -122- “Data collection was carried out by farmers who had received training from researchers”. I doubt about this. How farmers can collect data? The work is on crop damage by Nonhuman Primates. If yes how farmers can collect actual data. I strongly believe that the data collected by farmers might be biased. Why didn’t participate field experts rather than the farmers.
3. Line-127-128- The data collectors (farmers) received incentives. However, this may makes them biased on the data.
4. I have doubt on the data collection method (Farmer observation and reports). Thus, the authors should give scientific evidence on the doubt why they use farmers as data collectors by giving incentives.

**Data analysis**

**Result**

1. Line-213- remove the word “as detailed in”
2. The result of the manuscript is very shallow. Please try to make it strong result.

**Discussion**

1. Line -349- 354- this is a repetition of the result. Please don’t repeat your result in the discussion section. Instead try to discuss your result (the meaning and application of your result).
2. Your discussion is very broad and general. I suggest you to give emphasis on the interpretation of your result and comparing your result with recent publications similar to your work.

**Conclusion**

1. Would you please add the application of your work in the introduction section?
2. Make it strong conclusion based on your findings.

**General Comments**

1. Grammar and structure of sentences should be revised for better improvement.

I hope this helps to make the paper better.

Best wishes.
