# Supplementary material for: Patterns of primates crop foraging and the impacts on incomes of smallholders across the mosaic agricultural landscape of Wolaita zone, southern Ethiopia
Source: PLoS One. 2024 Nov 18;19(11):e0313831. doi: 10.1371/journal.pone.0313831 (PMC11573158; doi:10.1371/journal.pone.0313831)
Supplement: Attachment — Submitted filename: Response to revieweres.docx [file pone.0313831.s020.docx]

**Title**: Crop Damage by Nonhuman Primates: Quantifying the Keys Parameters of Crop Raiding events on the Livelihoods of Smallholders in an Agriculture- Forest Mosaic Landscape, Wolaita Zone, Southern Ethiopia

**Manuscript Number:** PONE-D-23-43746

**Response to Reviewers**

We are great full to the editors and reviewers for their insightful and valuable comments on our paper. We have carefully considered the comments and tried our best to address every one of them. We hope the manuscript after careful revisions meet your higher standard journal. The authors welcome further constructive comments if any. We provided the point by point responses. All modifications in the manuscript have been highlighted in yellow color.

Sincerely,

Yigrem Deneke

**Response to Reviewer 1**

**Title**: Please make your title concise and precise; it is now unnecessarily long and complex

I would suggest to be modified as: ‘Patterns of Primates’ Crop Raiding and the impacts on incomes of Smallholders across Mosaic agricultural Landscape of Wolaita Zone, Southern Ethiopia’

**Response:** The title has been modified to "Patterns of Primates Crop Raiding and the Impacts on Incomes of Smallholders across Mosaic Agricultural Landscape of Wolaita Zone, Southern Ethiopia."

**Introduction**: this section is too shallow, lacks strong evidence from bodies of literature. Instead, it seems the description of the study area. Hence, I would recommend major revisions by scanning more literature review. For example, you can refer: Lemessa, D., Hylander, K., & Hambäck, P. (2013). Composition of crops and land-use types in relation to crop raiding pattern at different distances from forests. *Agriculture, Ecosystems and Environment*, *167*, 71–78. https://doi.org/10.1016/j.agee.2012.12.014

**Response:** The introduction has been revised according to the comments and some points are move to description of the study area.

**Materials and methods**

**Study area**

Please include the ecosystem type and farming systems in the description of the study areas. Refer to this doc regarding the farming system: Amede, T., Auricht, C., Boffa, J.-M., Dixon, J., Mallawaarachchi, T., Rukuni, M., & Teklewold-Deneke, T. (2015). The Evolving Farming and Pastoral Landscapes in Ethiopia: A Farming System Framework for Investment Planning and Priority Setting. September.

**Response:** The introduction has been revised to incorporate the comments:

"The Wolaita zone, characterized by a highland perennial farming system, supports a diverse array of crops (Amede et al., 2017). Primary food crops in this region, as reported by Amede et al. (2017), include maize, teff, various vegetables, and root and tuber species such as cassava, yam, potato, sweet potato, and taro. Additionally, tropical and temperate fruit tree crops like banana, avocado, mango, and apple are cultivated in the Wolaita areas (Amede et al., 2017)."

**Line 146-148, how can famers identify the age categories of the crop raiders? It is not clear!**

**Response:** Most of the farmers had completed secondary school, and some of them had received bachelor's degrees in related fields such as plant science and biology. They possess indigenous knowledge regarding the identification of primate species and age categories of the crop raiders. Additionally, they were trained by the researchers to become more familiar with using scientific methods for identifying the age categories of the crop raiders.

Experimental setup: you need to show experimental setup by drawing schematic sketch, it is not entirely clear from the texts you wrote??

**Response:** I depicted the experimental setup by including a schematic sketch in this manuscript.
**Data analysis**
This is entirely less clear and has a lot of shortfalls. The study was undertaken across a landscape and hence, it is expected that there random factors besides the data considered as fixed factors. The data analysis undertaken did not take into account this issue. This means that such dataset can strongly be analyzed using mixed models such as LMM or GLMM. Now, simple non-parametric statistical tests were used and even are not clearly described how and for what type of response variables they used. For example, Ch-square for what kind of frequency distribution? Mann Whitney test for what type of data? and F-test?

**Response:** We chose a multiple regression model over LMM or GLMM models because it is suitable for analyzing spatio-temporal data of the dependent variable (maize rate of damage) along with multiple independent variables, such as the number of individuals raiding, primate CREs, field distance, duration of raiding, and crop phenology. This statistical model is supported by Wallace and Hill (2012). A chi-square test was employed to examine the variation in the amount of maize damage by primates across different variables, including primate species raiding duration, multiple versus single raid events, primate CFE in time of day, and age-category of raiding in single or group. The Mann-Whitney U test was utilized to compare primate CREs of raiding durations and different age categories of primate species on CREs. Additionally, the F-test was applied to compare estimates of maize damage between preventive and non-preventive strategies, as well as between single and multiple raids.

**Line 188-190**, For the analysis of primate assaults on maize within both preventive and non-preventive maize fields at different seasons and crop phenology, we utilized R-Software [13]. But, what package and function were used is not mentioned. Moreover, there are statistical tools mentioned in the result but not in data analysis section. Why the authors used two statistical software is also not clear? Hence, I would strongly suggest to run the analysis based on these comments and reciprocally present the results.

**Response:** In R software, we utilized the bplot function in the Rlab package. We found that creating graphics such as boxplots is more effective and easier to use for data interpretation compared to SPSS. Therefore, we employed two statistical tests.

**Results**

I was a bit confused how the authors described the crop damage from maize stems and cobs.

- Is it stems/cob or cobs/stems?

**Response:** It was observed that maize stems were damaged and cobs were plucked (stems/cobs).

**Discussion section**

Since some results may be changed after the revision of the analysis, at this stage it is not sensible to comment on this section.

**Response: Ok. It is modified based on results**

**Response to Reviewer 2**

This paper is good and can be strengthened for your audience to benefit even more from your study.

**Response:** Thank you very much.

**Title**

1. The title is very long. It should be shorter than the present and to the point accordingly.
2. The title is similar with the work titled “Crop Damage by Primates: Quantifying the Key Parameters of Crop-Raiding Events” in western Uganda and published in PLOS ONE although it is in different countries. Would you modify your title?

**Response:** It is modified

**Abstract:**

1. Line -13- “This study aims to assess primates foraging behavior” Is assessing primates foraging behavior was one of the objective of the paper? If yes where is the method and result of the objective?
2. An abstract should show slightly how to collect the data of the work. However, this manuscript didn’t show how to collect the data.

**Response:** I have modified it to "primate crop foraging/raiding events (CFE/CRE)." The method is described in the manuscript, and the data collection was conducted through farmer observation and reports, as well as camera traps. The data collection method is also incorporated into the abstract.

**Introduction**

1. The authors described as the study area contains endemic animal and plant species. Would you please list some of them?
2. The gap of the study should describe at the introduction part. However, I didn’t see a research gap in your introduction section.
3. Line-39- 47- It will be better if this part moved to the description of the study area section.
4. Lines -55- Please remove the method from the introduction part.
5. The introduction part is very short. It didn’t highlight what is done and what is the gap. I recommend writing strong introduction.

**Response:** The introduction has been revised based on the comments. The research gap is indicated, and the description of endemic animal and plant species, as well as the detailed description of the study area, has been removed from the manuscript.

**Study area**

1. Line-68- remove the word “see”

**Response: I removed the word “see”**

**Experimental setup**

1. What do 25 fields mean? Would you clarify in detail?
2. Line -122- “Data collection was carried out by farmers who had received training from researchers”. I doubt about this. How farmers can collect data? The work is on crop damage by Nonhuman Primates. If yes how farmers can collect actual data. I strongly believe that the data collected by farmers might be biased. Why didn’t participate field experts rather than the farmers.
3. Line-127-128- The data collectors (farmers) received incentives. However, this may makes them biased on the data.
4. I have doubt on the data collection method (Farmer observation and reports). Thus, the authors should give scientific evidence on the doubt why they use farmers as data collectors by giving incentives.

**Response:** Twenty-five maize fields were selected for this study, comprising 8 protective and 17 non-protective maize fields. Most of the farmers involved had completed secondary school, and some of them had received bachelor's degrees in related fields. The farmers managed their own maize fields during the day and night, drawing on their indigenous knowledge and practices concerning human-wildlife conflict assessments. All these farmer participants in data collection are members of Damota Community Managed Forest, and they are developing knowledge and skills from the forest managers and local NGOs (World Vision of Ethiopia) on how to manage and protect the forest, as well as assessing human-wildlife conflict and mitigating strategies. Additionally, all farmers were trained by the researchers to familiarize themselves more with scientific data collection protocol. Moreover, researchers supervised the data collection process. The farmers received per diem payments for guarding the camera traps during four consecutive trapping nights per month, and they also received compensation fees at the end of the project period.

**Data analysis**

**Result**

1. Line-213- remove the word “as detailed in”
2. The result of the manuscript is very shallow. Please try to make it strong result.

**Response**: I removed the phrase "as detailed in." Additionally, I included an additional result that highlights the strength of using multiple regression models for the analysis.

**Discussion**

1. Line -349- 354- this is a repetition of the result. Please don’t repeat your result in the discussion section. Instead try to discuss your result (the meaning and application of your result).
2. Your discussion is very broad and general. I suggest you to give emphasis on the interpretation of your result and comparing your result with recent publications similar to your work.

**Response:** I have revised this section by eliminating the repetition of the result. Additionally, I compared our findings with those of similar works, including recent publications.

**Conclusion**

1. Would you please add the application of your work in the introduction section?
2. Make it strong conclusion based on your findings.

**Response:** I have described this section based on my findings.

Grammar and structure of sentences should be revised for better improvement.

**Response**: Grammar and structure of sentences have been improved
