# Supplementary material for: Patterns of primates crop foraging and the impacts on incomes of smallholders across the mosaic agricultural landscape of Wolaita zone, southern Ethiopia
Source: PLoS One. 2024 Nov 18;19(11):e0313831. doi: 10.1371/journal.pone.0313831 (PMC11573158; doi:10.1371/journal.pone.0313831)
Supplement: Attachment — Submitted filename: Comment.docx [file pone.0313831.s021.docx]

March 7, 2024

Dear Editor,

Thank you once again for inviting me to review further the manuscript identified by PONE-D-23-43746R1. The authors have now made revisions and enhanced the clarities of their manuscript. However, still the introduction is too shallow or very short at least. Moreover, in the data analysis section, the authors are arguing regarding two aspects:

1. ‘it is easier to make visualizations of the results, e.g. plotting box plot using SPSS instead of R’, but the authors have used R to run lm analysis which is even more difficult when compared to making box plot graph using e.g., boxplot<-boxplot(damage~ locations). This does not convince me and I strongly suggest using R instead of SPSS to produce high quality graph.
2. ‘We chose a multiple regression model over LMM or GLMM models because it is suitable for analyzing spatio-temporal data of the dependent variable (maize rate of damage)’ over the LMM. However, you have random factors which, due to the variations among the sites/seasons unnecessarily inflate the impacts of noises in the model and subsequently the results. To intuitively analyze the data and thereby enhance the novelty of your study, I still insist to run the analysis using LMM which is the extension of lm (that you have used now) to take into account both fixed factors (that you measured or recorded) and the random factors (sites/seasons) that you cannot do this with ordinary linear model.

If he authors willing to revise their manuscript based on these points, I think the paper can be relevant to be published in PLOS ONE journal.
