# Supplementary material for: Patterns of primates crop foraging and the impacts on incomes of smallholders across the mosaic agricultural landscape of Wolaita zone, southern Ethiopia
Source: PLoS One. 2024 Nov 18;19(11):e0313831. doi: 10.1371/journal.pone.0313831 (PMC11573158; doi:10.1371/journal.pone.0313831)
Supplement: Attachment — Submitted filename: Comments PLOS ONE R1.docx [file pone.0313831.s022.docx]

**Comments**

Dear Editor of PLOS ONE, I am so grateful again to review this manuscript. Most of my comments and suggestions raised in the first session are addressed and amended. Accordingly, the manuscript is improved than the previous one. However, I raised few comments and queries again for better improvement of the manuscript.

Best regards,

**Comments and suggestions to the Authors**

Although most of the previous comments and suggestions are corrected, there are still some comments and queries that require improvements and clarification.

**Title**

1. Modified according the comments

**Abstract:**

1. I didn’t see the highlight how to collect the data of the work in the abstract section. Yes, it is described in detail in the MS. However, abstract should highlight the method of data collection.

**Introduction**

1. Corrected and improved.

**Study area**

1. Line 107, According to reference [25], delete the word reference
2. Line 13 and 111, 10x10 meters, correct as 10 x10 meters

**Farmer observation and reports**

1. Farmer? Only one farmer or farmers?
2. Line -141- “The data collection was conducted by twenty five farmers who had received training from researchers…..” Still, this concern is serious. How farmers can collect data? Although they completed secondary school or above, they are already farmers. Thus, how farmers can collect actual data. This may lead to biasness. Why didn’t participate field experts rather than the farmers?
3. Furthermore, the data the farmers received incentives. However, this may makes them biased on the data.
4. Dear authors, you didn’t give a scientific response on the above two serious issues (2 and 3).

**Results**

1. In the results in the testing of significance level, there is writing error. For instance, line 288: (F=292.5, df=11, p < .001, see Figure 5). Here, it should rewrite as follows: (F = 292.5, df = 11, p < .001, see Figure 5). Your writing style should consistency throughout the MS.
2. There is inconsistency in the writing style of the MS. For example, Line 288 (F=292.5, df=11, p < .001, see Figure 5) and line 299 (χ² = 58.62, d.f. = 10, P < 0.05). Here, look the degree of freedom (df). In the first degree of freedom written as df while in the second degree of freedom written as d.f. You have to correct such inconsistencies throughout the manuscript.

General Comments

1. There is inconsistency in the writing style in the MS (as described as the results section in the above).
2. Furthermore, I have observed other inconsistencies in the MS. For instance, line 77 (Fig. 1), line 118 (Figure 2), line 124 (see figure 3), line 280 (Fig. 5), line 285 (as illustrated in Fig. 6), line 295 (as illustrated in Fig 7), line 303 (see Fig 8) …… Please correct these and other inconsistencies throughout the manuscript.

Accordingly, the MS requires serious editing once again before go to the production for publication.
