# Supplementary material for: Patterns of primates crop foraging and the impacts on incomes of smallholders across the mosaic agricultural landscape of Wolaita zone, southern Ethiopia
Source: PLoS One. 2024 Nov 18;19(11):e0313831. doi: 10.1371/journal.pone.0313831 (PMC11573158; doi:10.1371/journal.pone.0313831)
Supplement: Attachment — Submitted filename: Response to revieweres..docx [file pone.0313831.s023.docx]

**Title**: Patterns of Primates Crop Raiding and the impacts on incomes of Smallholders across Mosaic agricultural Landscape of Wolaita Zone, Southern Ethiopia

**Manuscript Number:** PONE-D-23-43746-R3

**Response to Reviewers**

We are great full to the editors and reviewers for their insightful and valuable comments on our paper. We have carefully considered the comments and tried our best to address every one of them. We hope the manuscript after careful revisions meet your higher standard journal. The authors welcome further constructive comments if any. We provided the point by point responses. All modifications in the manuscript have been highlighted in yellow color.

Sincerely,

Yigrem Deneke

**Journal Requirements:**

Please review your reference list to ensure that it is complete and correct. If you have cited papers that have been retracted, please include the rationale for doing so in the manuscript text, or remove these references and replace them with relevant current references. Any changes to the reference list should be mentioned in the rebuttal letter that accompanies your revised manuscript. If you need to cite a retracted article, indicate the article’s retracted status in the References list and also include a citation and full reference for the retraction notice.

**Response**

The following reference was removed from the manuscript in the first-round revision (R1):

- The linear regression model results are reported as R^2^ values (proportion of variance accounted for), beta values (contribution to the model), t statistics (statistical significance of the contribution), and regression equations (the combination of variables best accounting for observed outcomes) (Sokal RR & Rohlf FJ, 1995). This was removed from the data analysis because the linear model only analyzes data with fixed factors. In contrast, the linear mixed model analyzes data with both fixed and random factors, along with the response variables.

The following nine references were added to the manuscript in the second-round revision (R2):

- Hill, CM, Webber, AD, 2010; Hockings, KJ, Sousa, C, 2013; Freed, BZ, 2012; McGuinness, S, Taylor, D, 2014; Findlay, LJ, 2016; Gillingham, S, Lee, PC, 2003; Aharikundira, M, Tweheyo, M, 2011; Ango, TG, Börjeson, L, Senbeta, F, Hylander, K, 2014; Fang L, Hong, Y, Zhou Z, Chen, W, 2021. This is added to the manuscript because it includes important additional background information for this study.

The following reference was removed from the manuscript in the second & third-round revision (R2 &R3):

- R core team. R, 2020. This reference was removed because the data were only analyzed using SPSS in the manuscript.

The following reference was added to the manuscript in the third round revision (R4):

- R core team. R, 2024. I used the latest version of the R program because it is robust for analyzing various datasets. This reference is now included to highlight the effectiveness and reliability of R in handling the spatio-temporal datasets.
- I have replaced Table 5 and text, Figure 5, and Figure 6, which were analyzed using SPSS, with new versions that can be analyzed using the R program.
- I have included the number of field experts and local farmers participated in this study
- I have included a relevant remarks in the conclusion section
- I have included the following supporting documents in the manuscript (R3):
- S2 file. The rate of maize damage by olive baboons in different crop phonological stages was analyzed in both protected and open/control fields using R code.
- S3 file. The rate of maize damage by grivet monkeys in different crop phenological stages was analyzed in both protected and open/control fields using R code.
- S4 table 2. A linear mixed model of the maize damage rate by primates, considering different spatio-temporal variables, was analyzed using R code.

**Response to Reviewer 1**

**Reviewer #1**: Thank you once again for inviting me to review furthers this manuscript. It is clear that the study is interesting and has some novelty. However, I think the authors need strong support for the statistical analysis part. With my previous comments, I was trying to encourage them to analyze their data in a robust way using LMM with the inclusion of random factors using R program instead of SPSS. The authors are stating that they used SPSS to do so and they even took out the R now from their data analysis section with this version. Still how they used the different statistical tools for the analysis is crude and not explicitly described. If the authors are willing to improve their manuscript by including these comments, I think the manuscript may be accepted for the publication without further review process.

**Response:** Thank you very much for the comments. I included the analysis done with R, running the analysis using LMM to account for both fixed and random factors and response variables. This analysis is clearly indicated in the supporting documents. The different statistical tools used for the analysis are also explicitly described in the data analysis section.

**Reviewer #2:** You corrected all the comments and suggestions that I gave you in the second round. Hence, I suggest to the editor that it be published in the journal.
Good luck.

**Response:** Thank you very much.
