# Supplementary material for: Patterns of primates crop foraging and the impacts on incomes of smallholders across the mosaic agricultural landscape of Wolaita zone, southern Ethiopia
Source: PLoS One. 2024 Nov 18;19(11):e0313831. doi: 10.1371/journal.pone.0313831 (PMC11573158; doi:10.1371/journal.pone.0313831)
Supplement: Attachment — Submitted filename: Comments to the authors and editors.docx [file pone.0313831.s026.docx]

This manuscript describes the results of a study of patterns of crop foraging by primate species and the impacts on the incomes of smallholders across the mosaic agricultural Landscape of Wolaita Zone.in Ethiopia. I recognize the hard work it has taken to conduct the fieldwork presented in this paper and the time authors have put into analyzing their data and writing the manuscript. However, the manuscript has several conceptual and analytical problems. The introduction lacks conceptual ideas. It should be seriously rewritten on the current Knowledge of crop foraging by wild animals worldwide. Therefore, the paper needs major revision to be accepted by PlOS ONE. It should be written based on the guidelines of PlOS ONE.

I included all my comments within the PDF file. The authors should use this file to correct those comments. I stated some crucial comments here.

1.	The authors use a lot of statistical packages. I advise the authors to consult a statistician about their data for better analysis.

2.	Some of the statistical values are not correct and clear too.

3.	Why did the authors use two different kinds of statistical software? SPSS and R

4.	Once the authors abbreviate phrases, they should use that abbreviation afterward. It is unnecessary to abbreviate here and there.

5.	The author should say crop foraging instead of crop raiding as well as forage instead of raid throughout the paper

6.	The authors should use a similar common name for baboons; olive baboons instead of anubis baboons

7.	The method lacks clarity

8.	I am so unhappy with the citation system. The authors seem no experience or they are careless. For example, a study conducted by, according to a paper by, as stated in reference

9.	There are many tables and figures. Some of them are unnecessary. Please trim your study as short and understandable as possible.
