# Supplementary material for: Patterns of primates crop foraging and the impacts on incomes of smallholders across the mosaic agricultural landscape of Wolaita zone, southern Ethiopia
Source: PLoS One. 2024 Nov 18;19(11):e0313831. doi: 10.1371/journal.pone.0313831 (PMC11573158; doi:10.1371/journal.pone.0313831)
Supplement: Attachment — Submitted filename: Reviewer report_PONE.docx [file pone.0313831.s029.docx]

**Review report**

Review report on Manuscript: “Patterns of Primates Crop Raiding and the impacts on incomes of Smallholders across Mosaic Agricultural Landscape of Wolaita Zone, Southern Ethiopia”

The paper falls well within the scope of the journal, as it addresses pertinent issues related to primate conservation and behavior, which are central themes for the journal's readership. While the paper addresses a crucial issue, a few areas require slight briefing to enhance clarity and academic rigor. Although I have provided specific comments directly within the manuscript (highlighted in yellow), I have also listed few issues below that require attention before publication.

**Abstract:** The author stated that “…..while guarding is assumed to be an efficient protective strategy…when not implemented continuously.” What does it mean?? I don’t think it is scientific as such. You should be clear with the definition of guarding? Unless it is continuous keeping of crop fields in accordance with the animals’ activity time, it will not be guarding. So, rephrase this statement???

**Keywords/Phrases**: Most journals require keywords to be listed alphabetically and they should be chosen from the abstract itself and should not be identical to words used in the title.

**Introduction**

Page 3 Paragraph 2: the author hypothesized that ….frequency of crop raiding by large wild primates …..crop damage decrease with the distance from the forest edges ..” The hypothesis assumes a linear or predictable relationship between distance from the forest edge and primate behavior. Although the hypothesis presents a reasonable starting point, it simplifies complex ecological and behavioral relationships. For instance, primates might not always follow such a pattern due to factors like availability of food in the forest, group dynamics, and habituation to human activity, the size of the crop field, types of crops, seasonal variations, and weather conditions could also impact raiding behavior. However, the authors did not mention these factors in the hypothesis and overemphasized on distance.

**Methods**

Page 5 Paragraph 3: The authors stated that they established 10 maize plots within 50 meters of the forest, while only 15 plots were set up at three different distance ranges: 100 meters, 200 meters, and 300 meters (with 5 plots per range). This means that the plots closest to the forest (at 50 meters) were twice of the number of plots to those in the subsequent distance ranges (100 meters, 200 meters, and 300 meters). Consequently, it is possible that a higher incidence of crop raiding might be observed on these more number of plots closest to the forest. This uneven distribution of plots could potentially lead to biased conclusions. Thus, caution should be taken.

Page 5 Paragraph 4: The authors stated that “... we planted the high-yielding maize variety …” Did authors engage in planting maize seeds to the study plots???

**Data analysis**

Is there any scientific reason to use the old version of the SPSS software (16)??

**Results**

The result section is staffed with large sized tables and too many figure. I recommend revising the larger tables (Table 1 and 3) or consider them for supplementary attachments.

**Discussion**

This section is well presented.

**Conclusion**

The conclusion is a short remarking of the key findings where the authors provided a concise overview of the main findings.
