# Supplementary material for: Patterns of primates crop foraging and the impacts on incomes of smallholders across the mosaic agricultural landscape of Wolaita zone, southern Ethiopia
Source: PLoS One. 2024 Nov 18;19(11):e0313831. doi: 10.1371/journal.pone.0313831 (PMC11573158; doi:10.1371/journal.pone.0313831)
Supplement: Attachment — Submitted filename: Response to revieweres.docx [file pone.0313831.s030.docx]

**Title**: Patterns of Primates Crop Foraging and the impacts on incomes of Smallholders across Mosaic Agricultural Landscape of Wolaita Zone, Southern Ethiopia

**Manuscript Number:** PONE-D-23-43746-R4

**Response to Reviewers**

We are great full to the editors and reviewers for their insightful and valuable comments on our paper. We have carefully considered the comments and tried our best to address every one of them. We hope the manuscript after careful revisions meet your higher standard journal. The authors welcome further constructive comments if any. We provided the point by point responses. All modifications in the manuscript have been highlighted in yellow color.

Sincerely,

Yigrem Deneke

**Journal Requirements:**

Please review your reference list to ensure that it is complete and correct. If you have cited papers that have been retracted, please include the rationale for doing so in the manuscript text, or remove these references and replace them with relevant current references. Any changes to the reference list should be mentioned in the rebuttal letter that accompanies your revised manuscript. If you need to cite a retracted article, indicate the article’s retracted status in the References list and also include a citation and full reference for the retraction notice.

**Response**

The following four references were added to the manuscript in the fourth-round revision (R4):

- Cuesta Hermira, AA, Michalski, F, 2022; Kifle, Z., Bekele, A, 2020; Kifle, Z., Bekele, A, 2021; [Mesfin Matusal](https://onlinelibrary.wiley.com/authored-by/Matusal/Mesfin) et al., 2023. This is added to the manuscript because it includes important additional background information for this study.

The following reference was removed from the manuscript in the fourth-round revision (R4):

- Hansen, LK, 2003.

I have moved the following previously reported results to the supporting information section of the manuscript (R4):

- S1 Fig. Location map of the study area (created with ESRI ArcGIS Desktop 10.8)
- S2 Fig. Various prevention strategies (Wire mesh (A), Human guardian tower (B), Scarecrow (C), Thorny bush (D)) were assessed in eight experimental maize field sites to evaluate their effectiveness in deterring crop raiders. The study was conducted in maize field sites located in Gurumu Woide and Kokate Marachare (Photo credit: Yigrem Deneke).
- S3 Fig. Diagrammatic example of a field map used by observers. HSE = house. GH = guard hut. SH = storage hut. Solid black lines = field boundary. Green objects = trees.
- S4 Fig. The images above depict camera trap captures of various wildlife species observed in maize field sites located in Damota Mountain, Southern Ethiopia: (A) olive baboons (*Papio anubis*) (B) Grivet monkeys (*Chlorocebus aethiops*), (C) Porcupine (*Hystrix cristata*), and (D) Bushbuck (*Tragelaphus scriptus*)
- S5 Fig. Relative frequency of raid durations by primate CREs (n = 95).
- S6 Fig. Relative frequency of raiding by primate CREs (n = 95)
- S7 Fig. The number of baboon and grivet monkey field visits that did and did not involve crop-raiding events (CRE) on maize fields in the Highlands of Damota Mountain, April to September 2020 and 2021 years (n=367)
- S8 Fig. The number of baboon and grivet monkey field visits that involved single- and multi-crop raiding events on maize fields in the Highlands of Damota Mountain, April to September 2020 and 2021 years (n=189).
- S1 Table. Maize field and study plot size on the protective and non-protective maize fields
- S2 Table. Farmer observation and reported of maize damage assessments (580 maize stem expected per plot except field no. 25 (see the text)

**Response to Reviewer #1**

**Abstract:** The author stated that “…..while guarding is assumed to be an efficient protective strategy…when not implemented continuously.” What does it mean?? I don’t think it is scientific as such. You should be clear with the definition of guarding? Unless it is continuous keeping of crop fields in accordance with the animals’ activity time, it will not be guarding. So, rephrase this statement???

**Response:** It has been revised and properly rephrased.

**Keywords/Phrases**: Most journals require keywords to be listed alphabetically and they should be chosen from the abstract itself and should not be identical to words used in the title.

**Response:** It has been corrected and listed alphabetically.

**Introduction:** Page 3 Paragraph 2: the author hypothesized that ….frequency of crop raiding by large wild primates …..crop damage decrease with the distance from the forest edges ..” The hypothesis assumes a linear or predictable relationship between distance from the forest edge and primate behavior. Although the hypothesis presents a reasonable starting point, it simplifies complex ecological and behavioral relationships. For instance, primates might not always follow such a pattern due to factors like availability of food in the forest, group dynamics, and habituation to human activity, the size of the crop field, types of crops, seasonal variations, and weather conditions could also impact raiding behavior. However, the authors did not mention these factors in the hypothesis and overemphasized on distance.

**Response:** It has been revised.

**Methods**

Page 5 Paragraph 3: The authors stated that they established 10 maize plots within 50 meters of the forest, while only 15 plots were set up at three different distance ranges: 100 meters, 200 meters, and 300 meters (with 5 plots per range). This means that the plots closest to the forest (at 50 meters) were twice of the number of plots to those in the subsequent distance ranges (100 meters, 200 meters, and 300 meters). Consequently, it is possible that a higher incidence of crop raiding might be observed on these more number of plots closest to the forest. This uneven distribution of plots could potentially lead to biased conclusions. Thus, caution should be taken.

Page 5 Paragraph 4: The authors stated that “... we planted the high-yielding maize variety …” Did authors engage in planting maize seeds to the study plots???

**Response:** It has been revised. We compared maize damage assessments at varying distances by evaluating an open maize field located 50 meters from the forest edge, along with individual fields situated 100 meters, 200 meters, and 300 meters away from the forest edge.

**Data analysis**

Is there any scientific reason to use the old version of the SPSS software (16)??

**Response:** We used SPSS Version 16 because the results are consistent with those from more recent versions. The core statistical algorithms have remained largely unchanged across different releases. Key statistical computations in SPSS, such as the Chi-square test, Mann-Whitney U test, Spearman’s Rank Correlation Coefficient, t-test, one-way ANOVA, and F-test, have not significantly differed between versions. This means that when using SPSS Version 16 for standard statistical analyses, the results should match those obtained from more recent versions like SPSS 28, provided the same data and settings are used. The underlying statistical algorithms for these common tests have remained stable, so outputs such as p-values, confidence intervals, and coefficients will be consistent across versions under the same conditions

**Results**

The result section is staffed with large sized tables and too many figure. I recommend revising the larger tables (Table 1 and 3) or consider them for supplementary attachments.

**Response:** We used 5 tables and figures in the Results section; the remaining Table 1 and Table 3 have been moved to the supporting information.

**Discussion**

This section is well presented.

**Response:** Thank you very much

**Conclusion**

The conclusion is a short remarking of the key findings where the authors provided a concise overview of the main findings.

**Response:** Ok

**Response to Reviewer #2**

**Introduction**

The introduction lacks conceptual ideas. It should be seriously rewritten on the current Knowledge of crop foraging by wild animals worldwide.

**Response:** It has been revised based on the current research on crop foraging by primates worldwide

I included all my comments within the PDF file. The authors should use this file to correct those comments. I stated some crucial comments here.

1.	The authors use a lot of statistical packages. I advise the authors to consult a statistician about their data for better analysis.

2.	Some of the statistical values are not correct and clear too.

**Response**: Some of the statistical values have been revised and refined all the analysis throughout the manuscript. We consult a statistician for some analysis.

3.	Why did the authors use two different kinds of statistical software? SPSS and R

**Response**: We use both SPSS and R for our analyses. SPSS is ideal for users who prefer a point-and-click interface and need to perform standard statistical analyses without learning to code. It is well-suited for researchers conducting routine analyses that do not require extensive customization. On the other hand, R is especially useful for handling complex data analysis tasks, such as spatio-temporal ecological datasets. It supports advanced statistical techniques (e.g., linear mixed models with random and fixed effects) and offers highly customizable visualizations (e.g., box plots, bar charts), providing far greater flexibility than SPSS.

4.	Once the authors abbreviate phrases, they should use that abbreviation afterward. It is unnecessary to abbreviate here and there.

**Response**: It has been revised.

5.	The author should say crop foraging instead of crop raiding as well as forage instead of raid throughout the paper

**Response**: It has been revised and corrected throughout the manuscript.

6.	The authors should use a similar common name for baboons; olive baboons instead of	anubis baboons

**Response**: It has been revised to consistently use 'olive baboons' throughout the manuscript.

7.	The method lacks clarity

**Response**: It has been revised, and this section is now clearly written.

8.	I am so unhappy with the citation system. The authors seem no experience or they are careless. For example, a study conducted by, according to a paper by, as stated in reference

**Response**: It has been revised throughout the manuscript.

9.	There are many tables and figures. Some of them are unnecessary. Please trim your study as short and understandable as possible.

**Response**: It has been revised. We used 5 tables and 5 figures in the Results section

**In addition:**

Please ensure that towards the end of the abstract, you indicate the significant potential application of your findings

**Response**: The significant potential of this study has been included in the manuscript.

To the declaration section on ethical statement, please add/confirm explicitly that: “In addition, there was no direct interaction between field personnel and the subjects (the primates) in such a way as to harm the animals or interfere with their freedom in nature such as by way of capture or trapping”

**Response**: It has been incorporated in Ethics statement of the manuscript.

Several subsections within Results and Discussion sections could be consolidated and merged so as to yield a maximum of no more than 4 subsections in each of them. This will make for easier reading

**Response**: All the Results and Discussion sections have been consolidated, merging several headings into four main subsections.

Please try and pay much more close attention to the quality or writing in terms of language, grammar, spelling and logical flow within paragraphs and the interconnections amongst paragraphs and sections. Seek the services of a professional English language Editor.

**Response:** We have improved the language, grammar, spelling, and logical flow within paragraphs and sections throughout the manuscript.

The formatting of the reference list and figure as well as table captions and legends must strictly follow the guidelines of PLOSONE. If not, this alone will lead to rejection of the paper, which would be unfortunate, given the considerable time and effort the authors already invested in revising the manuscript so far

**Response:** It has been revised, and the manuscript has been written according to the PLOS ONE guidelines.

Consolidate and minimize the number of illustrations: Aim for a maximum of 5 tables and 5 figures, and provide information regarding the extra illustrations within the text or leave them out altogether

**Response:** We used 5 tables and 5 figures in the Results section, with the remaining tables and figures included in the supporting information.

Take note that the reviewers have uploaded PDFs of the manuscript in which they have indicated additional specific comments, and that you need to look at these and address all such issues in your revision.

**Response:** We have addressed and carefully corrected both the tracked changes and comments in the PDF made by the reviewers, providing point-by-point responses to each comment.

Finally, we would like to thank all the reviewers and editors for their comments and questions, which have greatly contributed to improving the quality of the paper.
