# Supplementary material for: Patterns of primates crop foraging and the impacts on incomes of smallholders across the mosaic agricultural landscape of Wolaita zone, southern Ethiopia
Source: PLoS One. 2024 Nov 18;19(11):e0313831. doi: 10.1371/journal.pone.0313831 (PMC11573158; doi:10.1371/journal.pone.0313831)
Supplement: Attachment — Submitted filename: Response to revieweres.docx [file pone.0313831.s031.docx]

**Title**: Patterns of Primates Crop Foraging and the Impacts on Incomes of Smallholders across the Mosaic Agricultural Landscape of Wolaita Zone, Southern Ethiopia

**Manuscript Number:** PONE-D-23-43746-R5

**Responses to reviewers**

We are grateful to the editors and reviewers for their insightful and valuable comments on our paper. We have carefully considered the comments and tried our best to address every one of them. We hope the manuscript after careful revisions meets your higher standard journal. The authors welcome further constructive comments if any. We provided the point-by- point responses. All modifications in the manuscript have been highlighted in yellow.

Sincerely,

Yigrem Deneke

**Journal Requirements:**

Please review your reference list to ensure that it is complete and correct. If you have cited papers that have been retracted, please include the rationale for doing so in the manuscript text, or remove these references and replace them with relevant current references. Any changes to the reference list should be mentioned in the rebuttal letter that accompanies your revised manuscript. If you need to cite a retracted article, indicate the article’s retracted status in the References list and also include a citation and full reference for the retraction notice.

**Response**

The following four references were added to the manuscript in the fourth-round revision (R4):

- Naughton-Treves L, Treves A. 2005
- Alemu M, Berihun DC, Lokossou J, Yismaw, B. 2024
- Abate T, Shiferaw B, Menkir A, Wegary D, Kebede Y, Tesfaye K, Keno T*.* 2015
- Prasanna BM, Palacios-Rojas N, Hossain F, Muthusamy V, Menkir A, Dhliwayo T, Ndhlela T, San Vicente FM, Nair SK, Vivek BS, Zhang X, Olsen M, Fan, X. 2020
- Ikhuluru WE, Imboma ME, Liseche SE, Milemele MJ, Shilabiga SD, Cords M. 2023
- Koirala S, Garber PA, Somasundaram D, Katuwal HB, Ren B, Huang C, Li M. 2021
- Hill CM. 2020
- Wiafe ED. 2019
- Mwakatobe A, Nyahongo J, Janemary NJ, Røskaft E. 2014
- Jaleta M, Tekalign W. 2023
- Masha M, Yirgu T, Debele M, Belayneh M. 2021
- Abendroth LJ, Elmore RW, Boyer MJ, Marlay SK. 2011
- Wolaita District Agricultural Office (WDAO). 2021. This is added to the manuscript because it includes important additional background information for this study.

**Responses to reviewers**

**Abstract**

- It needs a brief statement of the study’s objectives and a brief outline of the general methods used to collect information. Please ensure that in the first part of the abstract, there is something (1-2 sentences)outlining how the study was conducted, before mentioning the findings

**Response:** It has been revised accordingly

**Introduction**

- It needs to be expanded with more literature review of recent studies on crop damage by primates in the tropics generally but in Africa in particular (especially maize). Ensure you mention how your study fills in any gap left by such studies i.e, what is new about your findings. This was clearly emphasized by Reviewer #1 but the authors have not addressed it. Many studies related to this have been conducted in many parts of the tropics. The introduction part needs to be at least 2 pages long
- It needs a statement towards the end, of hypotheses to justify linear modeling. Mention at least one hypothesis and what you expected to find out from the study
- Remove the statement in which insects are mentioned as crop raiders. This study is bout primates

**Response:** The manuscript has been revised to include an expanded literature review on recent studies of crop damage caused by primates in tropical regions, with a particular focus on Africa and maize. We have addressed the gap in previous research and highlighted the novel aspects of our findings. Additionally, we incorporated a hypothesis involving linear mixed modeling and removed references to insect-related crop damage.

**Tables and figures**

- Remove vertical lines in tables
- Within tables, center all texts and numbers within columns and rows except column titles
- Standardize font types and size in all tables according to journal guidelines
- Table legends and figure captions must contain enough details to be able to stand alone
- If abbreviations are used in figure axes such as in Fig 5, they must be fully defined in the captions text

**Response:** The manuscript has been revised to ensure that the font types and sizes in all tables adhere to the PLOS ONE guidelines. The table legends and figure captions have been modified to stand alone, and the caption text for Figure 5 has also been corrected.

**In general**

- The language is still below par, and needs professional editing for English. (There are very many affordable but effective ones available ones which can be contacted online to complete editing within 48 hours). Therefore, in the revised edition of the MS, a professional English proof reading certificate must be submitted.
- Only the first word of each title or subtitle should be capitalized
- For decimal numbers, standardize  and round off to a maximum of 3 decimal points
- Throughout the manuscript, adopt a third-party  stance in presentation of the write-up  e’g “the study was conducted …” instead of “We conducted the study …”;  and “The experimental set up … “ instead of “Our experimental set up …”
- Do not rush to resubmit the revised manuscript, if these issues are nit addressed. You have until to end of October 2024.

**Response:** The manuscript has been revised with professional English editing, and the editor certificate is included as supporting information. All other issues have been thoroughly addressed, resulting in significant improvements to the manuscript.

**Comments about the PDF and word documents**

**Question # 1**. Is there any difference between CRE and CFE? If not why didn’t take one of them.

**Response:** This is a valid point raised by the reviewer. The two terms differ in context: Actual crop feeding events (CFEs) refer to instances where primates actively enter a field and feed on crops, including observations of animals consuming leaves, fruits, seeds, and other plant parts. In contrast, potential crop raiding events (CREs) refer to situations where primates enter or approach a field but do not necessarily feed on the crops. In these cases, the animals may trample the field, cause stem or root damage, investigate, or simply pass through without consuming any crops. By categorizing both actual CFEs and potential CREs under the umbrella of crop foraging events, we can explore the overall interaction of primates with agricultural systems, capturing both active feeding behaviors and exploratory actions that may lead to crop damage.

**Question # 2**. Your respondents are too small in number. Why? Are they enough for this study

**Response:** Our experimental study involved 25 maize plots, with one farmer assigned to each plot (a total of 25 farmers) to conduct field observations and record maize damage caused by primates. This arrangement effectively minimized disturbances by primates and allowed for more accurate assessments of primate-related maize damage. The number of farmers was adequate, as the primates had easy access to the fields. The selected farmers, who lived in the surrounding Damota community-managed areas, were long-time residents with extensive experience in managing human-primate conflicts, particularly related to crop damage. As members of the Damota Forest Protection group, they provided valuable insights, enabling us to gather comprehensive information on the patterns and extent of crop foraging events by primates.

All the other comments in the track changes (PDF) and the Word documents have been thoroughly revised and addressed in the manuscript.

Finally, we would like to thank all the reviewers and editors for their comments and questions, which have greatly contributed to improving the quality of the paper.
